# A Pan-Inhibitor for Protein Arginine Methyltransferase Family Enzymes

**DOI:** 10.3390/biom11060854

**Published:** 2021-06-08

**Authors:** Iredia D. Iyamu, Ayad A. Al-Hamashi, Rong Huang

**Affiliations:** 1 Department of Medicinal Chemistry and Molecular Pharmacology, Institute for Drug Discovery, Center for Cancer Research, Purdue University, West Lafayette, IN 47907, USA; iiyamu@purdue.edu (I.D.I.); aalhamas@purdue.edu (A.A.A.-H.); 2 Department of Pharmaceutical Chemistry, College of Pharmacy, University of Baghdad, Bab-almoadham, Baghdad 10047, Iraq

**Keywords:** protein arginine methyltransferases, inhibitor, methyltransferase, competitive inhibitor

## Abstract

Protein arginine methyltransferases (PRMTs) play important roles in transcription, splicing, DNA damage repair, RNA biology, and cellular metabolism. Thus, PRMTs have been attractive targets for various diseases. In this study, we reported the design and synthesis of a potent pan-inhibitor for PRMTs that tethers a thioadenosine and various substituted guanidino groups through a propyl linker. Compound II757 exhibits a half-maximal inhibition concentration (IC_50_) value of 5 to 555 nM for eight tested PRMTs, with the highest inhibition for PRMT4 (IC_50_ = 5 nM). The kinetic study demonstrated that II757 competitively binds at the SAM binding site of PRMT1. Notably, II757 is selective for PRMTs over a panel of other methyltransferases, which can serve as a general probe for PRMTs and a lead for further optimization to increase the selectivity for individual PRMT.

## 1. Introduction

Protein arginine methyltransferases (PRMTs) are a group of enzymes that methylate the guanidino group of the arginine using *S*-adenosyl methionine (SAM) as a methyl donor, producing mono- or dimethylated arginine residues and *S*-adenosyl-*L*-homocysteine (SAH). To date, nine human PRMT members have been identified, which are classified into three types according to the characteristic of their products [1,2,3]. Type I PRMTs include PRMT1, 2, 3, 4, 6, and 8 that produce asymmetric dimethylated arginine, while type II PRMTs consist of PRMT5 and PRMT9 that generate symmetric dimethylated arginine. As the only member in Type III, PRMT7 solely produces monomethylated arginine. Arginine methylation regulates diverse biological processes, including signal transduction, RNA splicing, DNA repair, cell proliferation, and differentiation [4]. Consequently, aberrant levels of PRMTs have been implicated in diverse diseases including cardiovascular diseases, cancers, inflammatory diseases, and diabetes [1,5,6,7,8].

**Figure 1:**
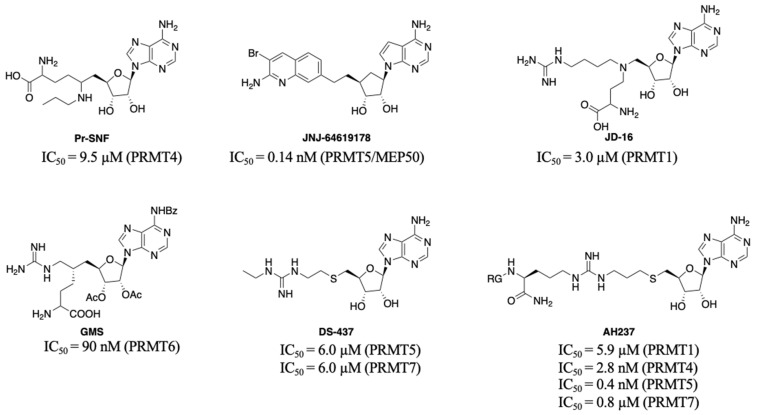
Representative structures of bisubstrate inhibitors of PRMTs.

PRMTs inhibitors have served as valuable tools to probe the biological roles of PRMTs and even led to the development of potential therapeutic agents. Besides allosteric inhibitors for PRMT3 and PRMT5 [9,10], three different types of inhibitors including bisubstrate analogues, substrate, and SAM-competitive inhibitors have been reported [11,12,13,14]. Generally, it is challenging to obtain highly selective inhibitors that competitively bind to the SAM binding site. For example, Pr-SNF, a derivative of sinefungin, was developed as a PRMT4 inhibitor but it has a higher binding affinity for SETD2 (Figure 1) [15]. However, a SAM analogue JNJ-64619178 is a selective and potent PRMT5 inhibitor that is orally bioavailable and has been developed for advanced solid tumors and non-Hodgkin lymphoma [16]. 

Another strategy to develop selective inhibitors is to design bisubstrate analogues that fully or partially occupy both binding pockets. For instance, 5′-(diaminobutyric acid)-*N*-iodoethyl-5′-deoxyadenosine ammonium hydrochloride (AAI) a SAM analogue has been used with PRMT1 to generate its bisubstrate inhibitors in situ [17,18]. In addition, JD-16 attached a terminal guanidine to a SAM analogue through a butyl linker, displaying selectivity for PRMT1 (IC_50_ = 3.0 µM) versus PRMT4 (Figure 1) [19,20]. GMS is a bisubstrate inhibitor for PRMT6 with an IC_50_ of 90 nM and DS-437 is a dual PRMT5/7 inhibitor with IC_50_ of 6.0 µM against both enzymes [21,22]. Recently developed AH237 is a potent and dual PRMT4/5 inhibitor by tethering a thioadenosine with an RGK tripeptide through a guanidino group [23]. Inspired by AH237, we aim to develop potent pan-inhibitors against all PRMTs, which can be further optimized to selective inhibitors for individual PRMT in the future.

## 2. Design

**Figure 2:**
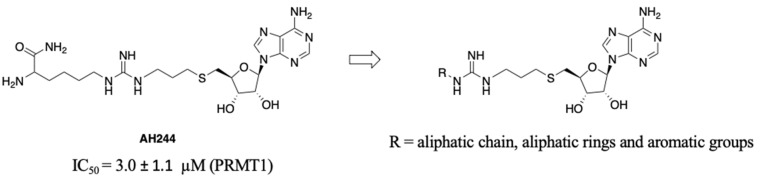
Design of pan-PRMT inhibitors.

SAM is a common cofactor that donates the methyl group to most methyltransferases. Thus, SAM has served as a lead compound to develop SAM-competitive inhibitors. One successful example is the PRMT5 inhibitor JNJ-64619178 [24]. Besides, SAM mimic has served as a building block in the development of bivalent or bisubstrate analogues to tune the selectivity by incorporating different substrate moieties specific to the methyltransferase of interest [17,25]. Recently, we have reported a series of bisubstrate analogues by linking amino acid residues or peptides to a SAM analogue through a guanidino group, exhibiting selectivity for PRMTs over protein lysine methyltransferases (PKMTs) and protein N-terminal methyltransferase 1 (NTMT1) [23]. Among them, AH237 is highly selective for PRMT4/5 among 41 methyltransferases, displaying an IC_50_ value of 2.8 and 0.42 nM for PRMT4 and PRMT5, respectively [23]. As AH237 has limited application for cell-based studies due to its low cell penetration, we focus on the replacement of its peptide moiety with lipophilic groups to improve permeability. The thioadenosine and guanidino moiety were retained to preserve the selectivity for PRMTs over other methyltransferases. Our previous studies on PRMT inhibitors revealed that a propyl linker between the sulfur atom and the guanidino group is optimal and modulates the binding affinity [23]. Thus, we chose to keep the 3-C atom linker as the optimal length between the thioadenosine and guanidino group [23]. Interestingly, bisubstrate analogues containing a propyl linker showed a twofold increased potency for PRMT1 when the substrate moiety changed from a tripeptide to a single amino acid [23]. For example, AH244 showed an IC_50_ value of 3.0 µM for PRMT1 (Figure 2) [23]. Thus, we hypothesize that replacing the amino acid portion with an aliphatic or aromatic group would retain the potency to PRMT1. To test our hypothesis, we designed compounds to investigate the effect of aliphatic and aromatic substitution on PRMT inhibition (Figure 2). 

## 3. Synthesis

**Scheme 1:**
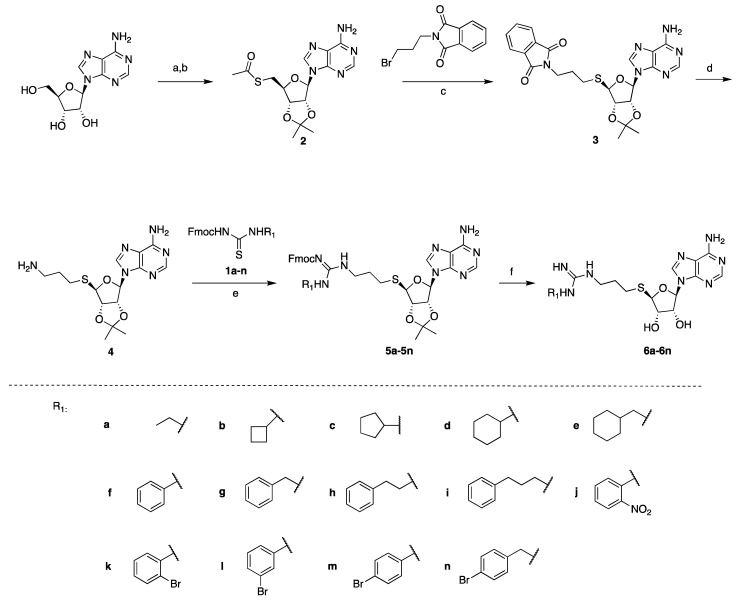
Synthesis of compounds **6a**–**6n**. Reagents and conditions: (**a**) *p*TsOH, acetone, 87%, (**b**) thioacetic acid, PPh_3_, DIAD, THF, 94%, (**c**) NaOCH_3_, MeOH 63%, (**d**) hydrazine, MeOH, 81%, (**e**) EDC, DIPEA, CH_2_Cl_2_, 53–79%, (**f**) piperidine, HOBt, DMF, then TFA, H_2_O, 47–65%.

The thiourea intermediates (**1a**–**1n**) were synthesized from the reaction of commercially available amines with Fmoc protected isothiocyanates [23]. These amines were selected to explore the effects of different lengths, ring sizes, and substituents on the inhibition of PRMT activity. The thioadenosine intermediate was synthesized as previously described [23]. The diol of commercially available adenosine was first protected before a Mitsunobu reaction with thioacetic acid to afford the thioester **2** [23]. The hydrolysis of the thioester and subsequent thiol alkylation with phthalimide alkyl bromide produced the phthalimide **3** [23]. The removal of the phthalimido group by hydrazine afforded the free amine intermediate **4**, which was coupled with different thioureas (**1a**–**1n**) to afford **5a**–**5n** [23]. The deprotection of the Fmoc group with piperidine followed by acid-catalyzed deprotection of the acetal group afforded the final compounds **6a**–**6n** (Scheme 1).

## 4. Biochemical Characterization 

First, we evaluated the inhibitory activities of all synthesized compounds for PRMT1 in a SAH hydrolase (SAHH)-coupled fluorescence assay under the condition of the *K*_m_ values of both SAM (10 µM) and the H4-21 substrate peptide (5 µM) [23]. For those potent compounds that displayed an IC_50_ value lower than half of the enzyme concentration, we re-determined their IC_50_ values under the condition of 4 Km values of both SAM and H4-21 peptide. Destruction of the alpha-amino acid moiety of AH244 (IC_50_ = 3.0 ± 1.1 µM) yielded an ethyl group substitution to produce **6a** (IC_50_ = 0.62 ± 0.08 µM), exhibiting about fivefold increased inhibition for PRMT1 compared to the parent compound AH244 (Table 1). Substitution of the ethyl group with a cyclobutane yielded **6b** that greatly improved the inhibitory activity, however, further increase in the ring size to cyclopentane and cyclohexane led to slight reduction in the inhibitory activity. Incorporating a methylene group between the cyclohexane and guanidino group allowed certain flexibility but did not change the inhibitory activity, as reflected by **6d** and **6e**.

**Figure 3:**
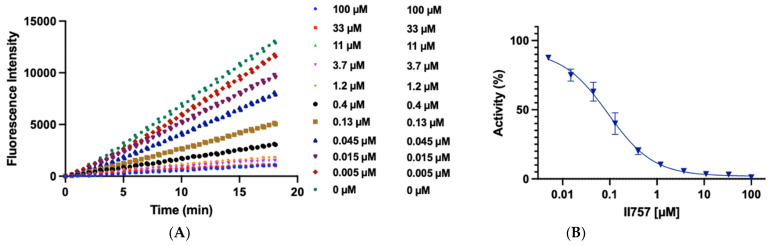
Biochemical characterization of compound **II757** (**6l**). (**A**) SAHH-coupled fluorescence assay of compound **II757** at 4 *K*_m_ substrate concentration ranging in concentrations from 0 to 100 µM and (**B**) concentration–response plot for compound **II757**. All experiments were performed in duplicates (n = 2).

Next, we examined the effect of the aromatic group on the PRMT1 inhibition. Compound **6f** containing a phenyl group displayed an IC_50_ of 0.29 ± 0.01 µM to PRMT1, resulting in about 2.5-fold reduction compared to **6d**. Surprisingly, increasing the length between a phenyl ring and guanidino group gave comparable inhibitory effects, as reflected by **6g** and **6i**. The aliphatic ring analogues **6d** and **6e** displayed about two- and sixfold better inhibitory activity than their respective aromatic analogues **6f** and **6g** (Table 1). Next, we introduced the bromo- or nitro substitution on the aromatic ring to evaluate the substituent effect. The selection was limited by readily available starting materials. Compound **6j** with a nitro group on the *para* position displayed an IC_50_ of 0.21 ± 0.01 µM, which was comparable to the parent analogue **6f**. The substitution of an *ortho*-Br on the phenyl ring generated **6k** (IC_50_ = 0.44 ± 0.03 µM), resulting in about a twofold loss of inhibitory activity. However, moving the bromide group to the *meta* and *par*a position led to **6l** and **6m**, respectively. According to the results (Table 1 and Figure 3), bromo-substitution on the *meta* position of the phenyl group produced the most potent inhibition. Interestingly, the *p*-Br on benzyl group displayed nearly threefold increased activity when we compared **6n** to **6g**, although the *p*-Br on phenyl ring produced comparable inhibitory activity against PRMT1 (Table 1). 

## 5. Inhibition Mechanism Studies

**Figure 4:**
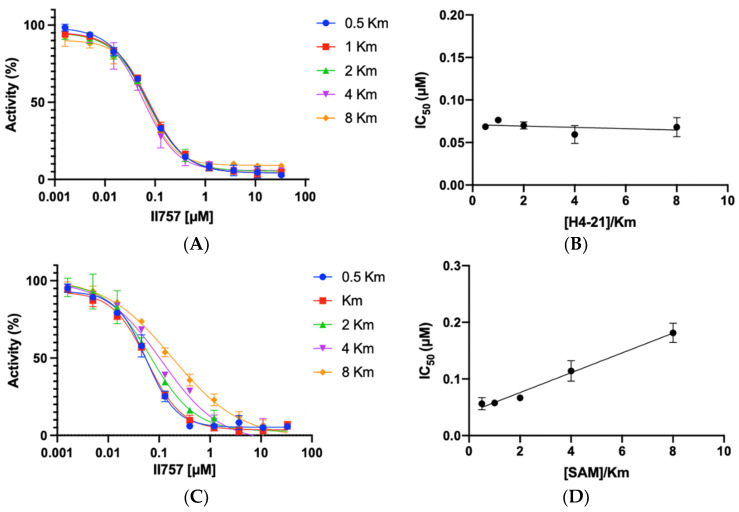
Inhibition mechanism studies of **II757**: (**A**) IC_50_ curves of **6l** at varying concentrations of substrate (H4-21) with a fixed concentration of SAM at 10 µM, (**B**) linear regression plot of IC_50_ values with corresponding concentrations of substrate (H4-21), (**C**) IC_50_ curves of **6l** at varying concentrations of SAM with a fixed concentration of substrate (H4-21) at 5 µM, and (**D**) linear regression plot of IC_50_ values with corresponding concentrations of SAM. All experiments were performed in duplicates (n = 2).

To understand the mode of action, the most potent inhibitor **6l** (**II757**) among this series was selected for an investigation of inhibition mechanism through a kinetic analysis using SAHH-coupled fluorescence assay with PRMT1. **II757** displayed a noncompetitive inhibition for the peptide substrate H4-21 as demonstrated by comparable IC_50_ values as the peptide concentration increased from 0.5 to 8 Km (Figure 4A,B). On the other hand, **II757** showed an explicit pattern of competitive inhibition for SAM, as demonstrated by a linear increase in the IC_50_ values proportional to the increased concentration of SAM (Figure 4C,D). This result indicated that **II757** act as a SAM-competitive inhibitor. Thus, its apparent *K*_i_ value is about 18 nM (Table 1 and Figure 3). Although the guanidino group did not reach the substrate-binding site, it may form additional interactions close to the SAM binding site to display improved potency. 

## 6. Selectivity Studies

We chose the top four potent compounds (**6b** and **6l–n**) to investigate their selectivities against several in-house methyltransferases including protein arginine methyltransferase *Tb*PRMT7, two members of the protein lysine methyltransferase PKMT (G9a and SETD7), protein N-terminal methyltransferase 1 (NTMT1), and a small molecule methyltransferase nicotinamide N-methyltransferase (NNMT), all of which have a SAM binding site. Additionally, SAHH was included as it is used in the coupled fluorescence assay, and it possesses a SAH binding site. 

**Figure 5:**
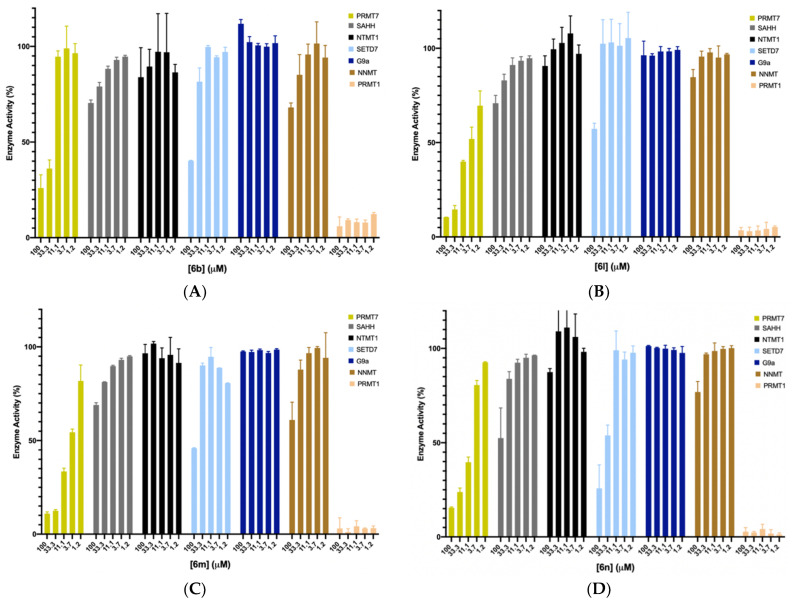
Selectivity studies for compounds **6b** (**A**) and **6l**–**n** (**B**–**D**) against *Tb*PRMT7, SAHH, NTMT1, SETD7, G9a, and NNMT with each compound at five different concentrations (100, 33, 11, 3.7, and 1.2 μM). All experiments were performed in duplicates (n = 2).

As shown in Figure 5, all four compounds did not show any significant inhibition to SAHH, NTMT1, SETD7, G9a, and NNMT at 33.3 μM. Except for **6l** (**II757**), three compounds (**6b**, **6m**, and **6n**) displayed over 50% inhibition to SETD7 at 100 μM. For *Tb*PRMT7, **6b** containing a cyclobutane group showed higher selectivity over PRMT7 as no inhibitory activity was observed against PRMT7 at 11.1 μM, confirming the possibility of structural modification of SAM analogue to achieve selectivity even among PRMTs. The most potent PRMT1 inhibitor **II757** displayed about 30% of the activity at 1.2 μM against PRMT7 while demonstrating slightly higher selectivity for other enzymes such as SETD7, G9a, NNMT, NTMT, and SAHH. 

## 7. Pan-PRMT Inhibition

**Figure 6:**
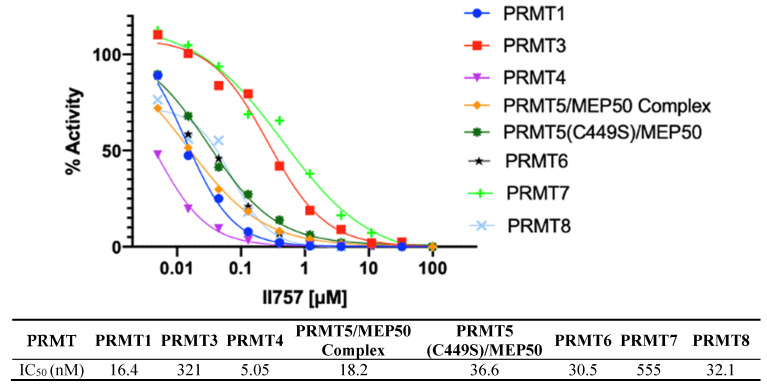
Selectivity of **II757** against the panel of PRMTs. **II757** was tested in 10 doses with threefold serial dilution in the HotSpot methyltransferase assay at reaction biology.

The most potent inhibitor **II757** was then sent to Reaction Biology to evaluate its selectivity against all the available PRMTs using a radioisotope ^3^H-AdoMet methyltransferase assay. As shown in Figure 6, **II757** inhibits all eight members of the PRMTs with an IC_50_ value ranging from 5.05 to 555 nM. It inhibits PRMT1 with an IC_50_ of 16 nM, which is similar to the value of 18 nM in our SAHH-coupled assay. Interestingly, **II757** demonstrated modest inhibition to PRMT3 and PRMT7 compared to other tested PRMT members and was most potent against PRMT4 with an IC_50_ of 5.05 nM. To our knowledge, this is the most potent SAM-competitive inhibitor for PRMT4 even compared to reported bisubstrate analogue **AH237**. Future studies will focus on obtaining structural information of **II757** bound to PRMTs to inform how to connect to a substrate portion to increase both potency and selectivity for each PRMT.

## 8. Molecular Docking

**Figure 7:**
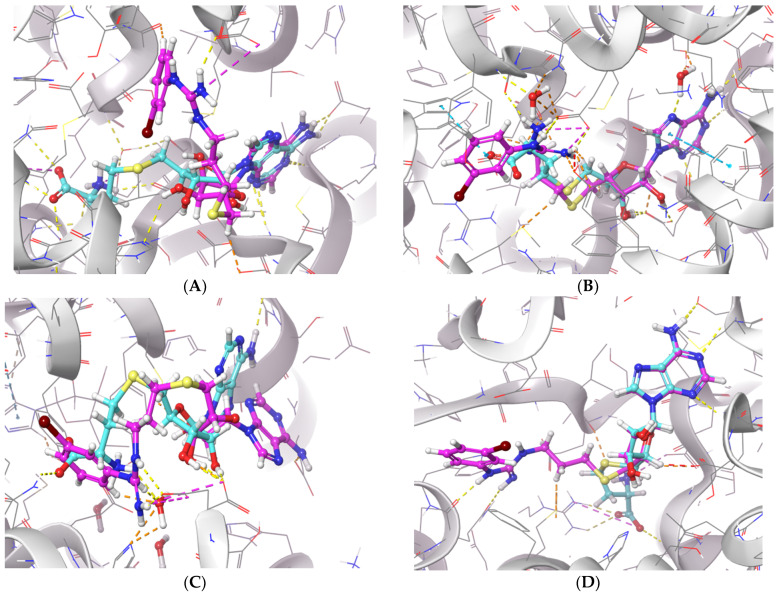
The *in silico* interaction of **II757** inside the pocket of (**A**) PRMT1 (PDB: 6nt2), (**B**) PRMT4 (PDB: 6s79), (**C**) PRMT5 (PDB: 6ckc), and (**D**) PRMT7 (PDB: 4m37). **II757** is shown in the magenta stick and ball model.

**Figure 8:**
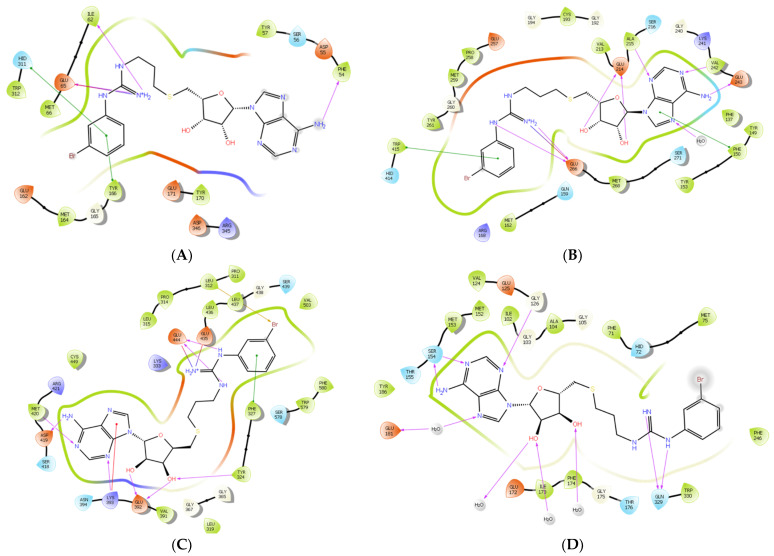
The predicted 2D interactions of **II757** inside the active site of (**A**) PRMT1 (pdb: 6nt2), (**B**) PRMT4 (pdb: 6s79), (**C**) PRMT5 (pdb: 6ckc), and (**D**) PRMT7 (pdb: 4m37). **II757** is shown in the black line model.

To further examine the binding mode of **II757**, we performed a molecular docking study on selected PRMT isoforms including PRMT1, 4, 5, and 7. The results indicated that **II757** virtually occupied the SAM binding site of the aforementioned PRMT isoforms, which is consistent with the SAM-competitive inhibition mechanism result (Figure 4). For PRMT 1/4/5, the *m*-bromophenyl substitution of **II757** formed a π–π interaction with multiple residues in the active site. Besides, multiple hydrogen bonding and van der Waals interactions were also observed. However, **II757** interacted with PRMT7 through several water molecule bridges and the *m*-bromophenyl substitution located at the edge of the binding pocket, possibly explaining the moderate inhibitory activity of **II757** for PRMT7 compared to other PRMTs (Figure 7 and Figure 8).

## 9. Inhibition on Cellular Methylation Level

**Figure 9:**
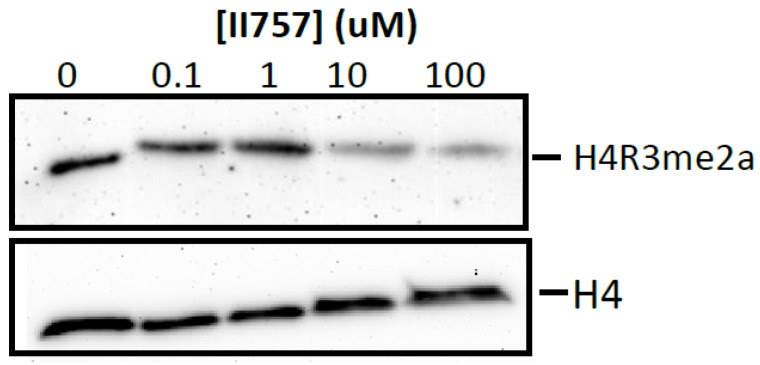
Cellular arginine methylation assay of **II757** in HEK293 cells. Representative Western blot results of effects of **II757** on histone H4 Arg3 asymmetric dimethylation (H4R3me2a) level at 0, 0.1, 1, 10, and 100 µM. H4 blot is shown as a loading control.

Furthermore, we proceeded to investigate the inhibitory effects of **II757** on the methylation level of histone H4 Arg3 via Western blotting to check its cellular inhibition. As shown in Figure 9, treatment with **II757** for 48 h in HEK293 cells resulted in a decrease in the asymmetric demethylation on Arg3 (H4R3me2a) level at 10 µM. Although cellular inhibition was about 20- to 1000-fold lower than the value obtained from the enzymatic assay, this preliminary study indicated that **II757** is cell potent. Future studies will focus on tethering this moiety with reported substrate-competitive inhibitors to enhance the selectivity and cellular potency. 

## 10. Conclusions

In summary, we have designed and synthesized a pan-PRMT inhibitor **II757** by incorporating an *m*-Br phenyl group on a guanidino group tethered to thioadenosine. This inhibitor showed inhibition against eight tested PRMT members with an IC_50_ value ranging from 5 to 555 nM. Furthermore, it showed the highest inhibitory activity against PRMT4 with an IC_50_ of 5 nM. Kinetic analysis revealed that **II757** is a SAM-competitive inhibitor for PRMT1, indicating that it can be tethered to a substrate-competitive inhibitor to further improve its inhibitory activity and selectivity for any specific member of the PRMT family. **II757** exhibited over 1000-fold selectivity over other methyltransferases such as SETD7, G9a, NTMT1, and NNMT. The *m*-Br phenyl group on the guanidino group has the potential to be derivatized by the formation of the sp^2^–sp^2^ bond through abundantly available chemical reactions. Compound **II757** can serve as a general probe in the screening for PRMT inhibitors and as a building block to construct cell-potent and selective PRMT inhibitors.

## 11. Experimental Section

Materials and instruments: The reagents and solvents were purchased from commercial sources (Fisher, Waltham, MA, USA and Sigma-Aldrich, St. Louis, MO, USA) and used directly. Analytical thin-layer chromatography was performed on ready-to-use plates with silica gel 60 (Merck, F254 Sigma-Aldrich, St. Louis, MO, USA). Flash column chromatography was performed over silica gel (grade 60, 230–400 mesh, Sigma-Aldrich, St. Louis, MO, USA) on the Teledyne ISCO CombiFlash purification system (Lincoln, NE, USA). Final compounds were purified on preparative reversed-phase high-pressure liquid chromatography (RP-HPLC) performed on Agilent 1260 Series system (Santa Clara, CA, USA). Systems were run with 0–50% methanol/water gradient with 0.1% TFA. NMR spectra were acquired on a Bruker AV500 instrument (Billerica, MA, USA) (500 MHz for ^1^H-NMR, 126 MHz for ^13^C-NMR). All the target compounds showed a purity of >95%.

### 11.1. General Procedure for the Synthesis of Thiourea

To a solution of amine in dichloromethane (3 mL), Fmoc-isothiocyanate (42 mg, 0.15 mmol, 1.5 eq.) was added at 0 °C. The reaction mixture was stirred at room temperature for 1–4 h. The volatiles were removed under reduced pressure and the crude was purified by column chromatography to afford **1a**–**1n**.

### 11.2. General Procedure for the Synthesis of Guanidine Moiety

To a solution of amine 4 (0.05 mmol, 1 eq.) in dichloromethane (2 mL), thiourea **1a**–**n** (0.75 mmol, 1.5 eq.), EDC (20 mg, 0.1 mmol, 2 eq.), and DIPEA (13 mg, 0.1 mmol, 2 eq.) were added. The reaction mixture was stirred overnight at room temperature. The mixture was washed with dichloromethane (3 × 2 mL) to produce **5a**–**n**. 

### 11.3. General Procedure for Final Compounds Synthesis

The mixture **5a**–**n** was treated with 20% piperidine in DCM for 10 min each. Then, it was concentrated and used for the next step. Further, the crude was dissolved in TFA/H_2_O (9;1). The reaction mixture was stirred at room temperature for 5 h. The mixture was evaporated by passing N_2_ gas and was then washed with dry ether. The residue was used for semi-preparatory HPLC separation using MeOH/H2O 10–40% to obtain final compounds **6a**–**n**. All NMR spectra, HRMS and HPLC analysis of compounds **6a**–**6n** are included in the supplementary materials.

1-(3-((((2S,3S,4R,5R)-5-(6-amino-9H-purin-9-yl)-3,4-dihydroxytetrahydrofuran-2-yl)methyl)thio)propyl)-3-ethylguanidine (**6a**). ^1^H NMR (500 MHz, Methanol-*d*_4_) δ 8.55 (s, 1H), 8.30 (s, 1H), 8.21 (s, 1H), 6.00 (d, *J* = 4.8 Hz, 1H), 4.80 (t, *J* = 5.1 Hz, 1H), 4.34 (t, *J* = 5.0 Hz, 1H), 4.24–4.17 (m, 1H), 3.25–3.16 (m, 4H), 3.03–2.90 (m, 2H), 2.62 (t, *J* = 7.1 Hz, 2H), 1.87–1.76 (m, 2H), 1.19 (t, *J* = 7.2 Hz, 3H). ^13^C NMR (126 MHz, MeOD) δ 170.33, 157.37, 157.33, 153.94, 150.66, 141.47, 120.58, 90.21, 85.72, 74.75, 74.10, 41.23, 37.43, 35.23, 30.60, 29.70, 14.43. HRMS *m/z* calc’d for C_16_H_26_N_8_O_3_S [M + H]^+^: 411.1921; found: 411.1923.

1-(3-((((2*S*,3*S*,4*R*,5*R*)-5-(6-amino-9*H*-purin-9-yl)-3,4-dihydroxytetrahydrofuran-2-yl)methyl)thio)propyl)-3-cyclobutylguanidine (**6b**). ^1^H NMR (500 MHz, Methanol-*d*_4_) δ 8.55 (s, 1H), 8.29 (s, 1H), 8.21 (s, 1H), 5.99 (d, *J* = 4.8 Hz, 1H), 4.80 (t, *J* = 5.1 Hz, 1H), 4.59 (s, 1H), 4.34 (t, *J* = 5.1 Hz, 1H), 4.27–4.17 (m, 1H), 4.02–3.89 (m, 1H), 3.22 (t, *J* = 6.9 Hz, 2H), 3.04–2.90 (m, 2H), 2.62 (t, *J* = 7.1 Hz, 2H), 2.44–2.30 (m, 2H), 2.08–1.93 (m, 2H), 1.86–1.70 (m, 4H). ^13^C NMR (126 MHz, MeOD) δ 157.39, 156.32, 153.95, 150.66, 141.49, 120.60, 90.28, 85.72, 74.72, 74.12, 47.59, 41.28, 35.24, 31.09, 30.58, 29.70, 15.72. HRMS *m/z* calc’d for C_18_H_28_N_8_O_3_S [M + H]^+^: 437.2078; found: 437.2079.

1-(3-((((2*S*,3*S*,4*R*,5*R*)-5-(6-amino-9*H*-purin-9-yl)-3,4-dihydroxytetrahydrofuran-2-yl)methyl)thio)propyl)-3-cyclopentylguanidine (**6c**). ^1^H NMR (500 MHz, Methanol-*d*_4_) δ 8.53 (s, 1H), 8.30 (s, 1H), 8.21 (s, 1H), 6.00 (d, *J* = 4.8 Hz, 1H), 4.80 (t, *J* = 5.1 Hz, 1H), 4.34 (t, *J* = 5.0 Hz, 1H), 4.23–4.17 (m, 1H), 3.87–3.77 (m, 1H), 3.24 (t, *J* = 6.9 Hz, 2H), 3.04–2.90 (m, 2H), 2.62 (t, *J* = 7.1 Hz, 2H), 2.03–1.93 (m, 2H), 1.86–1.78 (m, 2H), 1.77–1.68 (m, 2H), 1.67–1.59 (m, 2H), 1.55–1.45 (m, 2H). ^13^C NMR (126 MHz, MeOD) δ 157.38, 157.02, 153.95, 150.66, 141.47, 120.59, 90.24, 85.70, 74.74, 74.10, 54.32, 41.31, 35.24, 33.62, 30.60, 29.75, 24.44. HRMS *m/z* calc’d for C_19_H_30_N_8_O_3_S [M + H]^+^: 451.2234; found: 451.2236.

1-(3-((((2*S*,3*S*,4*R*,5*R*)-5-(6-amino-9*H*-purin-9-yl)-3,4-dihydroxytetrahydrofuran-2-yl)methyl)thio)propyl)-3-cyclohexylguanidine (**6d**). ^1^H NMR (500 MHz, Methanol-*d*_4_) δ 8.48 (s, 1H), 8.30 (s, 1H), 8.21 (s, 1H), 6.00 (d, *J* = 4.8 Hz, 1H), 4.80 (t, *J* = 5.1 Hz, 1H), 4.34 (t, *J* = 5.0 Hz, 1H), 4.24–4.17 (m, 1H), 3.38–3.32 (m, 1H), 3.23 (t, *J* = 6.9 Hz, 2H), 3.03–2.89 (m, 2H), 2.62 (t, *J* = 7.1 Hz, 2H), 1.93–1.86 (m, 2H), 1.82 (p, *J* = 7.0 Hz, 2H), 1.78–1.72 (m, 2H), 1.67–1.59 (m, 1H), 1.42–1.32 (m, 2H), 1.31–1.15 (m, 3H). ^13^C NMR (126 MHz, MeOD) δ 157.35, 156.52, 153.92, 150.65, 141.44, 120.57, 90.17, 85.66, 74.78, 74.07, 51.80, 41.24, 35.25, 33.77, 30.62, 29.72, 26.23, 25.71. HRMS *m/z* calc’d for C_20_H_32_N_8_O_3_S [M + H]^+^: 465.2391; found: 465.2392.

1-(3-((((2*S*,3*S*,4*R*,5*R*)-5-(6-amino-9*H*-purin-9-yl)-3,4-dihydroxytetrahydrofuran-2-yl)methyl)thio)propyl)-3-(cyclohexylmethyl)guanidine (**6e**). ^1^H NMR (500 MHz, Methanol-*d*_4_) δ 8.54 (s, 1H), 8.30 (s, 1H), 8.21 (s, 1H), 6.00 (d, *J* = 4.8 Hz, 1H), 4.80 (t, *J* = 5.1 Hz, 1H), 4.34 (t, *J* = 5.0 Hz, 1H), 4.24–4.16 (m, 1H), 3.23 (t, *J* = 6.9 Hz, 2H), 3.04–2.91 (m, 4H), 2.63 (t, *J* = 7.1 Hz, 2H), 1.88–1.79 (m, 2H), 1.78–1.72 (m, 4H), 1.71–1.64 (m, 1H), 1.58–1.47 (m, 1H), 1.33–1.14 (m, 3H), 1.01–0.89 (m, 2H). ^13^C NMR (126 MHz, MeOD) δ 157.60, 157.37, 153.94, 150.65, 141.46, 120.58, 90.22, 85.68, 74.75, 74.09, 41.26, 38.68, 35.25, 31.61, 30.60, 29.71, 27.38, 26.81. HRMS *m/z* calc’d for C_21_H_34_N_8_O_3_S [M + H]^+^: 479.2547; found: 479.2548.

1-(3-((((2*S*,3*S*,4*R*,5*R*)-5-(6-amino-9*H*-purin-9-yl)-3,4-dihydroxytetrahydrofuran-2-yl)methyl)thio)propyl)-3-phenylguanidine (**6f**). ^1^H NMR (500 MHz, Methanol-*d*_4_) δ 8.47 (s, 1H), 8.37 (s, 1H), 7.43–7.34 (m, 2H), 7.33–7.27 (m, 3H), 6.05 (d, *J* = 4.9 Hz, 1H), 4.73 (t, *J* = 5.1 Hz, 1H), 4.41 (s, 2H), 4.32 (t, *J* = 5.0 Hz, 1H), 4.25–4.17 (m, 1H), 3.03–2.87 (m, 2H), 2.59 (t, *J* = 7.1 Hz, 2H), 1.90–1.80 (m, 2H). ^13^C NMR (126 MHz, MeOD) δ 156.94, 152.71, 150.10, 146.49, 143.72, 136.40, 131.07, 128.52, 126.45, 120.62, 90.50, 85.78, 75.28, 74.01, 41.62, 35.28, 30.59, 29.61. HRMS *m/z* calc’d for C_20_H_26_N_8_O_3_S [M + H]^+^: 459.1921; found: 459.1921.

1-(3-((((2*S*,3*S*,4*R*,5*R*)-5-(6-amino-9*H*-purin-9-yl)-3,4-dihydroxytetrahydrofuran-2-yl)methyl)thio)propyl)-3-benzylguanidine (**6g**). ^1^H NMR (500 MHz, Methanol-*d*_4_) δ 8.47 (s, 1H), 8.37 (s, 1H), 7.40–7.35 (m, 2H), 7.33–7.26 (m, 3H), 6.05 (d, *J* = 4.9 Hz, 1H), 4.73 (t, *J* = 5.1 Hz, 1H), 4.41 (s, 2H), 4.32 (t, *J* = 5.0 Hz, 1H), 4.24–4.18 (m, 1H), 3.29 (d, *J* = 6.9 Hz, 2H), 3.02–2.86 (m, 2H), 2.59 (t, *J* = 7.1 Hz, 2H), 1.89–1.79 (m, 2H). ^13^C NMR (126 MHz, MeOD) δ 157.57, 153.32, 150.22, 147.64, 143.40, 137.74, 129.93, 129.01, 128.21, 90.45, 85.71, 75.19, 74.02, 45.91, 41.36, 35.27, 30.47, 29.65. HRMS *m/z* calc’d for C_21_H_28_N_8_O_3_S [M + H]^+^: 473.2078; found: 473.2079.

1-(3-((((2*S*,3*S*,4*R*,5*R*)-5-(6-amino-9*H*-purin-9-yl)-3,4-dihydroxytetrahydrofuran-2-yl)methyl)thio)propyl)-3-phenethylguanidine (**6h**). ^1^H NMR (500 MHz, Methanol-*d*_4_) δ 8.36 (s, 1H), 8.30 (s, 1H), 8.20 (s, 1H), 7.32–7.26 (m, 2H), 7.25–7.17 (m, 3H), 6.00 (d, *J* = 4.8 Hz, 1H), 4.80 (t, *J* = 5.1 Hz, 1H), 4.34 (t, *J* = 5.0 Hz, 1H), 4.24–4.17 (m, 1H), 3.43 (t, *J* = 7.1 Hz, 2H), 3.17 (t, *J* = 6.9 Hz, 2H), 3.02–2.89 (m, 2H), 2.85 (t, *J* = 7.1 Hz, 2H), 2.58 (t, *J* = 7.1 Hz, 2H), 1.81–1.70 (m, 2H). ^13^C NMR (126 MHz, MeOD) δ 168.06, 157.43, 153.90, 150.64, 141.46, 139.38, 129.90, 129.68, 127.78, 120.57, 90.20, 85.68, 74.76, 74.08, 43.76, 41.20, 36.01, 35.23, 30.58, 29.59. HRMS *m/z* calc’d for C_22_H_30_N_8_O_3_S [M + H]^+^: 487.2234; found: 487.2235.

1-(3-((((2*S*,3*S*,4*R*,5*R*)-5-(6-amino-9*H*-purin-9-yl)-3,4-dihydroxytetrahydrofuran-2-yl)methyl)thio)propyl)-3-(3-phenylpropyl)guanidine (**6i**). ^1^H NMR (500 MHz, Methanol-*d*_4_) δ 8.29 (s, 1H), 8.20 (s, 1H), 7.25 (t, *J* = 7.6 Hz, 2H), 7.19–7.14 (m, 3H), 6.00 (d, *J* = 4.9 Hz, 1H), 4.80 (t, *J* = 5.1 Hz, 1H), 4.34 (t, *J* = 5.0 Hz, 1H), 4.25–4.18 (m, 1H), 3.24–3.12 (m, 4H), 3.02–2.90 (m, 2H), 2.68–2.58 (m, 4H), 1.94–1.85 (m, 2H), 1.84–1.77 (m, 2H). ^13^C NMR (126 MHz, MeOD) δ 157.45, 157.32, 153.92, 150.62, 142.30, 141.39, 129.50, 129.38, 127.11, 120.55, 90.13, 85.65, 74.78, 74.06, 42.00, 41.25, 35.25, 33.68, 31.55, 30.60, 29.66. HRMS *m/z* calc’d for C_23_H_32_N_8_O_3_S [M + H]^+^: 501.2391; found: 501.2394.

1-(3-((((2*S*,3*S*,4*R*,5*R*)-5-(6-amino-9*H*-purin-9-yl)-3,4-dihydroxytetrahydrofuran-2-yl)methyl)thio)propyl)-3-(2-nitrophenyl)guanidine (**6j**). ^1^H NMR (500 MHz, Methanol-*d*_4_) δ 8.56 (s, 1H), 8.30 (s, 1H), 8.21 (s, 1H), 8.11 (dd, *J* = 8.2, 1.5 Hz, 1H), 7.75 (td, *J* = 7.8, 1.5 Hz, 1H), 7.56–7.47 (m, 2H), 6.00 (d, *J* = 4.8 Hz, 1H), 4.35 (t, *J* = 5.1 Hz, 1H), 4.26–4.17 (m, 1H), 3.06–2.91 (m, 2H), 2.70–2.62 (m, 2H), 1.92–1.83 (m, 2H). ^13^C NMR (126 MHz, MeOD) δ 157.20, 156.61, 153.78, 150.48, 141.30, 138.16, 132.30, 130.99, 128.83, 124.61, 123.86, 120.43, 90.09, 85.55, 74.58, 73.94, 41.55, 35.09, 30.49, 29.29. HRMS *m/z* calc’d for C_20_H_25_N_9_O_3_S [M + H]^+^: 504.1772; found: 504.1774.

1-(3-((((2*S*,3*S*,4*R*,5*R*)-5-(6-amino-9*H*-purin-9-yl)-3,4-dihydroxytetrahydrofuran-2-yl)methyl)thio)propyl)-3-(2-bromophenyl)guanidine (**6k**). ^1^H NMR (500 MHz, Methanol-*d*_4_) δ 8.55 (s, 1H), 8.30 (s, 1H), 8.21 (s, 1H), 7.75 (dd, *J* = 8.1, 1.4 Hz, 1H), 7.49–7.44 (m, 1H), 7.39 (dd, *J* = 7.9, 1.7 Hz, 1H), 7.36–7.30 (m, 1H), 6.00 (d, *J* = 4.8 Hz, 1H), 4.59 (s, 2H), 4.34 (t, *J* = 5.1 Hz, 1H), 4.24–4.17 (m, 1H), 3.04–2.91 (m, 2H), 2.65 (t, *J* = 7.1 Hz, 2H), 1.92–1.83 (m, 2H). ^13^C NMR (126 MHz, MeOD) δ 157.71, 157.12, 154.29, 150.99, 141.81, 138.67, 132.81, 131.50, 129.34, 125.12, 124.37, 120.94, 90.60, 86.06, 75.09, 74.45, 42.06, 35.60, 31.00, 29.80. HRMS *m/z* calc’d for C_20_H_25_BrN_8_O_3_S [M + H]^+^: 537.1026; found: 537.1026. 

1-(3-((((2*S*,3*S*,4*R*,5*R*)-5-(6-amino-9*H*-purin-9-yl)-3,4-dihydroxytetrahydrofuran-2-yl)methyl)thio)propyl)-3-(3-bromophenyl)guanidine (**6l**). ^1^H NMR (500 MHz, Methanol-*d*_4_) δ 8.51 (s, 1H), 8.30 (s, 1H), 8.21 (s, 1H), 7.54–7.43 (m, 2H), 7.35 (t, *J* = 8.0 Hz, 1H), 7.22 (dd, *J* = 8.4, 1.8 Hz, 1H), 6.00 (d, *J* = 4.8 Hz, 1H), 4.80 (t, *J* = 5.1 Hz, 1H), 4.35 (t, *J* = 5.1 Hz, 1H), 4.28–4.17 (m, 1H), 3.33 (d, *J* = 6.9 Hz, 2H), 3.09–2.91 (m, 2H), 2.66 (t, *J* = 7.1 Hz, 2H), 1.96–1.81 (m, 2H). ^13^C NMR (126 MHz, MeOD) δ 157.37, 156.78, 153.95, 150.65, 141.47, 138.33, 132.47, 131.16, 129.00, 124.78, 124.03, 120.60, 90.26, 85.72, 74.75, 74.11, 41.72, 35.26, 30.66, 29.46. HRMS *m/z* calc’d for C_20_H_25_BrN_8_O_3_S [M + H]^+^: 537.1026; found: 537.1027.

1-(3-((((2*S*,3*S*,4*R*,5*R*)-5-(6-amino-9*H*-purin-9-yl)-3,4-dihydroxytetrahydrofuran-2-yl)methyl)thio)propyl)-3-(4-bromophenyl)guanidine (**6m**). ^1^H NMR (500 MHz, Methanol-*d*_4_) δ 8.34 (s, 1H), 8.30 (s, 1H), 8.21 (s, 1H), 7.62–7.55 (m, 2H), 7.16 (d, *J* = 8.3 Hz, 2H), 6.00 (d, *J* = 4.8 Hz, 1H), 4.80 (t, *J* = 5.1 Hz, 1H), 4.34 (t, *J* = 5.1 Hz, 1H), 4.26–4.16 (m, 1H), 3.33 (s, 2H), 3.05–2.91 (m, 2H), 2.65 (t, *J* = 7.1 Hz, 2H), 1.93–1.81 (m, 2H). ^13^C NMR (126 MHz, MeOD) δ 157.37, 156.81, 153.94, 150.66, 141.48, 135.91, 134.11, 128.09, 121.53, 120.60, 90.25, 85.73, 74.75, 74.11, 41.67, 35.25, 30.63, 29.51. HRMS *m/z* calc’d for C_20_H_25_BrN_8_O_3_S [M + H]^+^: 537.1026; found: 537.1028.

1-(3-((((2*S*,3*S*,4*R*,5*R*)-5-(6-amino-9*H*-purin-9-yl)-3,4-dihydroxytetrahydrofuran-2-yl)methyl)thio)propyl)-3-(4-bromobenzyl)guanidine (**6n**). ^1^H NMR (500 MHz, Methanol-*d*_4_) δ 8.61–8.48 (m, 1H), 8.29 (s, 1H), 8.21 (s, 1H), 7.56–7.49 (m, 2H), 7.27–7.20 (m, 2H), 6.00 (d, *J* = 4.9 Hz, 1H), 4.80 (t, *J* = 5.1 Hz, 1H), 4.63 (d, *J* = 26.9 Hz, 2H), 4.37 (s, 2H), 4.34 (t, *J* = 5.1 Hz, 1H), 4.23–4.17 (m, 1H), 3.26 (t, *J* = 6.8 Hz, 2H), 3.01–2.89 (m, 2H), 2.58 (t, *J* = 7.1 Hz, 2H), 1.85–1.76 (m, 2H). ^13^C NMR (126 MHz, MeOD) δ 157.55, 157.37, 153.95, 150.65, 141.46, 137.13, 132.99, 130.09, 122.68, 120.59, 90.23, 85.67, 74.74, 74.10, 45.23, 41.37, 35.25, 30.54, 29.59. HRMS *m/z* calc’d for C_21_H_27_BrN_8_O_3_S [M + H]^+^: 551.1183; found: 551.1185.

### 11.4. PRMT1 Biochemical Assays and Enzyme Kinetics Study

A fluorescence-based SAHH-coupled assay was used to evaluate the IC50 values of inhibitors by monitoring the production of SAH from SAM after methylation. The condition of the assay in a final well volume of 100 μL was: 2.5 mM HEPES (pH = 7.0), 25 mM NaCl, 25 μM EDTA, 50 μM TCEP, 0.01% Triton X-100, 5 μM SAHH, 0.1 μM PRMT1, 10 μM AdoMet, and 10 μM ThioGlo4. After incubating with the inhibitors for 10 min at 37 °C, reactions were initiated by the addition of 5 μM H4-21 peptide (Km value). The fluorescence signal was monitored on a BMG CLARIOstar microplate reader with excitation at 400 nm and emission at 465 nm for 18 min. Data were processed using GraphPad Prism software 7.0. All experiments were performed in duplicate. 

Where the IC_50_ at *K*_m_ value was less than the enzyme concentration, the condition of the assay was modified to a final well volume of 100 μL: 2.5 mM HEPES (pH = 7.0), 25 mM NaCl, 25 μM EDTA, 50 μM TCEP, 0.01% Triton X-100, 5 μM SAHH, 0.1 μM PRMT1, 10 μM AdoMet, and 10 μM ThioGlo4. After incubating with the inhibitors for 10 min at 37 °C, reactions were initiated by the addition of 20 μM H4-21 peptide (4 *K*_m_ value). The fluorescence signal was monitored on a BMG CLARIOstar microplate reader with excitation at 400 nm and emission at 465 nm for 18 min. Data were processed using GraphPad Prism software 7.0. All experiments were performed in duplicate. Data were processed using GraphPad Prism software 7.0. *K*_i, app_ was calculated using the equation *K*_i, app_ = IC_50_/(1 + [S]/*K*_m_).

### 11.5. Selectivity Assays

A fluorescence-based SAHH-coupled assay was applied to study the effect of the compound on methyltransferase activity of PRMT7, NTMT1, SETD7, G9a, NNMT, and SAHH. For PRMT7, the assay was performed in a final well volume of 100 μL: 25 mM Tris (pH = 7.5), 50 mM KCl, 0.01% Triton X-100, 5 μM SAHH, 0.2 μM PRMT7, 3 μM AdoMet, and 15 μM ThioGlo1. After incubation for 10 min with the inhibitor, reactions were initiated by the addition of 60 μM H4-21 peptide, and the reaction was monitored for 15 min. For NTMT1, the assay was performed in a final well volume of 100 μL: 25 mM Tris (pH = 7.5), 50 mM KCl, 0.01% Triton X-100, 5 μM SAHH, 0.1 μM NTMT1, 3 μM AdoMet, and 10 μM ThioGlo4. After incubation for 10 min with the inhibitor, reactions were initiated by the addition of 0.5 μM GPKRIA peptide, and the reaction was monitored for 15 min. For SETD7, the assay was performed in a final well volume of 100 μL: 25 mM potassium phosphate buffer (pH = 7.6), 0.01% Triton X-100, 5 μM SAHH, 1 μM SETD7, 2 μM AdoMet, and 10 μM ThioGlo1. After incubation for 10 min with the inhibitor, reactions were initiated by the addition of 90 μM H3-21 peptide, and the reaction was monitored for 15 min. For G9a, the assay was performed in a final well volume of 100 μL: 25 mM potassium phosphate buffer (pH = 7.6), 1 mM EDTA, 2 mM MgCl_2_, 0.01% Triton X-100, 5 μM SAHH, 0.1 μM His-G9a, 10 μM AdoMet, and 10 μM ThioGlo4. For NNMT, the assay was performed in a final well volume of 100 μL: 25 mM Tris (pH = 7.5), 50 mM KCl, 0.01% Triton X- 100, 5 μM SAHH, 0.1 μM NNMT, 10 μM AdoMet, and 10 μM ThioGlo1. After incubation for 10 min with the inhibitor, reactions were initiated by the addition of 10 μM nicotinamide, and the reaction was monitored for 18 min. The inhibitors were added at five concentrations: 100, 33.3, 11.1, 3.7, and 1.2 μM. 

The effect of the inhibitors on the coupled enzyme, SAHH, activity was also evaluated. The assay was performed in a final well volume of 100 μL: 25 mM Tris (pH = 7.5), 50 mM KCl, 0.01% Triton X-100, 0.1 μM SAHH, and 15 μM ThioGlo1. After incubation for 10 min with the compound, 0.5 μM SAH was added to initiate the reactions. All experiments were performed in duplicate. Fluorescence was monitored on a BMG CLARIOstar microplate reader with excitation at 380 nm and emission at 505 nm.

### 11.6. Inhibition Mechanism Studies

The fluorescence-based SAHH-coupled assay was employed for the enzyme kinetic study. Varying concentrations of SAM (from 0.5 to 8 Km) with 5 μM fixed concentration of H4-21 or varying concentration of H4-21 (from 0.5 to 8 Km) with 10 μM fixed concentration of SAM was included in reactions with a series concentration of compounds. All the IC50 values were determined in triplicate. Fluorescence was monitored on a BMG CLARIOstar microplate with excitation at 380 nm and emission at 505 nm. Data were processed using GraphPad Prism software 7.0.

### 11.7. Molecular Docking

To evaluate the binding of **II757** on selected PRMT isoforms, a Glide software of Schrödinger Maestro 2021-1 release was implicated. The crystal structures of PRMT1, PRMT4, PRMT5, and PRMT7 were downloaded from protein data bank (www.rcsb.org) with PDB code of 6nt2, 6s79, 6ckc, and 4m37 (accessed on 26 April 2021), respectively. The proteins were prepared by minimizing energy with OPLS_2005 force field. LigPrep was used to sketch the chemical structure of **II757** as the ionization state was generated at pH 7.0 ± 2.0. Glide is applied to generate grids for PRMT subtypes using the default parameters without any protein constraint. A cubic box of specific dimensions centered around the active site residues was generated for the proteins. The bounding box was set to 12 Å × 12 Å × 12 Å. Flexible docking was performed into the prepared proteins using extra precision mode by keeping all docking parameters as default. OPLS_2005 force field was used to calculate the binding free energy, and 10 poses per docked ligand were saved. 

### 11.8. Cellular Methylation Level

HEK293 cell line was cultured in DMEM media supplemented with 10% FBS and 1% antibiotic-antimycotic (Gibco, Fisher, Waltham, MA, USA). The cells were maintained in a tissue culture dish (Falcon 353003, Fisher, Waltham, MA, USA) until seeding at a density of 0.03 × 10^6^ cells/mL into a 24-well tissue culture plate (Falcon 353047, Fisher, Waltham, MA, USA). The cells were incubated overnight at 37 °C in 5% CO_2_ with the lid on. They were then treated with the inhibitors at different concentrations for 48 h. The media was removed, the cells were washed three times with cold 1X PBS, and lysed on ice in cold SDS-containing buffer (25 mM Tris-HCl pH 7.6, 150 mM NaCl, 1% NP-40, 1% sodium deoxycholate, 0.1% SDS, and Halt protease and phosphate inhibitor, 1861280) for 10 min. The cell lysate was sonicated and centrifuged at 14,000× *g* for 10 min to collect the debris.

The protein was quantified using BCA (Pierce BCA Protein Assay Kit, 23225, Fisher, Waltham, MA, USA) and equal amounts of protein were resolved by SDS-PAGE electrophoresis and blotted on Transfer membrane (Immun-Blot PVDF Membranes, Bio-Rad, Hercules, CA, USA Bio-Rad, 1620177). The membrane was blocked in blocking buffer (5% milk in 1X TBST) for 1 h at RT followed by overnight incubation with primary antibodies (Histone H4R3me2a, ABclonal, Woburn, MA, USA, ABclonal, A2376, and Histone H4, ABclonal, A1131) at 4 °C. After washing three times with 1X TBST for 5 min each, the membrane was incubated with HRP-linked secondary antibodies (Cell Signaling Technologies, Danvers, MA, USA. Cell Signaling Technology, 7074S) at RT for 1 h. The membranes were washed three times with 1X TBST for 5 min each, and the proteins were detected by ECL (Bio-Rad, Hercules, CA, USA).

## Figures and Tables

**Table 1:** SAR of synthesized compounds **6a–6n**.

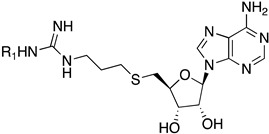			
Compound	R_1_	IC_50_ (µM) *	*K*_i, app_ (µM) ^b^
**6a**	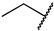	0.62 ± 0.08	0.31 ± 0.04
**6b**	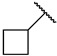	0.23 ± 0.02 ^a^	0.045 ± 0.005
**6c**	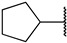	0.22 ± 0.016	0.11 ± 0.008
**6d**	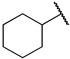	0.11 ± 0.048	0.055 ± 0.024
**6e**	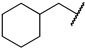	0.12 ± 0.026	0.06 ± 0.013
**6f**	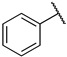	0.29 ± 0.01	0.15 ± 0.005
**6g**	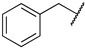	0.72 ± 0.15	0.36 ± 0.075
**6h**	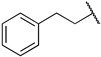	0.21 ± 0.06	0.11 ± 0.03
**6i**	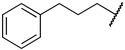	0.33 ±0.11	0.17 ± 0.055
**6j**	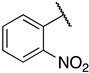	0.21 ± 0.01	0.11 ± 0.005
**6k**	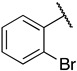	0.44 ± 0.03	0.22 ± 0.015
**6l (II757)**	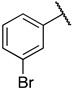	0.09 ± 0.04 ^a^	0.018 ± 0.008
**6m**	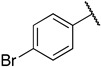	0.29 ± 0.04 ^a^	0.058 ± 0.007
**6n**	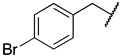	0.26 ± 0.07 ^a^	0.052 ± 0.014

^a^ These experiments were performed with substrate concentration at 4 *K*_m_ as the IC_50_ at *K*_m_ value was less than the enzyme concentration. ^b^ *K*_i, app_ was calculated using the equation *K*_i, app_ = IC_50_/(1 + [S]/*K*_m_). * IC_50_ values were performed in duplicates (n = 2) and presented as mean ± SD.

## Data Availability

Not applicable.

## Supplementary Materials

The following are available online at https://www.mdpi.com/article/10.3390/biom11060854/s1, NMR spectra, HRMS and HPLC analysis of compounds **6a**–**n**.
