# Supplementary material for: A Pan-Inhibitor for Protein Arginine Methyltransferase Family Enzymes
Source: Biomolecules. 2021 Jun 8;11(6):854. doi: 10.3390/biom11060854 (PMC8230315; doi:10.3390/biom11060854)

Supplementary Materials

A Pan-Inhibitor for Protein Arginine Methyltransferase Family Enzymes

Iredia D. Iyamu[1], Ayad A. Al-Hamashi[1,2] and Rong Huang[1,*]

[1] Department of Medicinal Chemistry and Molecular Pharmacology, Center for Cancer Research, Institute for Drug Discovery, Purdue University, West Lafayette, Indiana 47907, United States
[2] Department of Pharmaceutical Chemistry, College of Pharmacy, University of Baghdad, Bab-almoadham, Baghdad, 10047, Iraq
* Correspondence: huang-r@purdue.edu Tel.: +001 765 494 3426

NMR spectra, HRMS and HPLC analysis of compounds 6a-6n

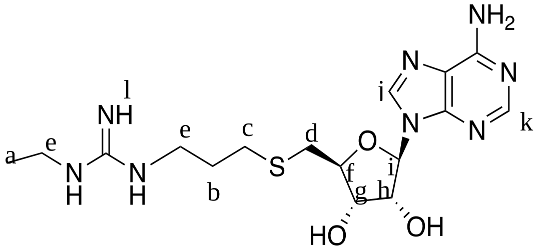
NH2
N
N
N
N
i
k
l
NH
a
e
N
H
N
H
e
c
b
S
d
O
f
g
h
i
HO
OH

6a

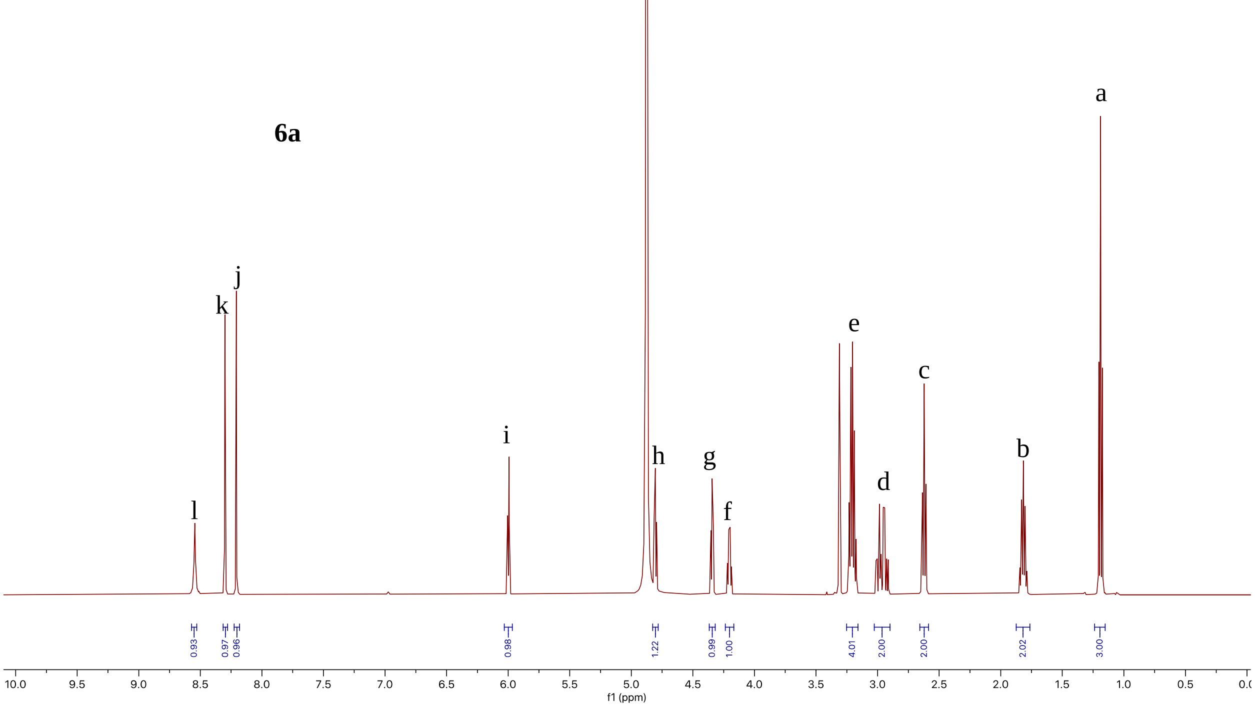
l
k
j
i
h
g
f
e
d
c
b
a
0.93
0.97
0.96
0.98
1.22
0.99
1.00
4.01
2.00
2.00
2.02
3.00
10.0 9.5 9.0 8.5 8.0 7.5 7.0 6.5 6.0 5.5 5.0 4.5 4.0 3.5 3.0 2.5 2.0 1.5 1.0 0.5
f1 (ppm)

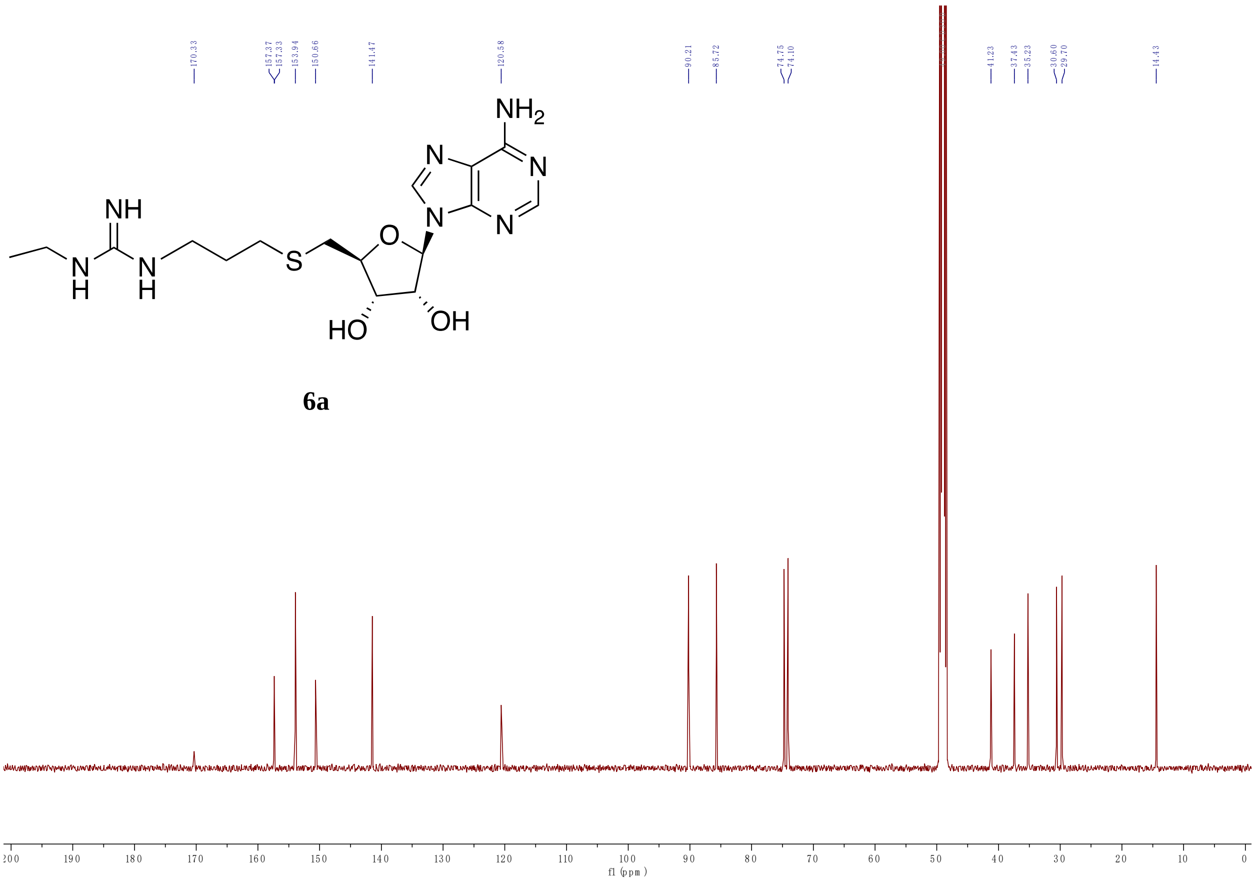

HRMS Spectra of 6a

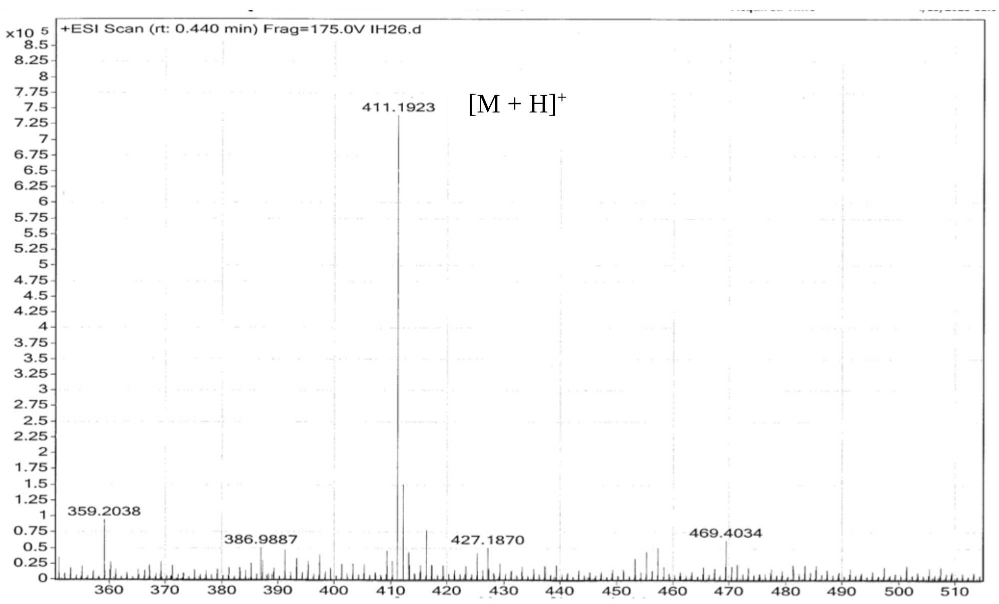

HPLC Spectra of 6a

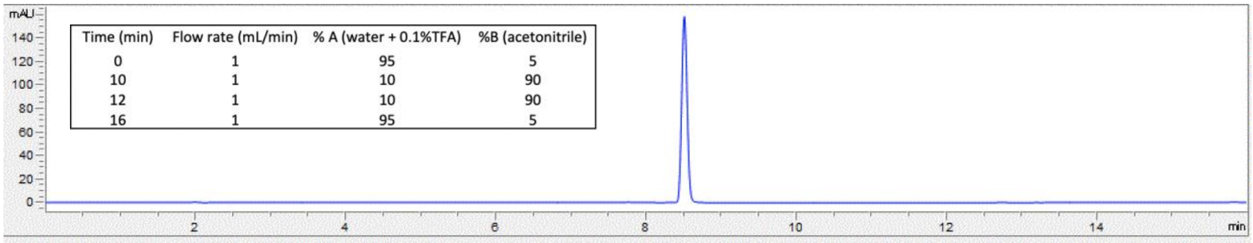

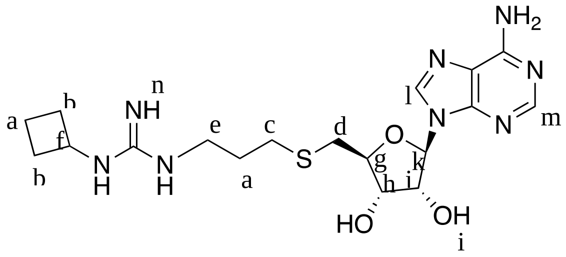
NH2
N
N
N
N
NH
N
H
N
H
S
O
HO
OH
a
b
f
n
e
c
d
g
h
i
k
l
m

6b

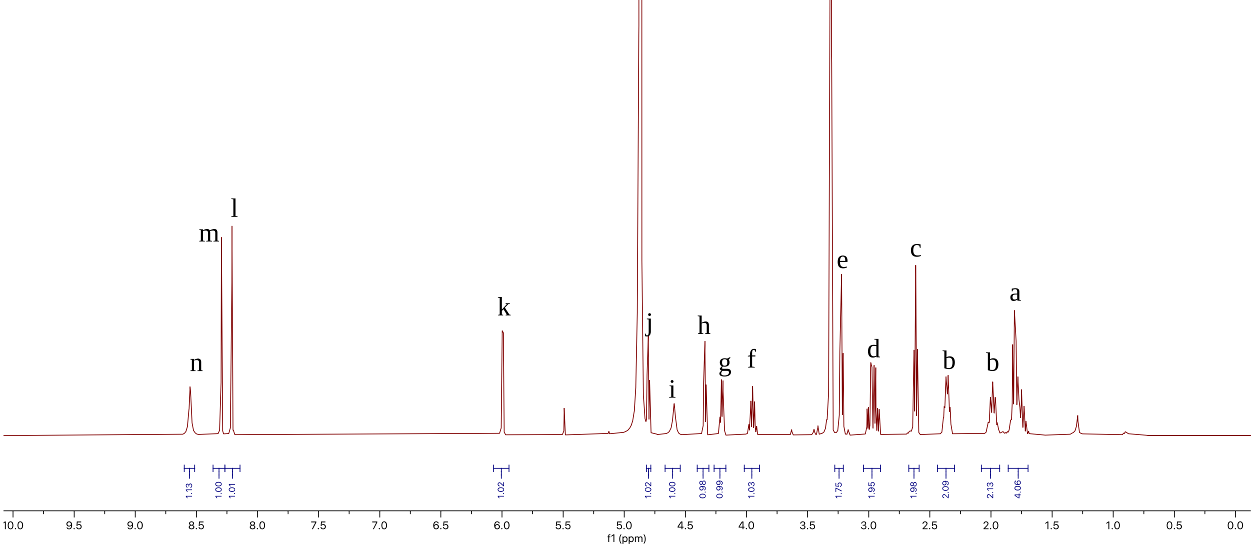
n
m
l
k
j
i
h
g
f
e
d
c
b
b
a
1.13
1.00
1.01
1.02
1.02
1.00
0.98
0.99
1.03
1.75
1.95
1.98
2.09
2.13
4.06
10.0 9.5 9.0 8.5 8.0 7.5 7.0 6.5 6.0 5.5 5.0 4.5 4.0 3.5 3.0 2.5 2.0 1.5 1.0 0.5 0.0
f1 (ppm)

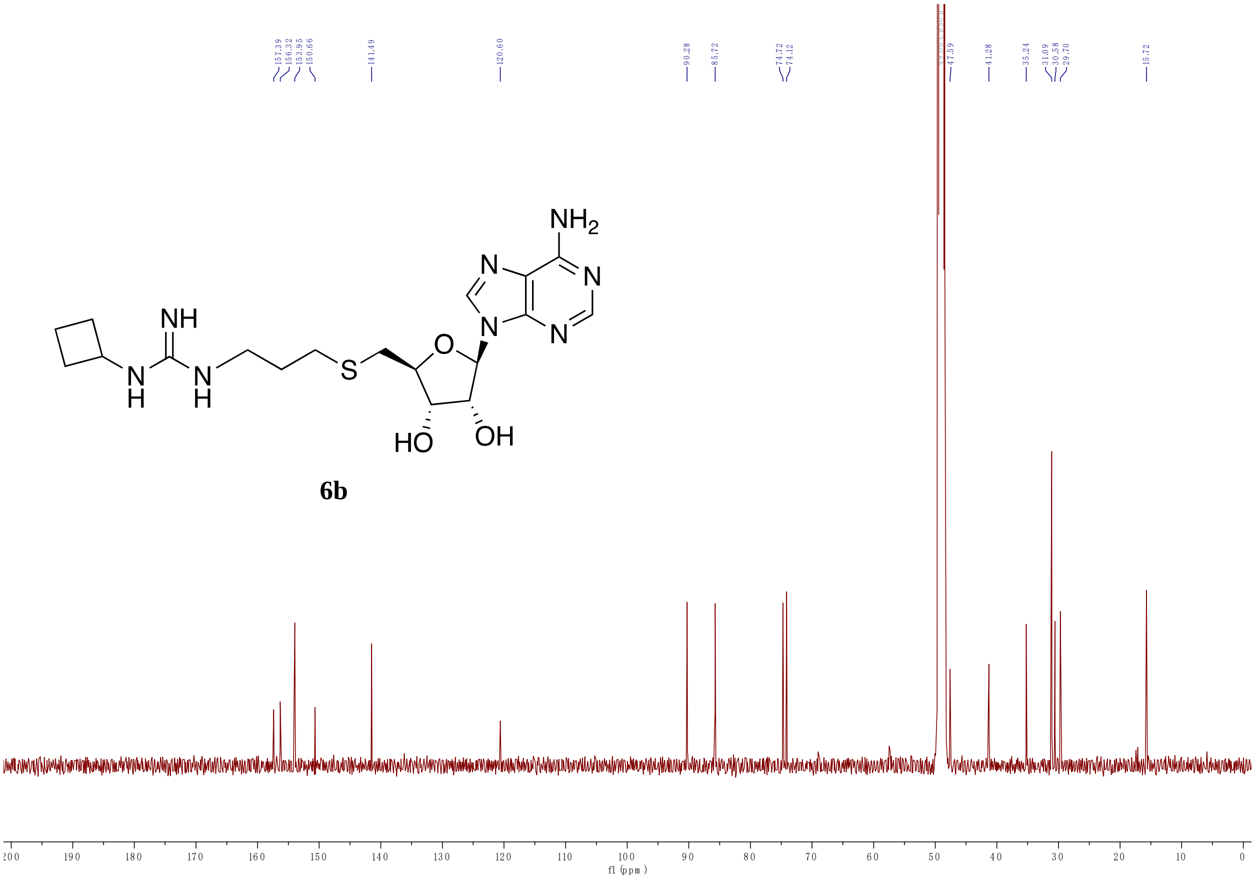

HRMS Spectra of 6b

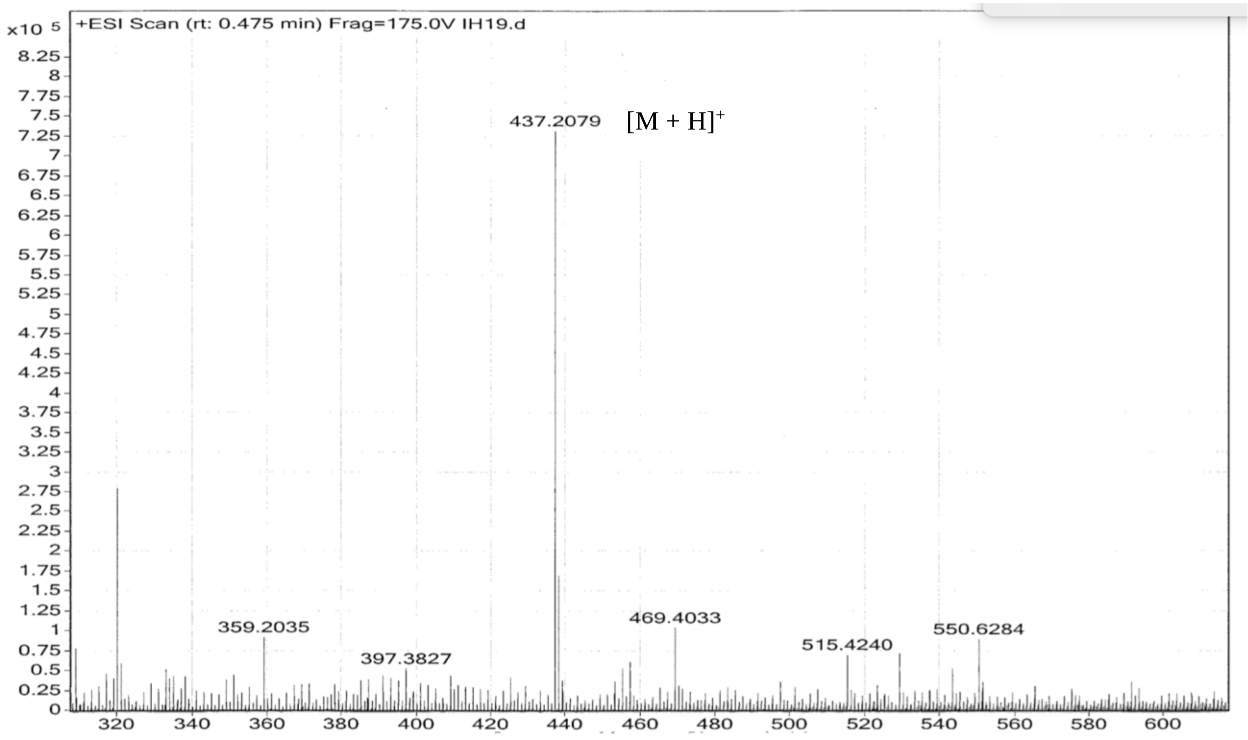

HPLC Spectra of 6b

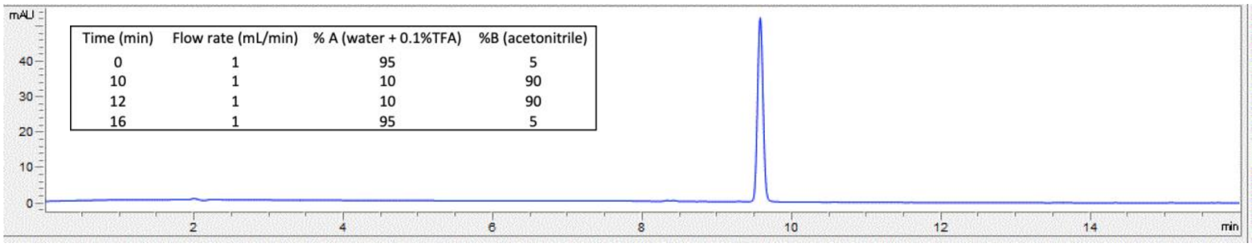

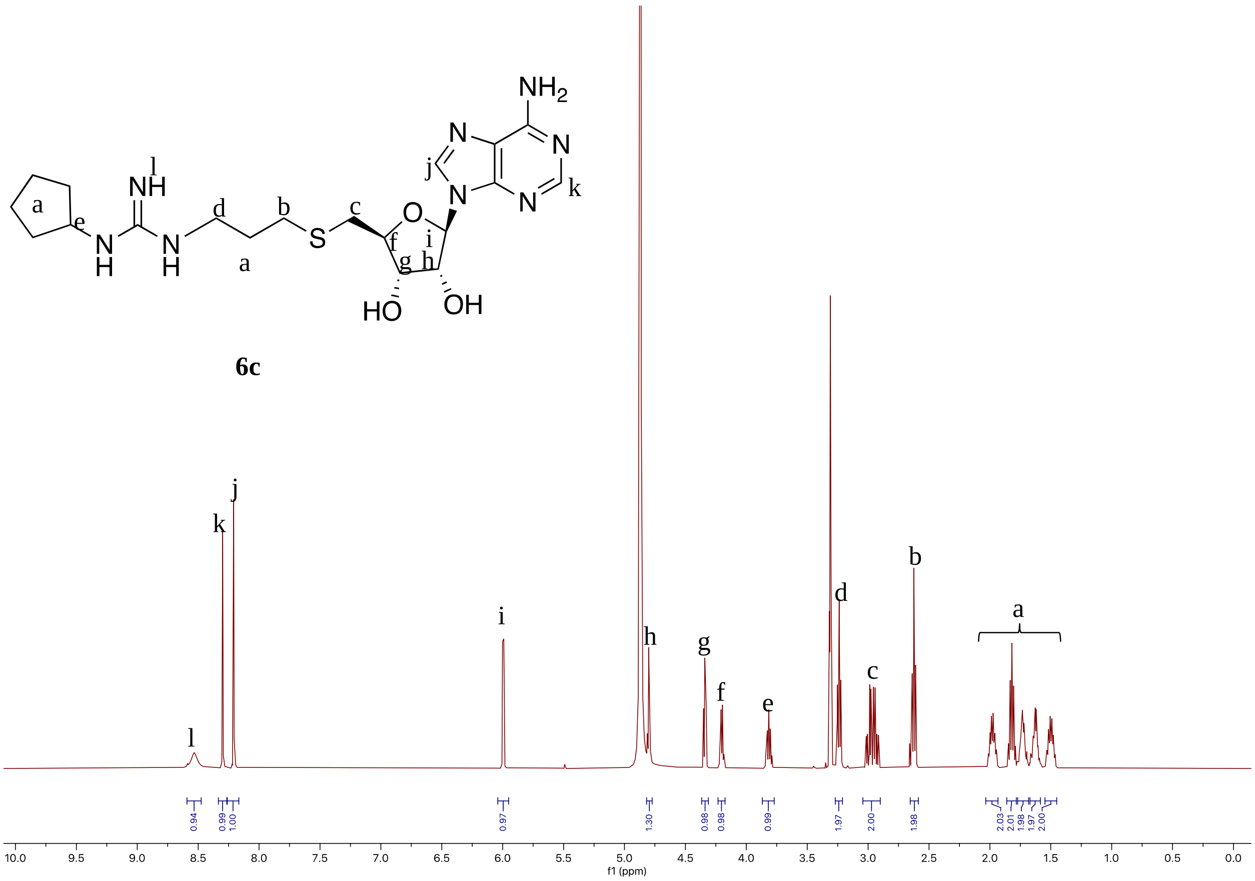
NH2
N
N
j
N
N
k
l
NH
a
e
N
H
N
H
d
a
b
S
c
O
f
i
g
h
HO
OH
l
k
j
i
h
g
f
e
d
c
b
a
0.94
0.99
1.00
0.97
1.30
0.98
0.98
0.99
1.97
2.00
1.98
2.03
2.01
1.98
1.97
2.00
10.0 9.5 9.0 8.5 8.0 7.5 7.0 6.5 6.0 5.5 5.0 4.5 4.0 3.5 3.0 2.5 2.0 1.5 1.0 0.5 0.0
f1 (ppm)

6c

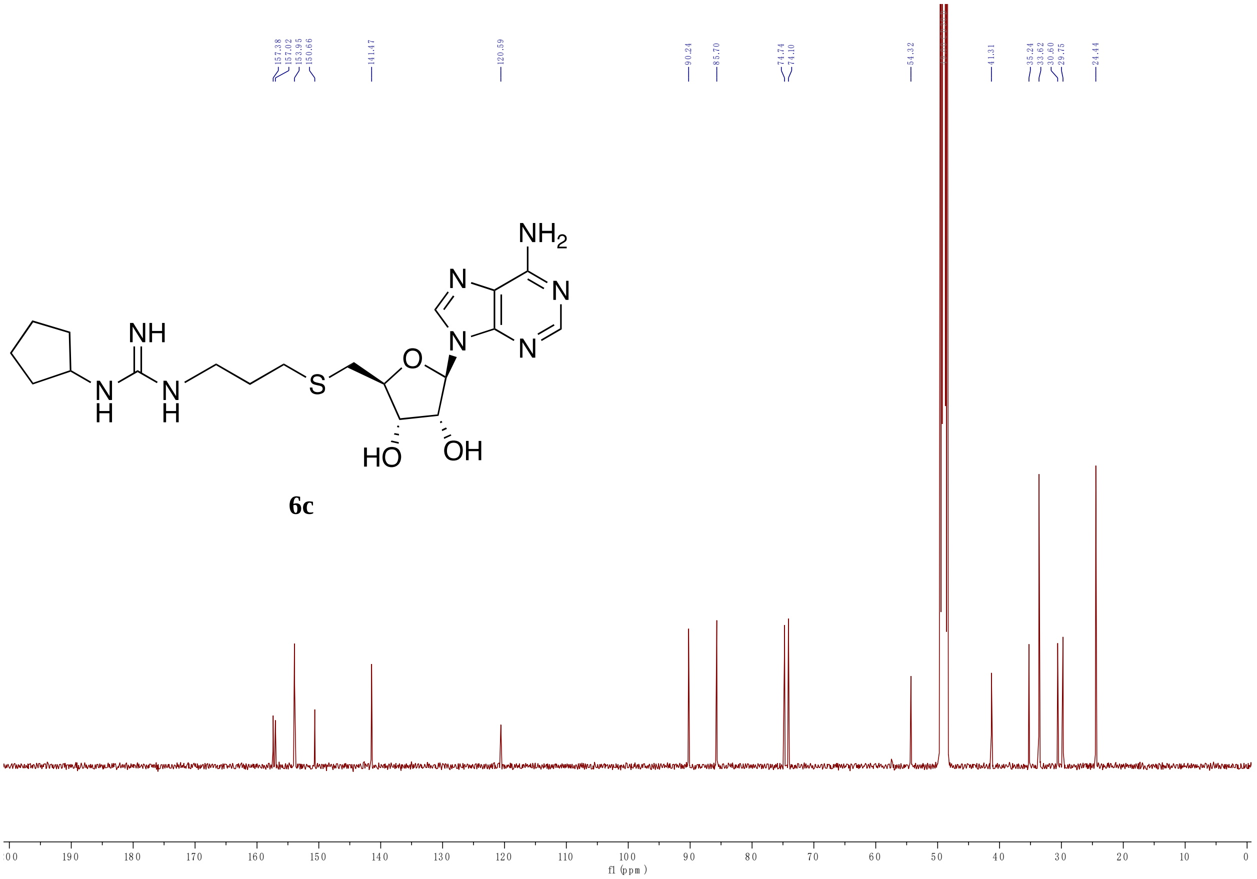

HRMS Spectra of 6c

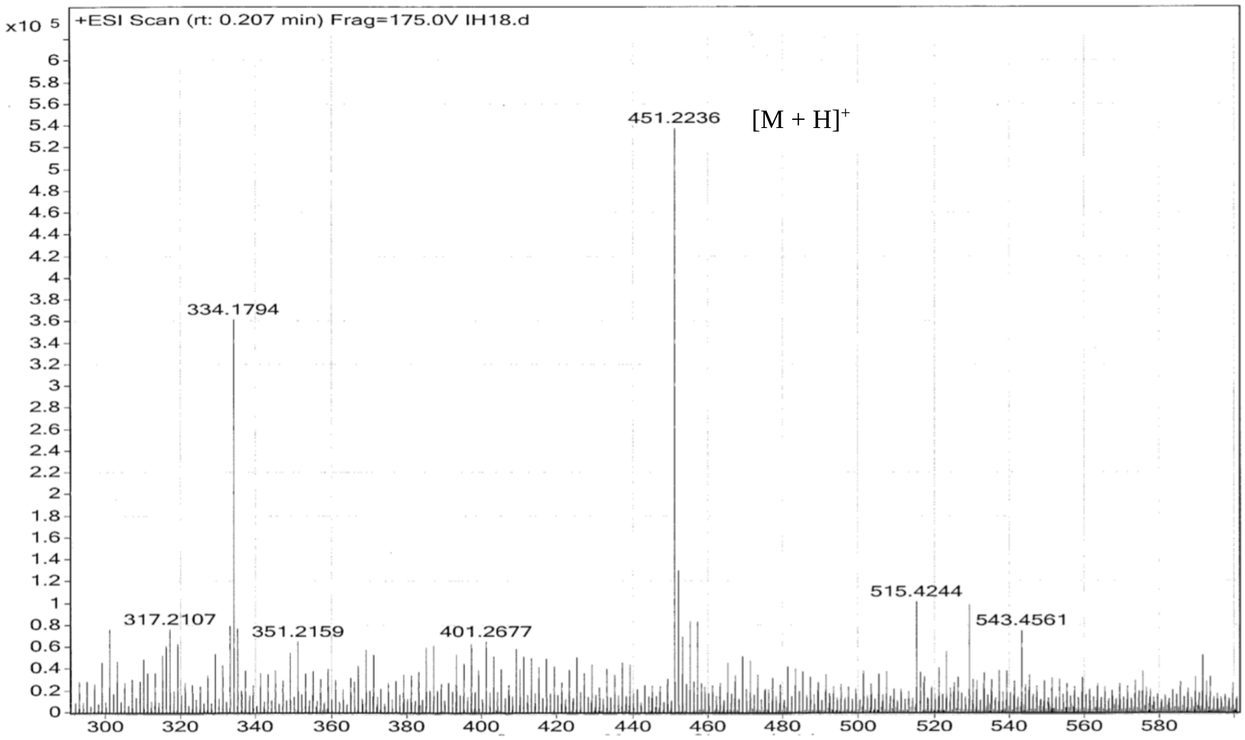

HPLC Spectra of 6c

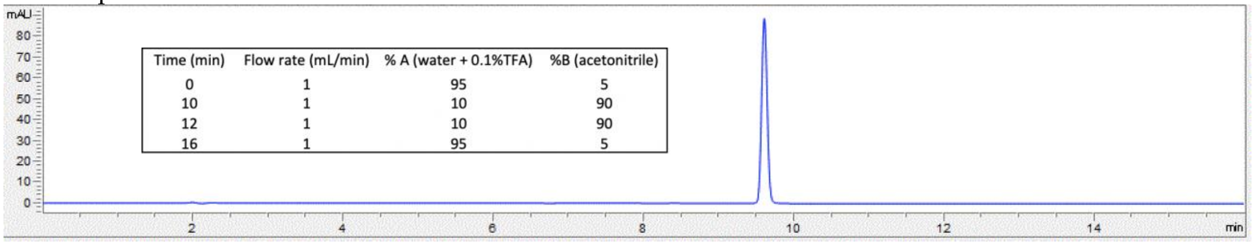

Time (min)	Flow rate (mL/min)	% A (water + 0.1%TFA)	%B (acetonitrile)
0	1	95	5
10	1	10	90
12	1	10	90
16	1	95	5

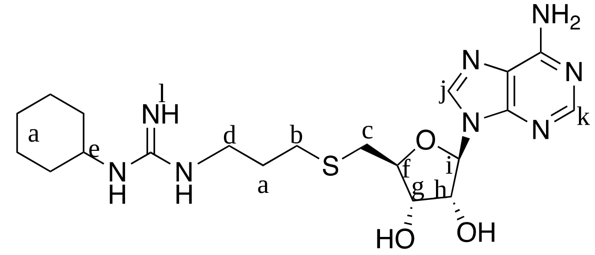
NH2
N
N
j
N
N
k
l
NH
a
e
N
H
N
H
d
b
a
S
c
O
f
i
g
h
HO
OH

6d

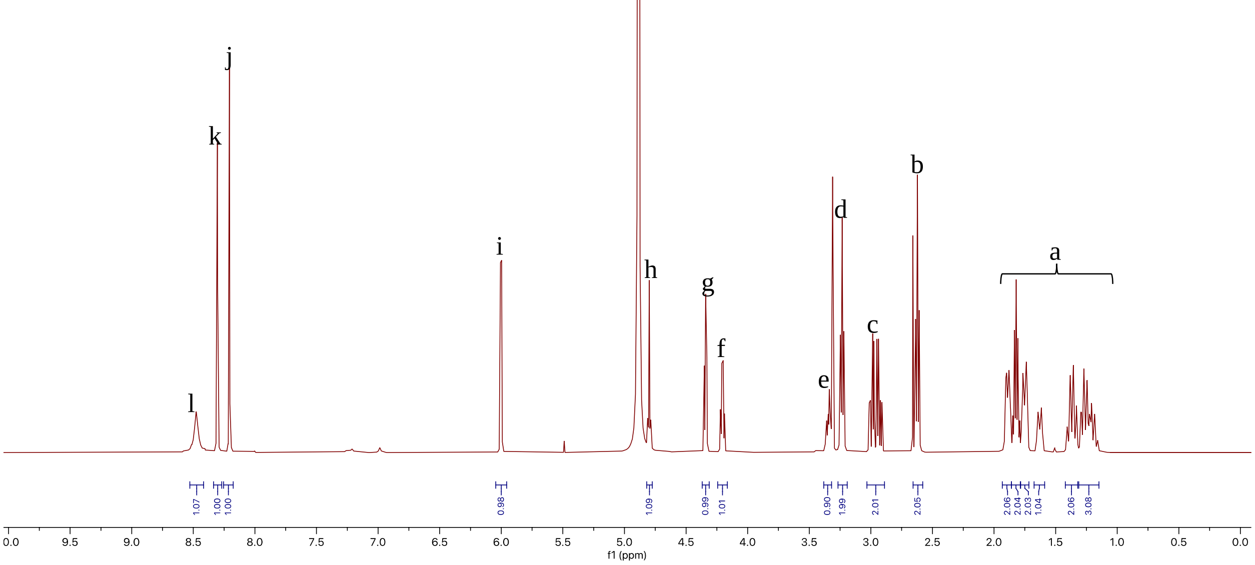
j
k
l
i
h
g
f
e
d
c
b
a
1.07
1.00
1.00
0.98
1.09
0.99
1.01
0.90
1.99
2.01
2.05
2.06
2.04
2.03
1.04
2.06
3.08
0.0 9.5 9.0 8.5 8.0 7.5 7.0 6.5 6.0 5.5 5.0 4.5 4.0 3.5 3.0 2.5 2.0 1.5 1.0 0.5 0.0
f1 (ppm)

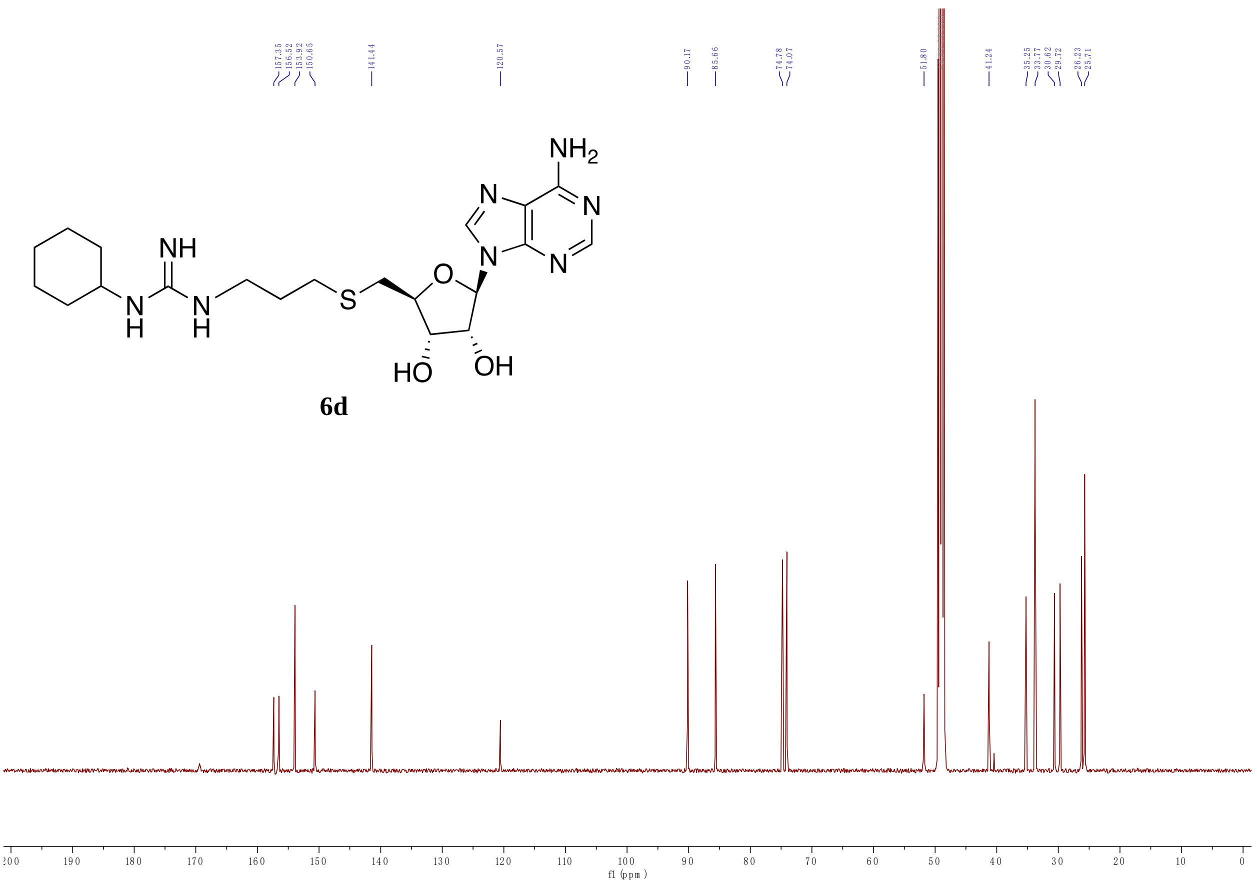

HRMS Spectra of 6d

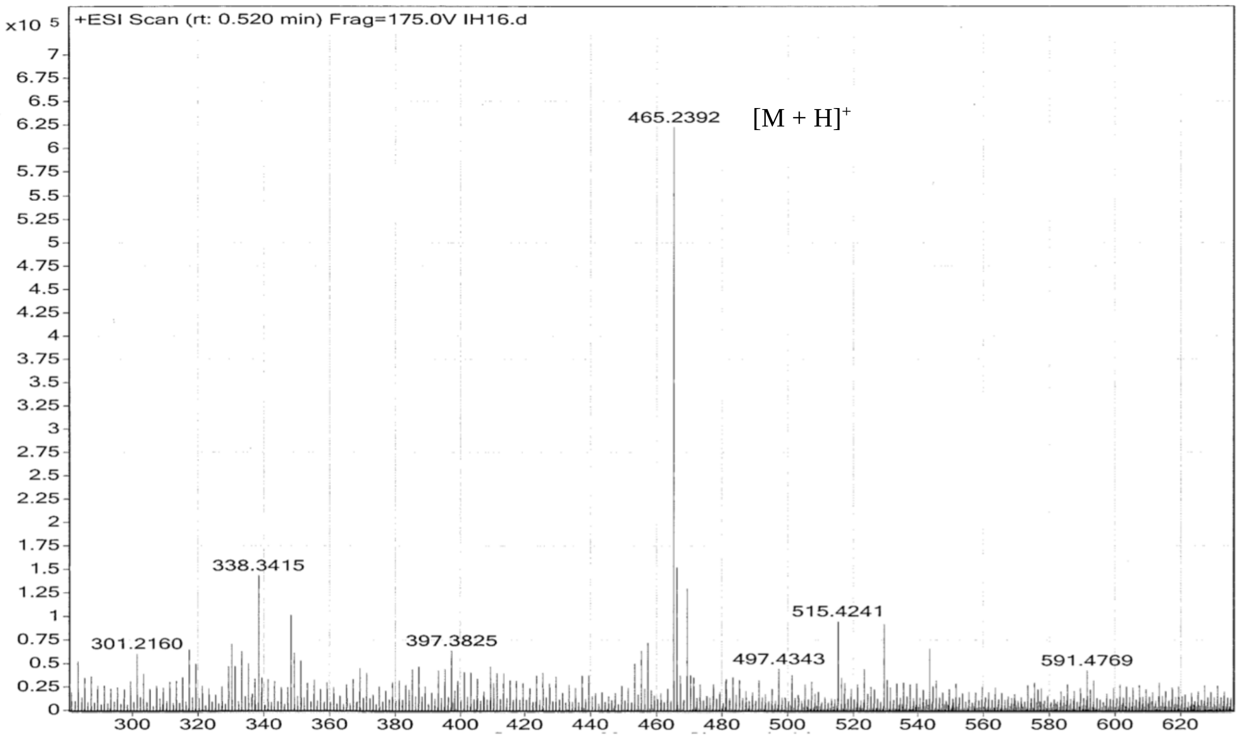

HPLC Spectra of 6d

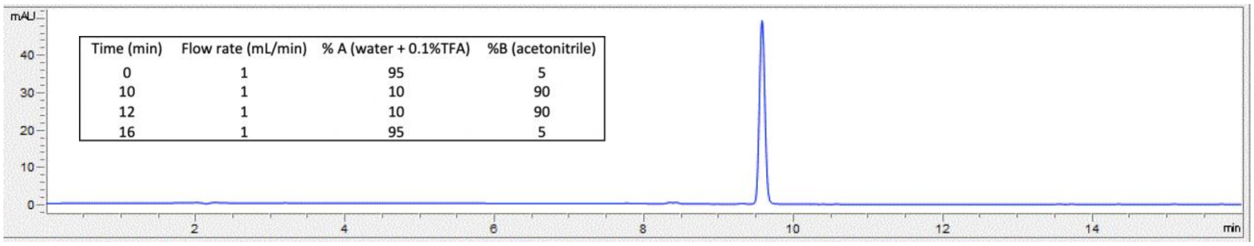

Time (min)	Flow rate (mL/min)	% A (water + 0.1%TFA)	%B (acetonitrile)
0	1	95	5
10	1	10	90
12	1	10	90
16	1	95	5

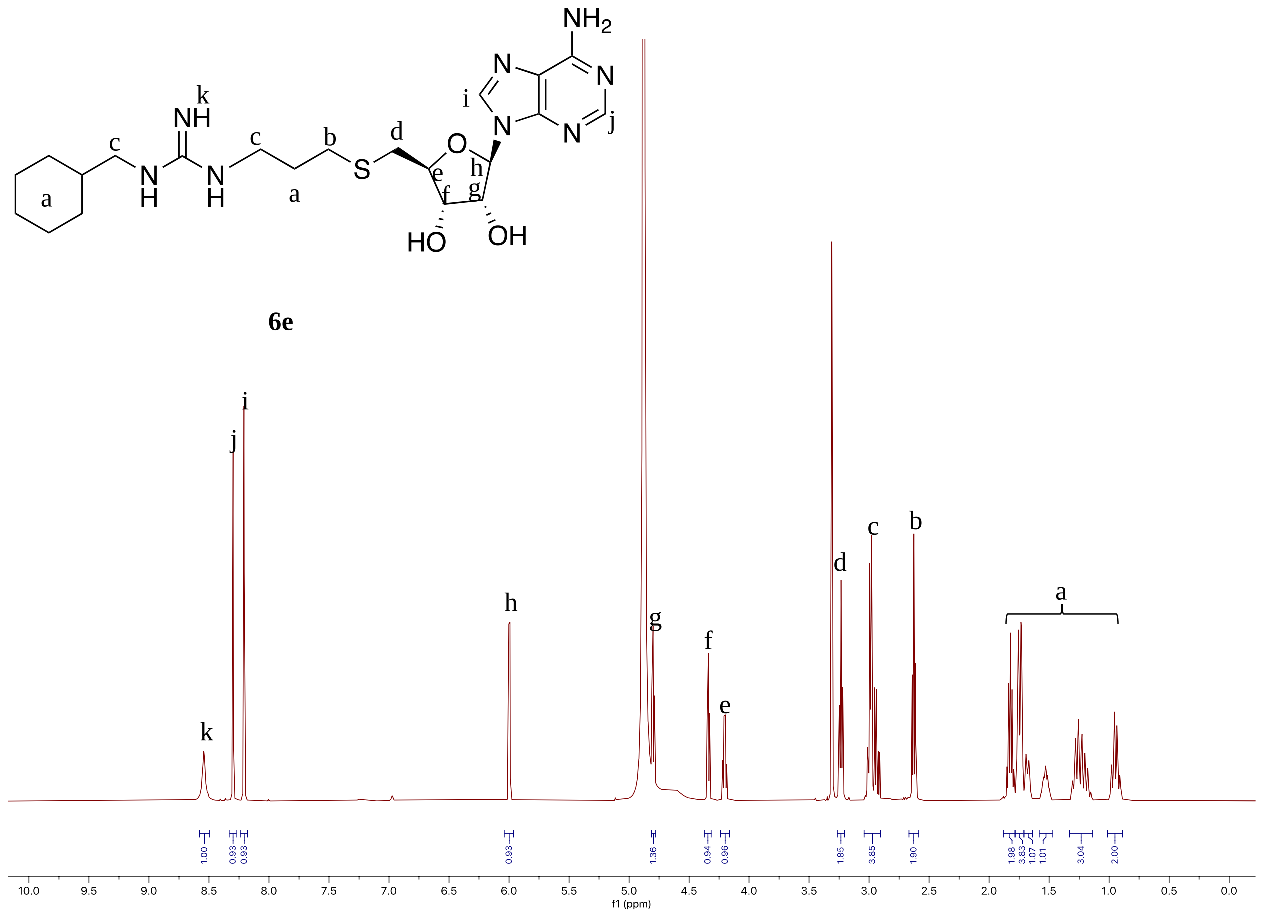

NH2
N
N
i
N
N
j
k
NH
c
c
b
d
O
h
N
H
N
H
S
e
a
a
f
g
HO
OH
6e
k
j
i
h
g
f
e
d
c
b
a
1.00
0.93
0.93
0.93
1.36
0.94
0.96
1.85
3.85
1.90
1.98
3.83
1.07
1.01
3.04
2.00
10.0 9.5 9.0 8.5 8.0 7.5 7.0 6.5 6.0 5.5 5.0 4.5 4.0 3.5 3.0 2.5 2.0 1.5 1.0 0.5 0.0
f1 (ppm)

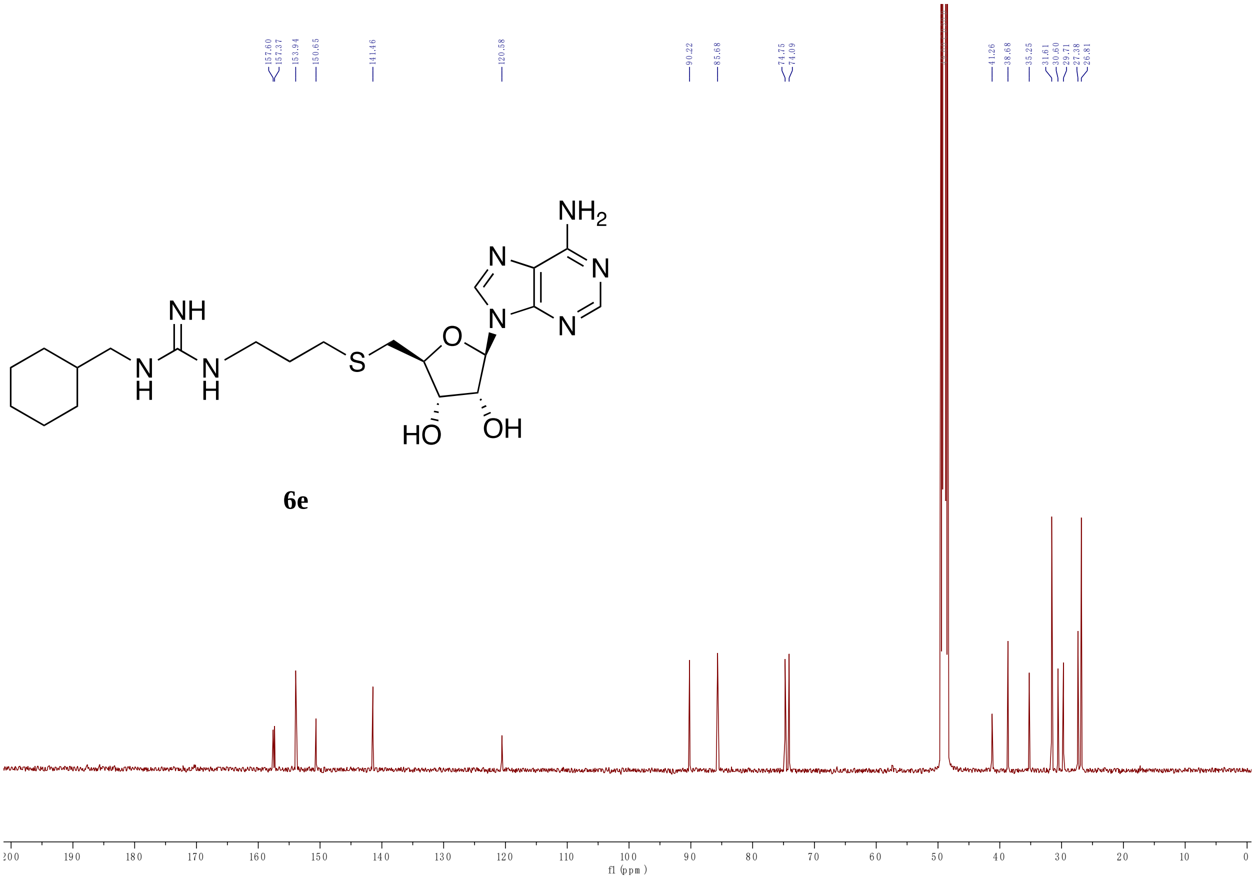

HRMS Spectra of 6e

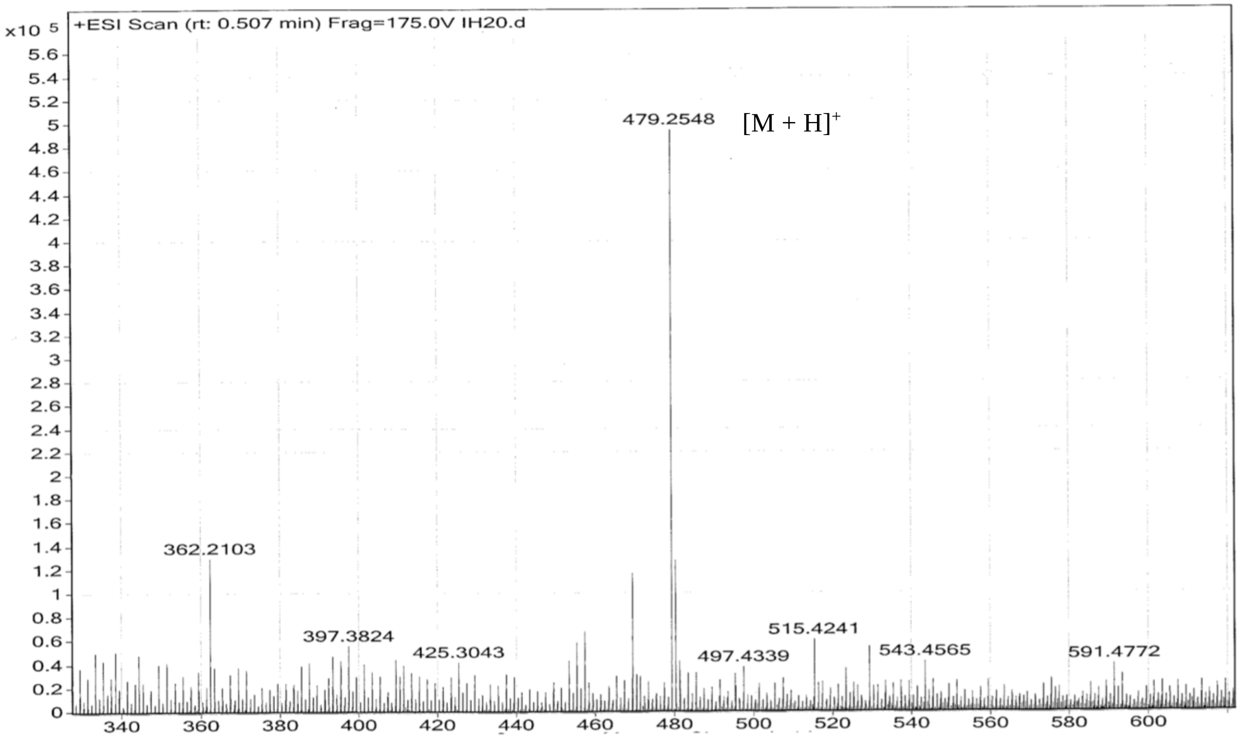

HPLC Spectra of 6e

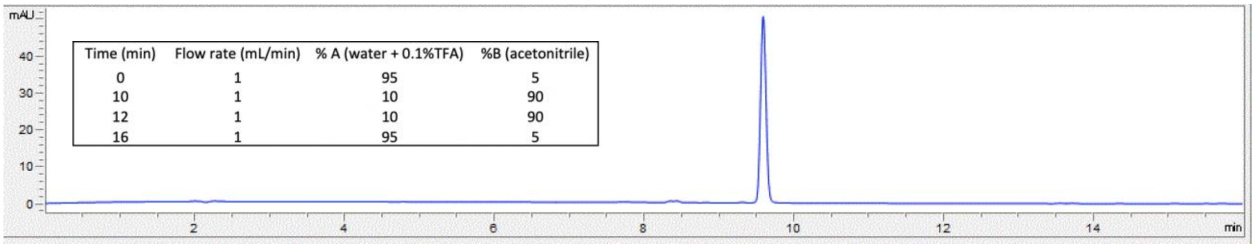

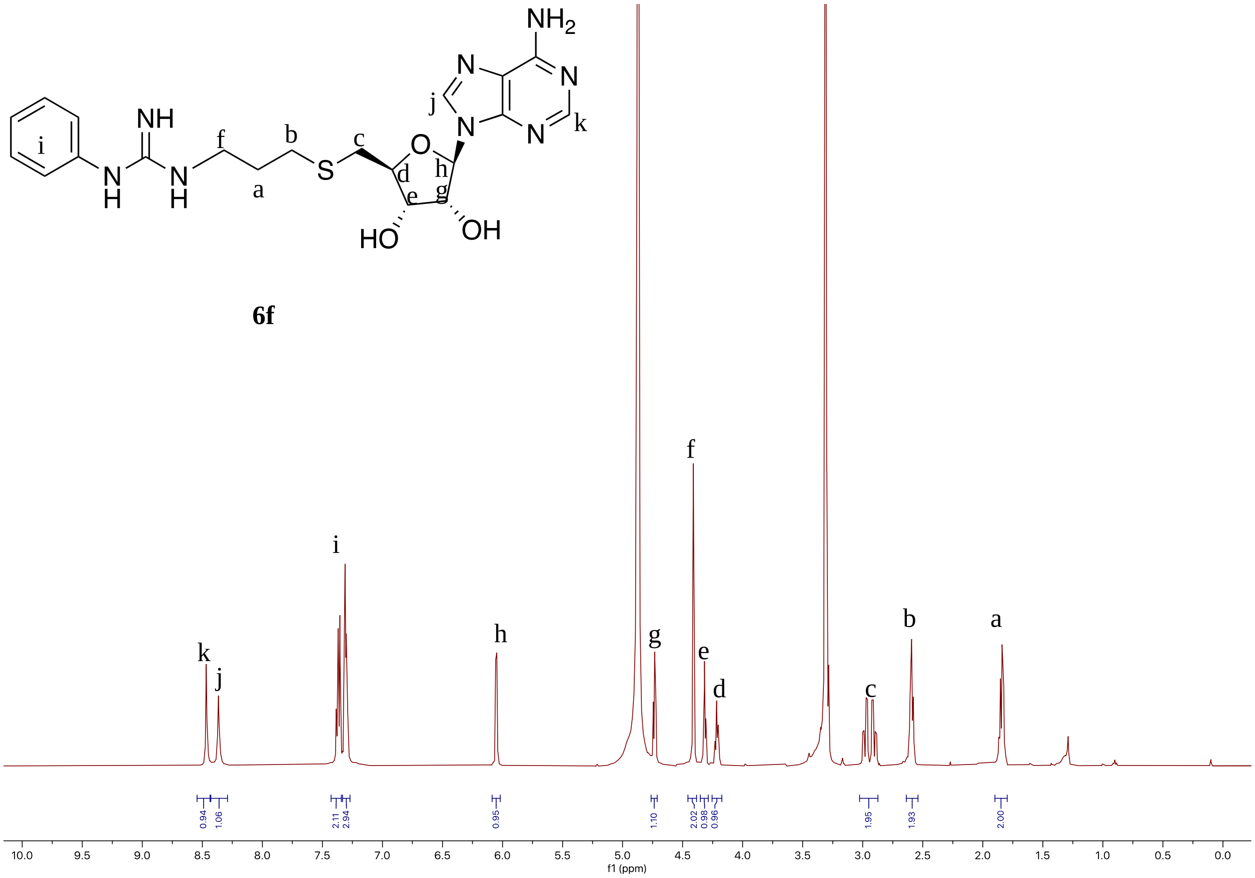
NH2
N
N
j
N
k
N
NH
i
N
H
N
H
f
b
a
S
c
O
d
h
e
g
HO
OH
6f
k
j
i
h
g
f
e
d
c
b
a
0.94
1.06
2.11
2.94
0.95
1.10
2.02
0.98
0.96
1.95
1.93
2.00
10.0
9.5
9.0
8.5
8.0
7.5
7.0
6.5
6.0
5.5
5.0
4.5
4.0
3.5
3.0
2.5
2.0
1.5
1.0
0.5
0.0
f1 (ppm)

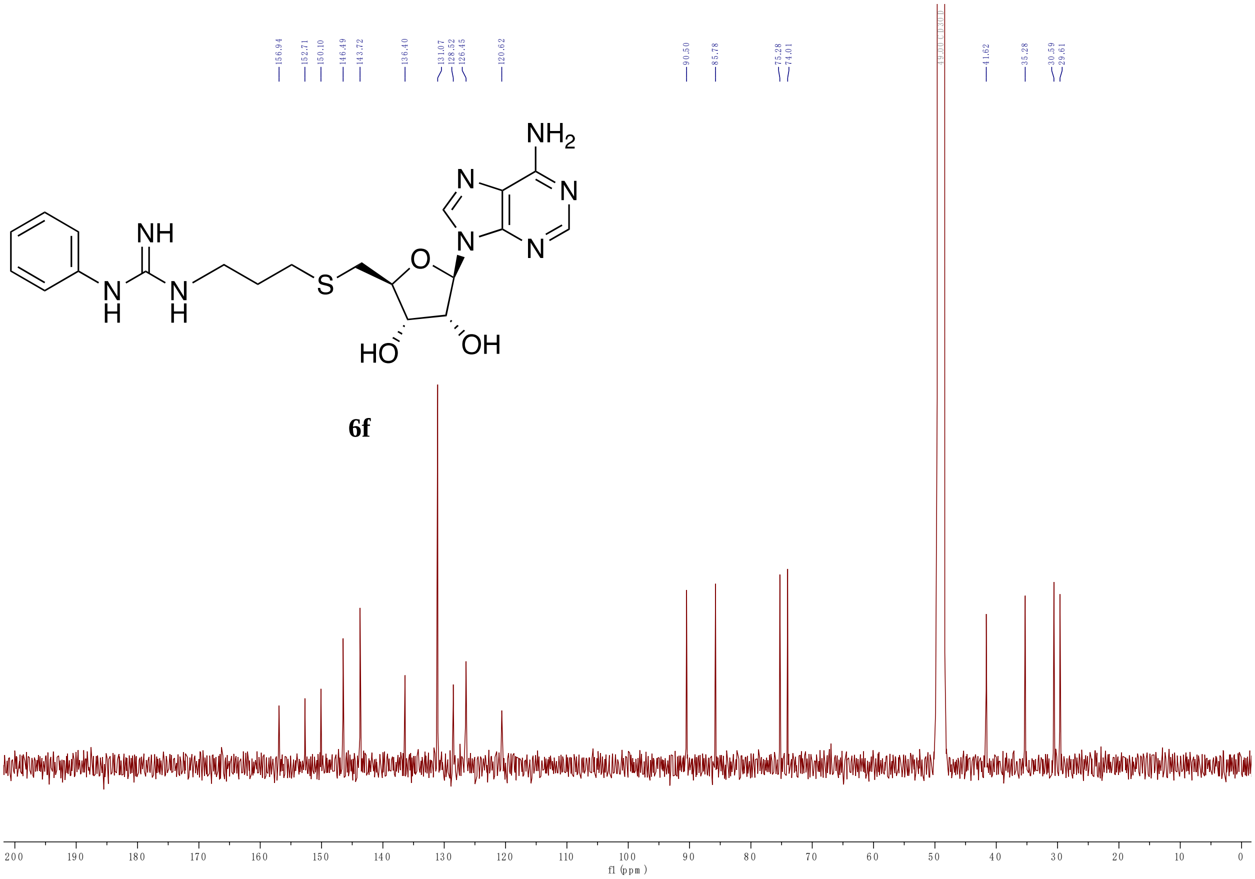

HRMS Spectra of 6f

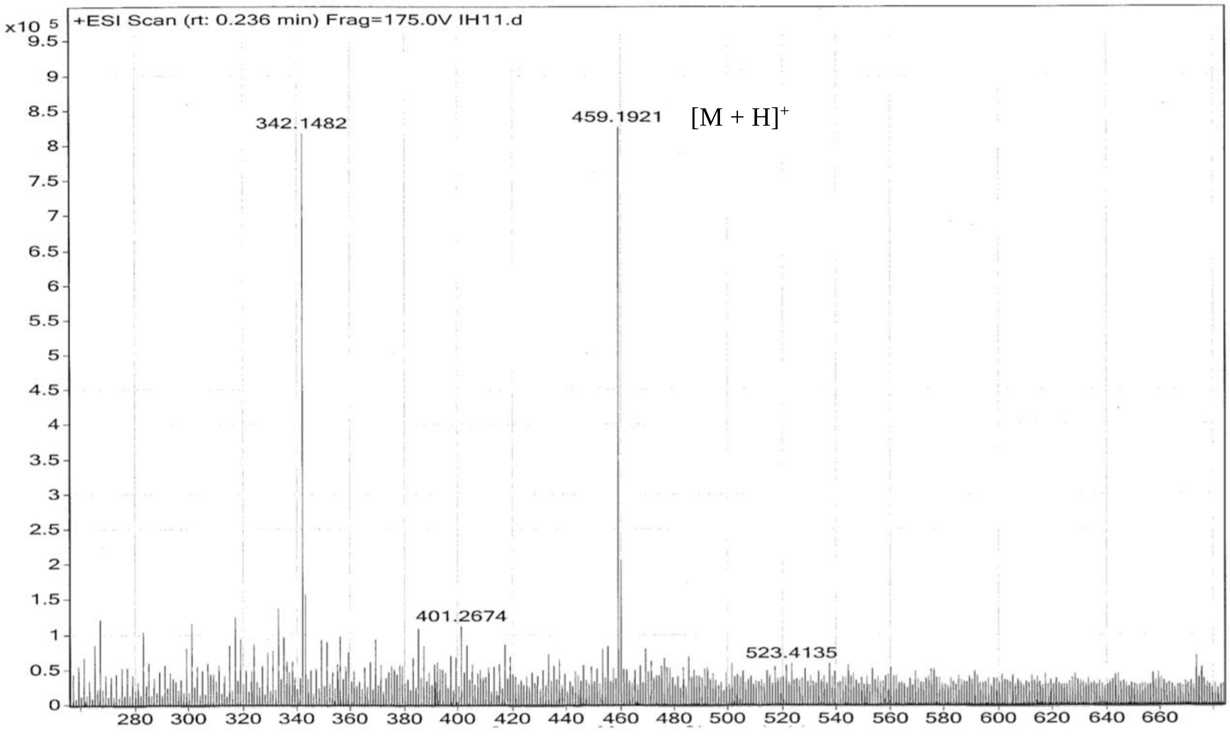

HPLC Spectra of 6f

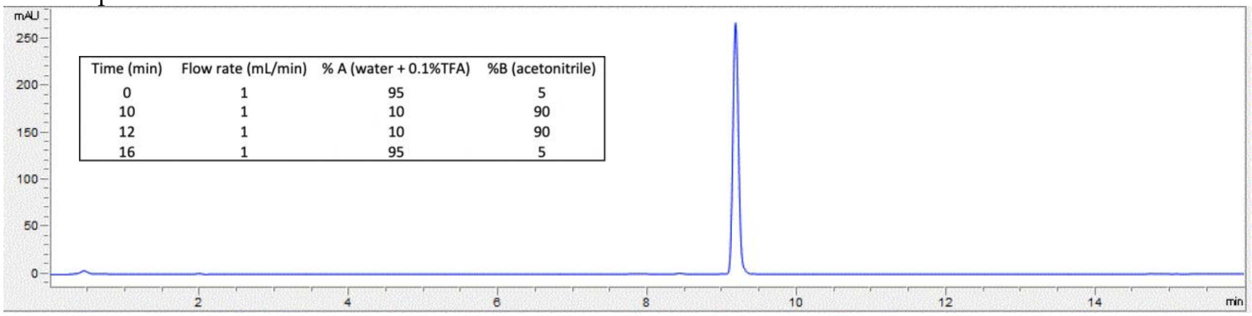

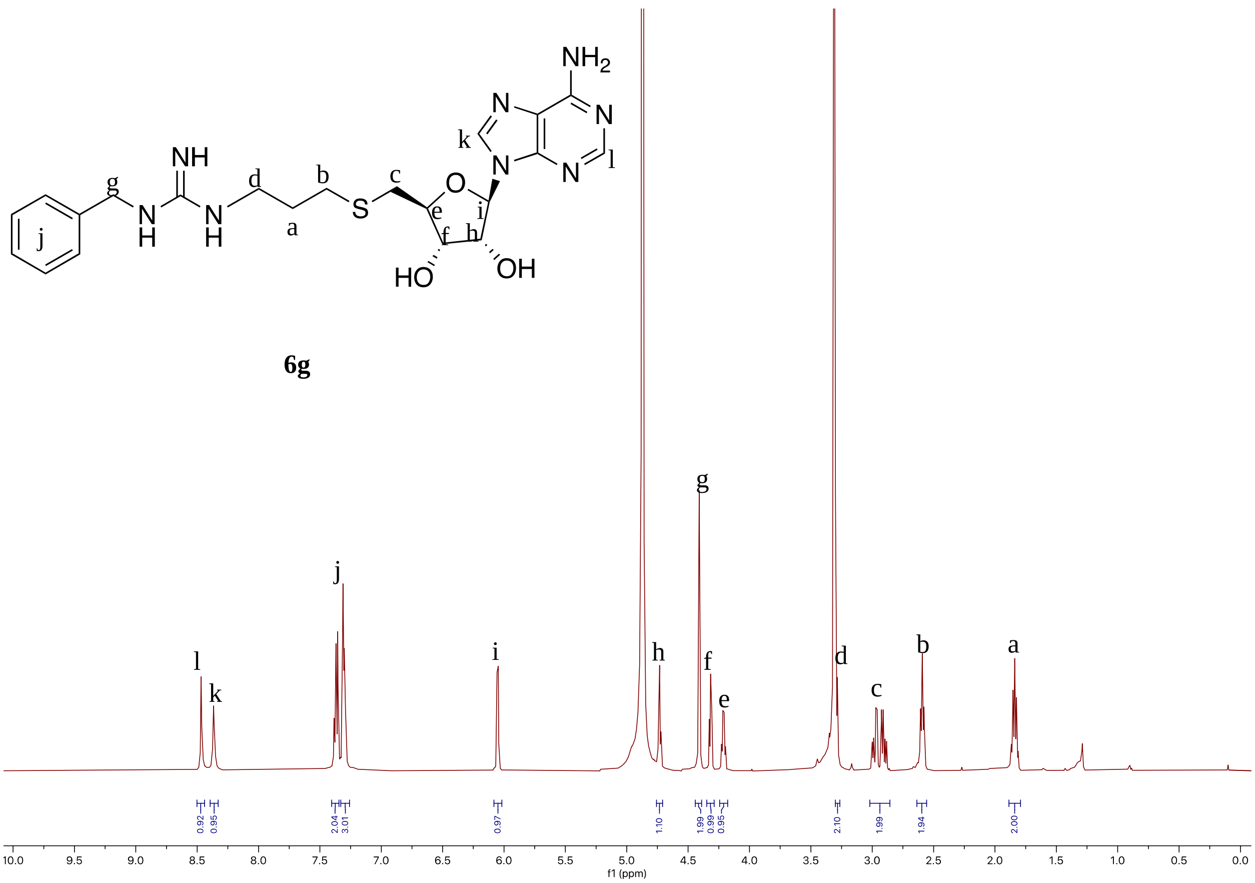
NH2
N
N
k
N
N
l
NH
g
d
b
c
O
i
N
H
N
H
S
e
j
a
f
h
HO
OH
l
k
j
i
h
g
f
e
d
c
b
a
0.92
0.95
2.04
3.01
0.97
1.10
1.99
0.99
0.95
2.10
1.99
1.94
2.00
10.0
9.5
9.0
8.5
8.0
7.5
7.0
6.5
6.0
5.5
5.0
4.5
4.0
3.5
3.0
2.5
2.0
1.5
1.0
0.5
0.0
f1 (ppm)

6g

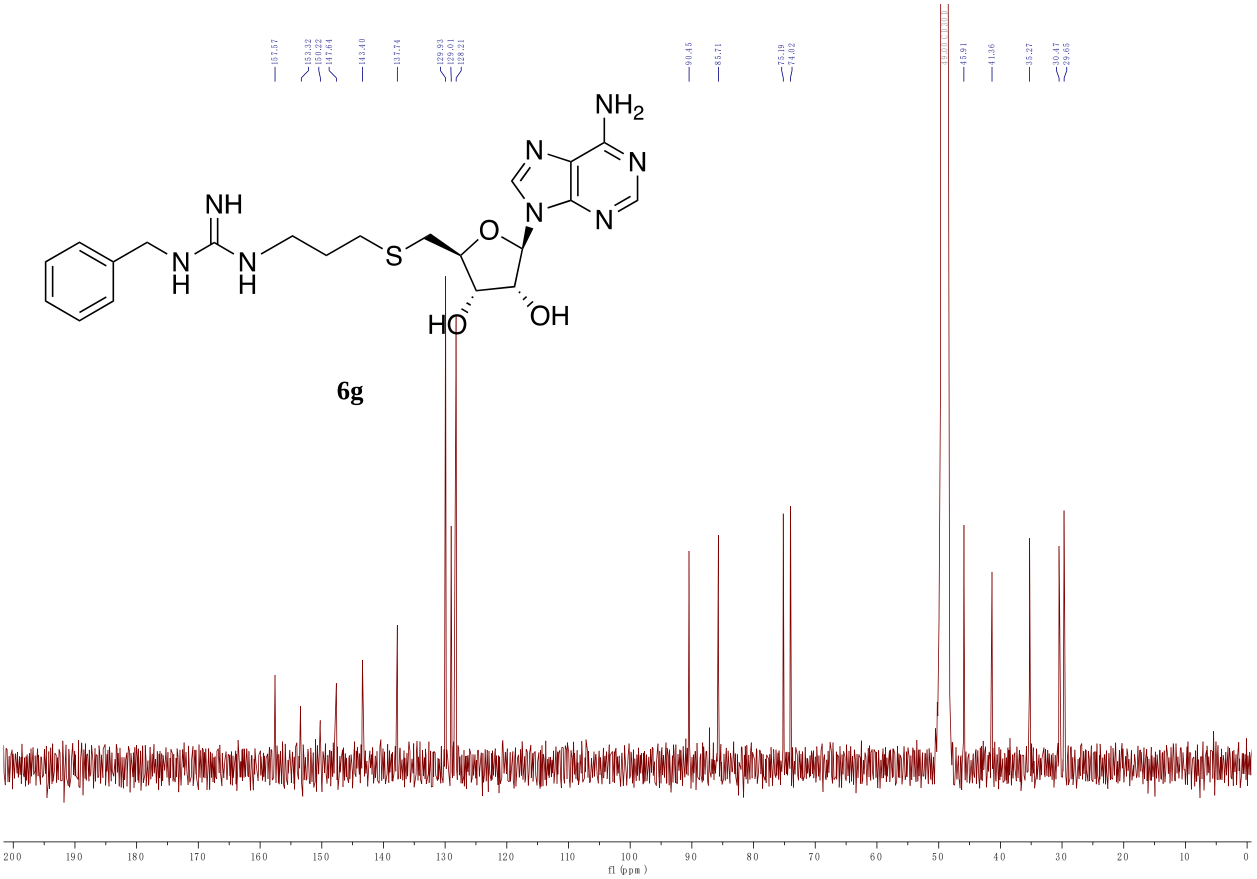

HRMS Spectra of 6g

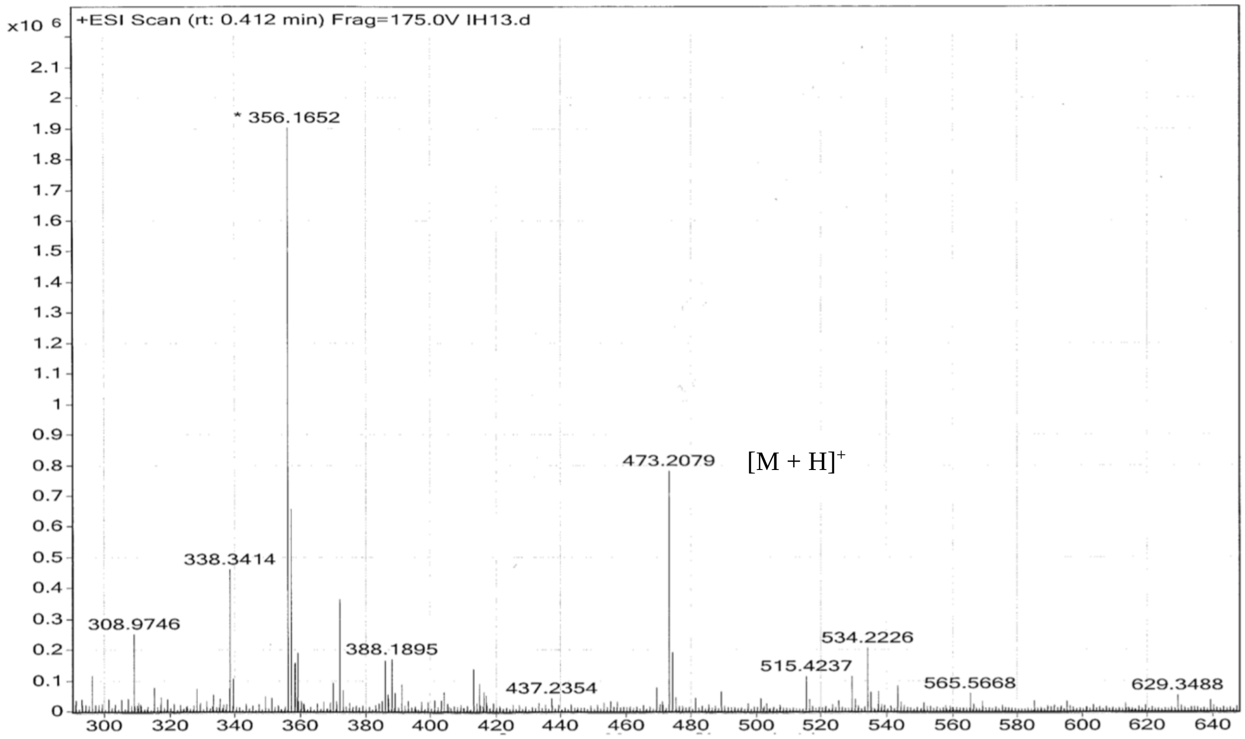

HPLC Spectra of 6g

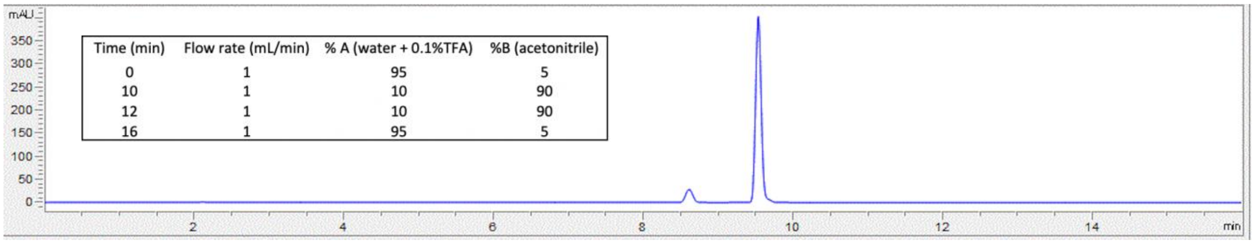

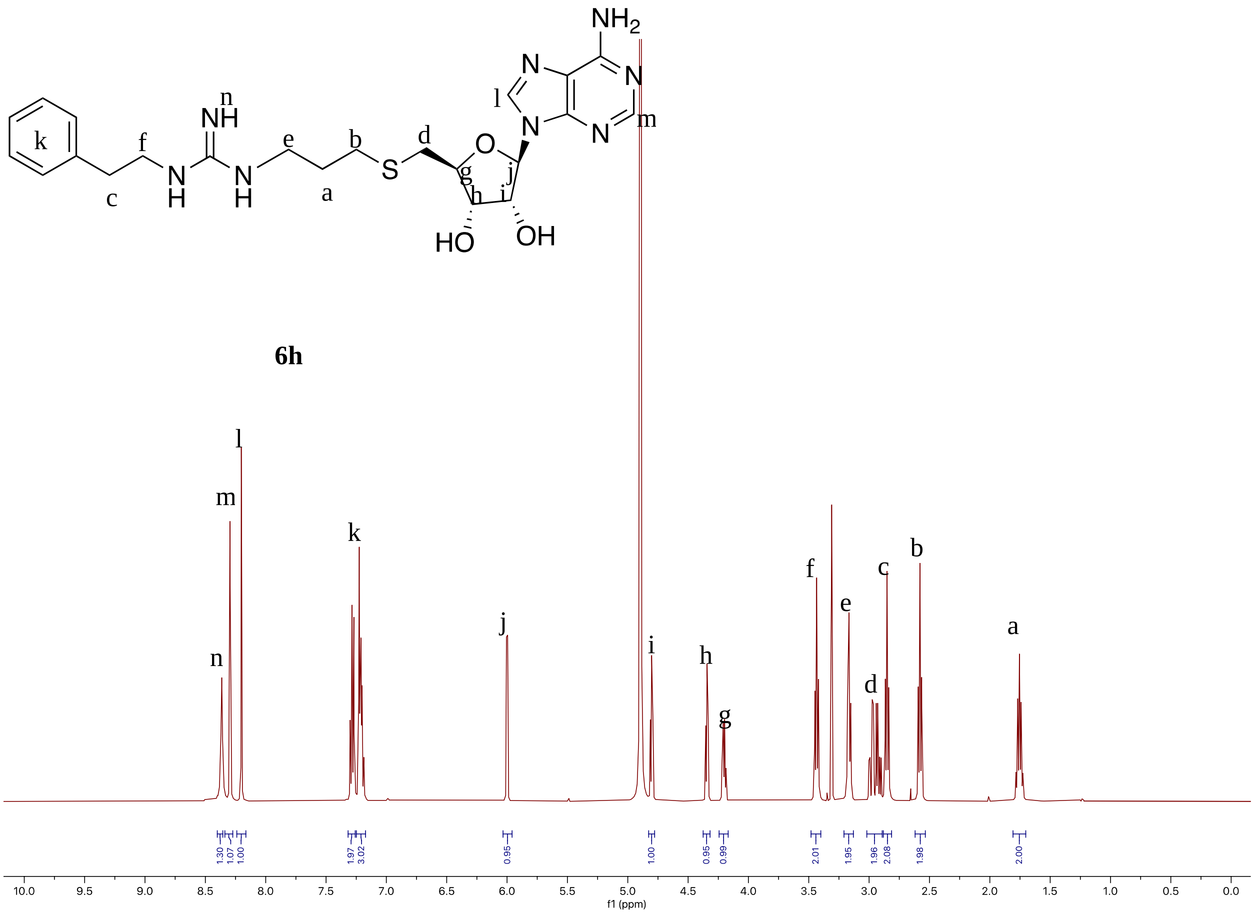
NH2
N
N
N
N
N
O
S
HO
OH
NH
N
H
N
H
n
m
l
k
j
i
h
g
f
e
d
c
b
a
1.30
1.07
1.00
1.97
3.02
0.95
1.00
0.95
0.99
2.01
1.95
1.96
2.08
1.98
2.00
10.0
9.5
9.0
8.5
8.0
7.5
7.0
6.5
6.0
5.5
5.0
4.5
4.0
3.5
3.0
2.5
2.0
1.5
1.0
0.5
0.0
f1 (ppm)

6h

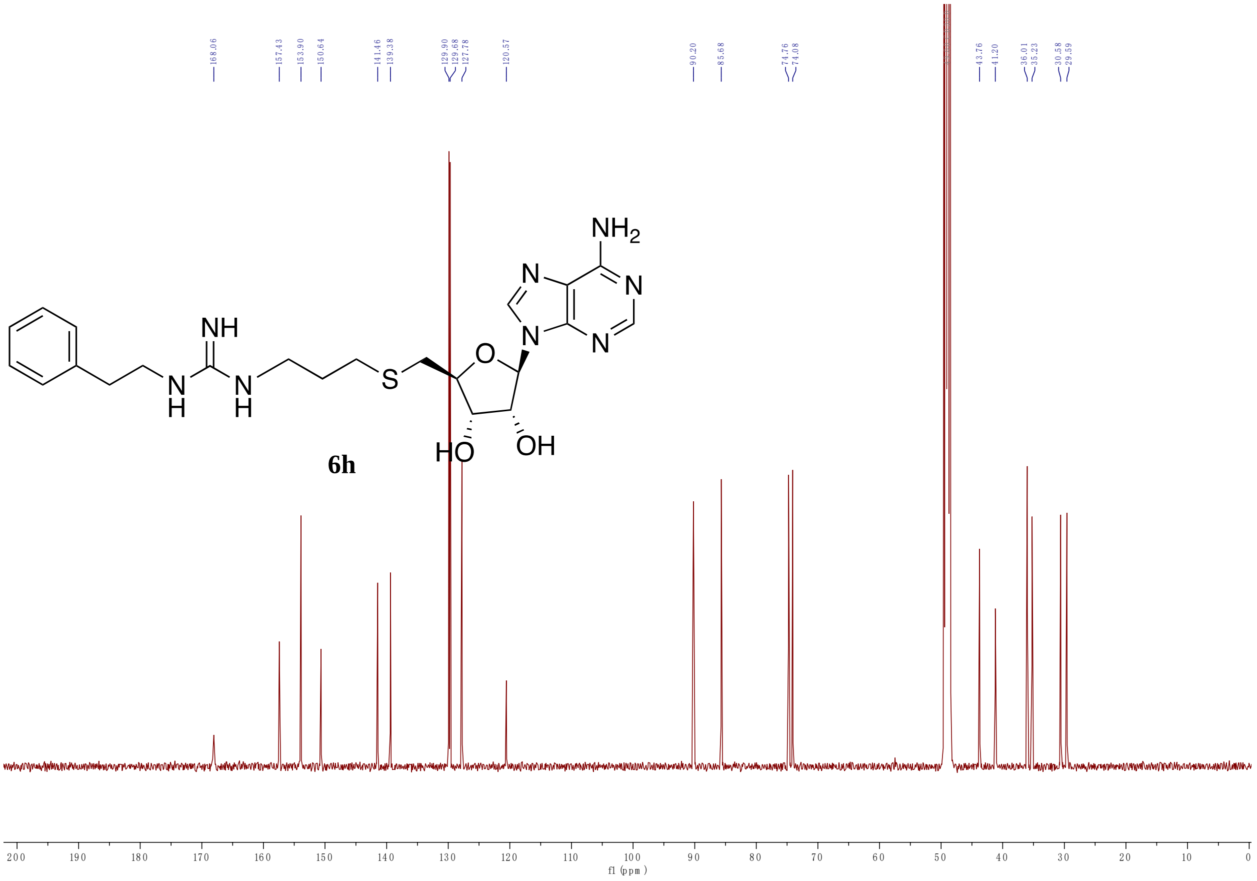

HRMS Spectra of 6h

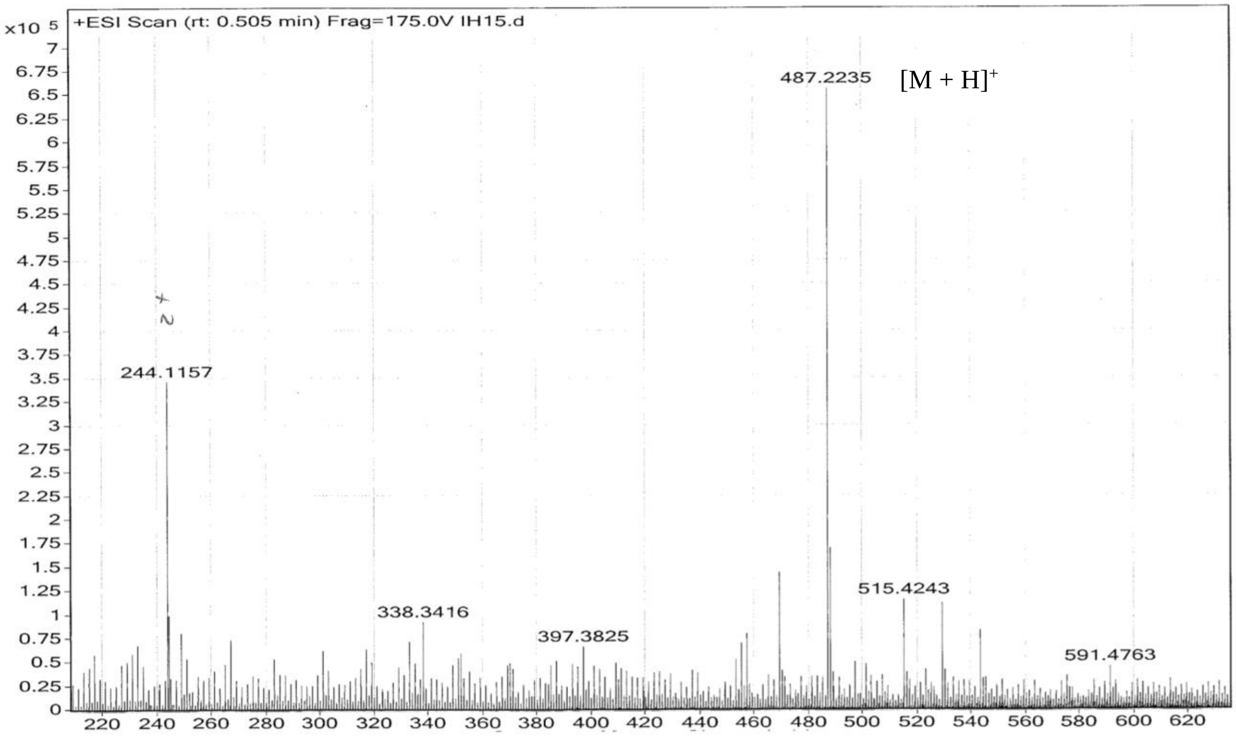

HPLC Spectra of 6h

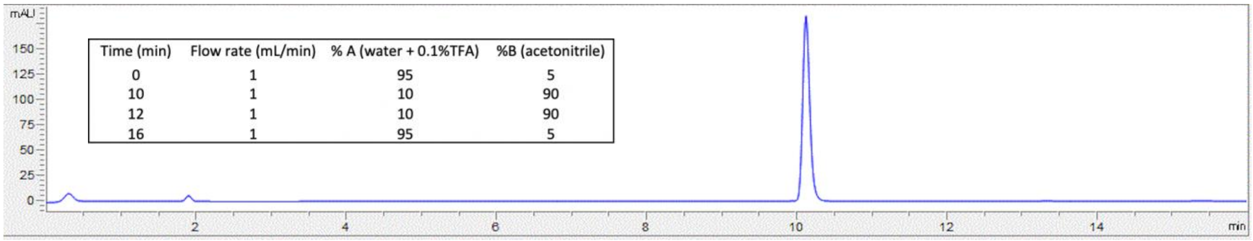

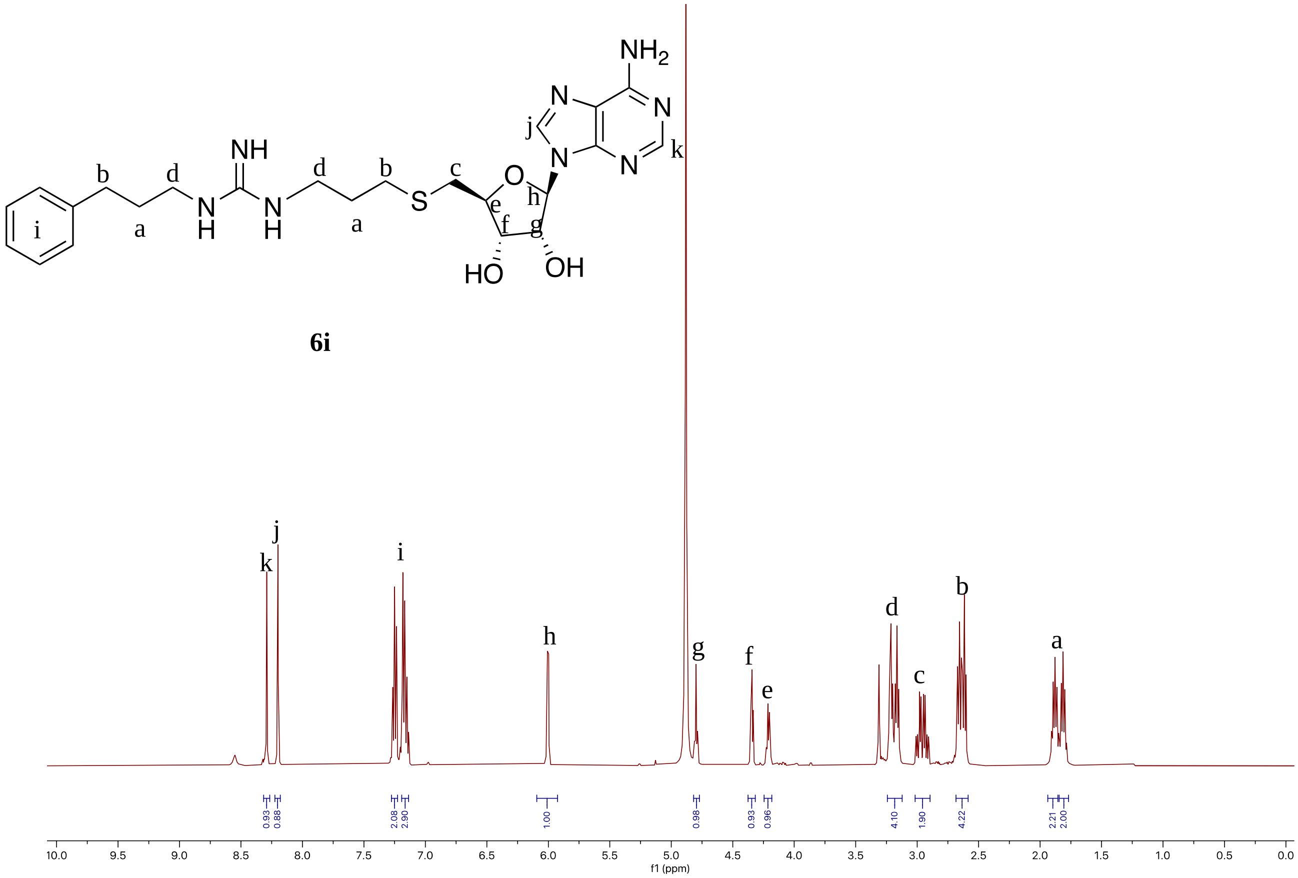
NH
NH2
N
N
N
N
N
O
S
HO
OH
H
H
a b c d e f g h i j k
0.93
0.88
2.08
2.90
1.00
0.98
0.93
0.96
4.10
1.90
4.22
2.21
2.00
10.0 9.5 9.0 8.5 8.0 7.5 7.0 6.5 6.0 5.5 5.0 4.5 4.0 3.5 3.0 2.5 2.0 1.5 1.0 0.5 0.0
f1 (ppm)

6i

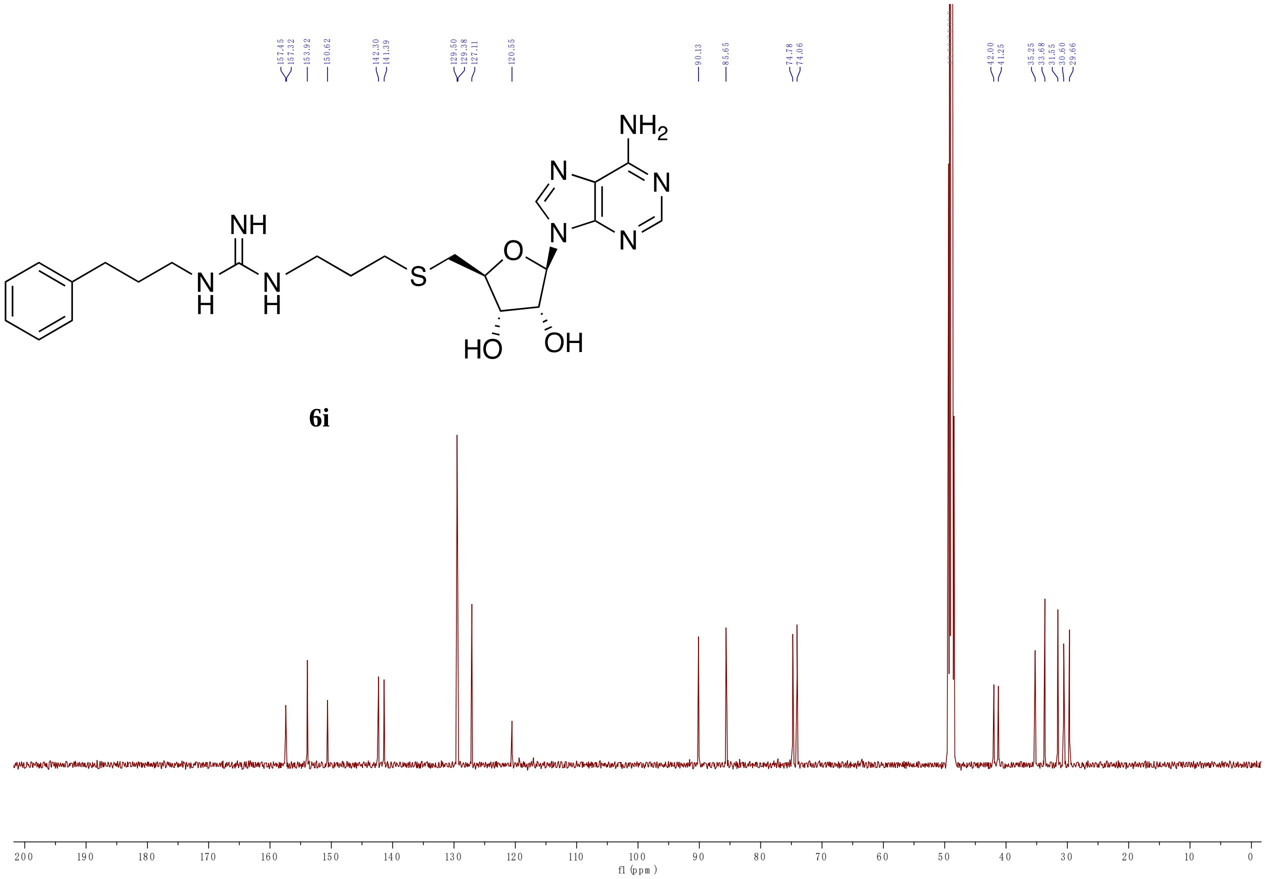

HRMS Spectra of 6i

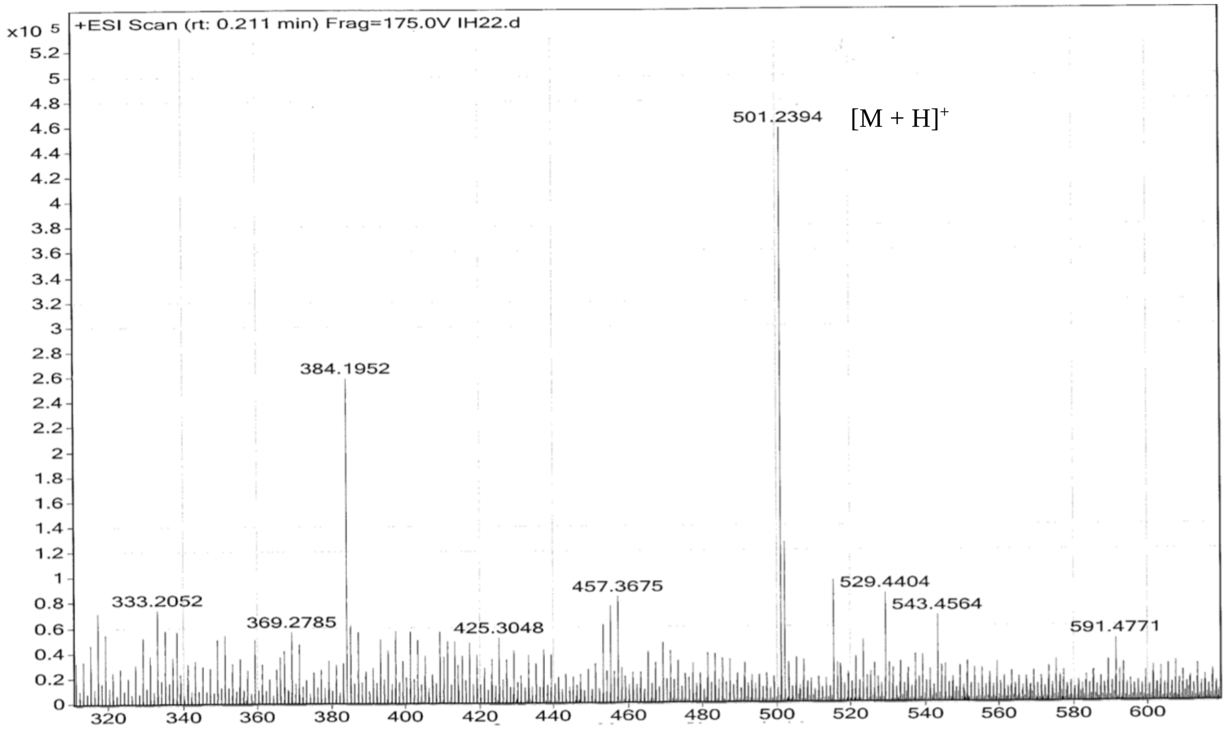

HPLC Spectra of 6i

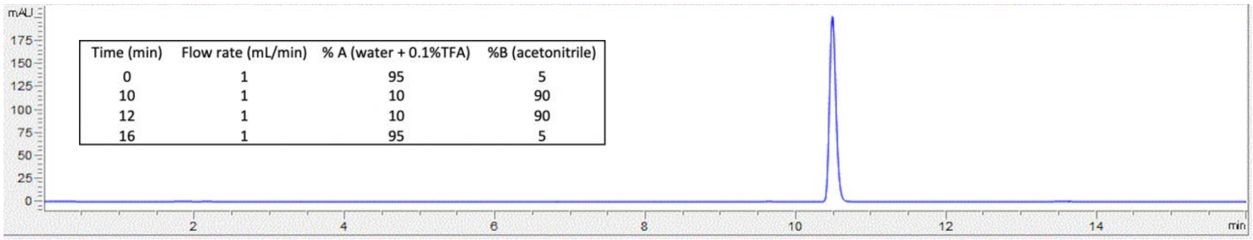

Time (min)	Flow rate (mL/min)	% A (water + 0.1%TFA)	%B (acetonitrile)
0	1	95	5
10	1	10	90
12	1	10	90
16	1	95	5

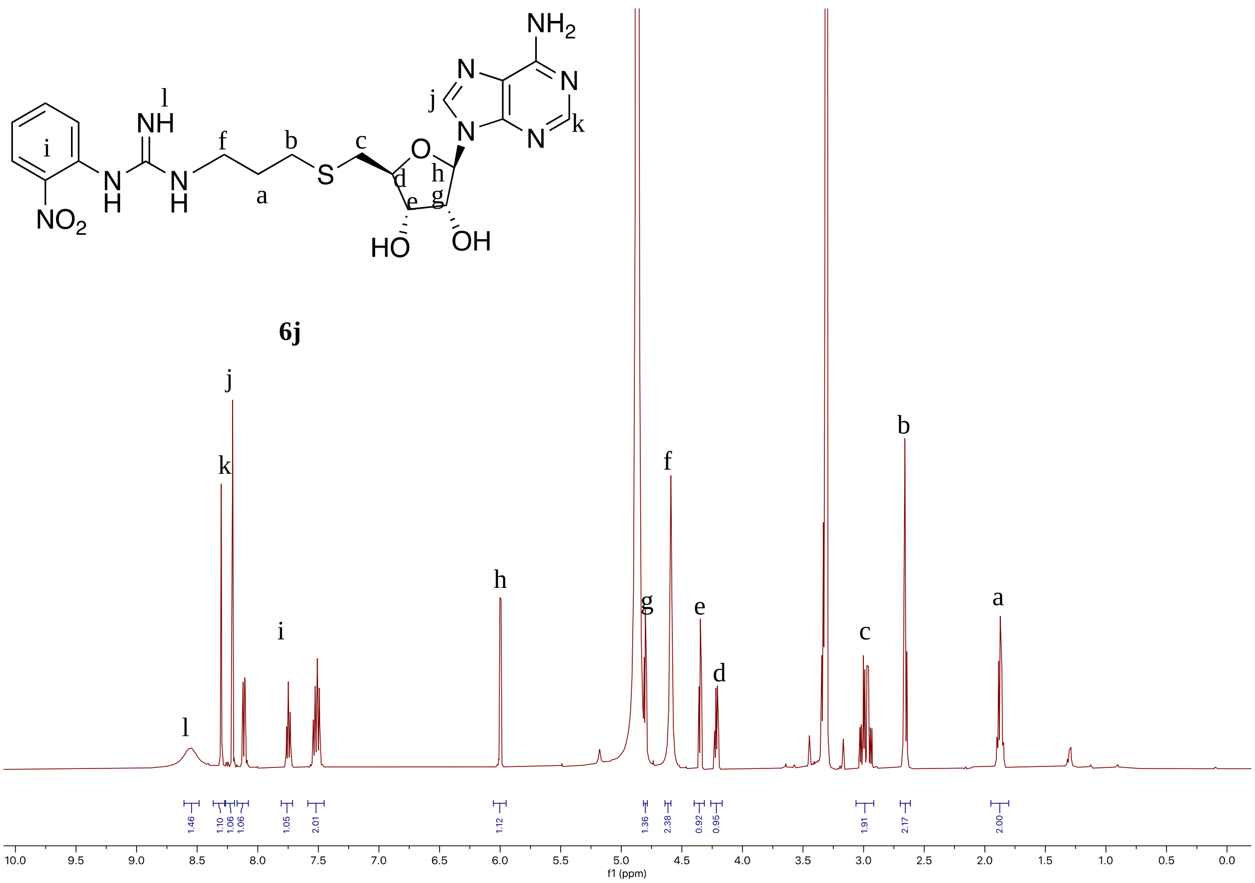
NH2
N
N
N
N
N
j
k
l
NH
i
N
H
N
H
NO2
f
b
a
S
c
O
h
d
e
g
HO
OH
6j
j
k
b
f
h
i
g
e
d
c
a
l
1.46
1.10
1.06
1.06
1.05
2.01
1.12
1.36
2.38
0.92
0.95
1.91
2.17
2.00
10.0
9.5
9.0
8.5
8.0
7.5
7.0
6.5
6.0
5.5
5.0
4.5
4.0
3.5
3.0
2.5
2.0
1.5
1.0
0.5
0.0
f1 (ppm)

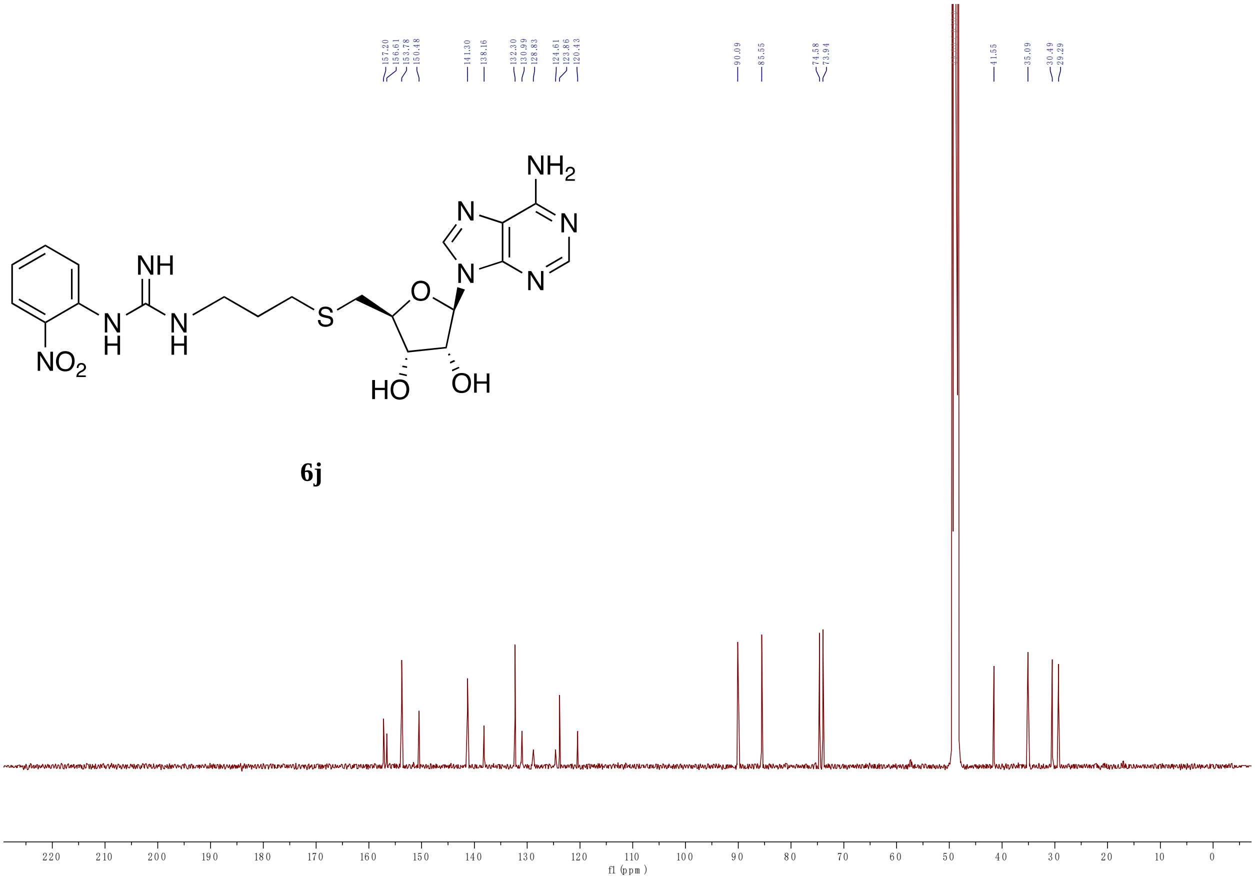

6j

HRMS Spectra of 6j

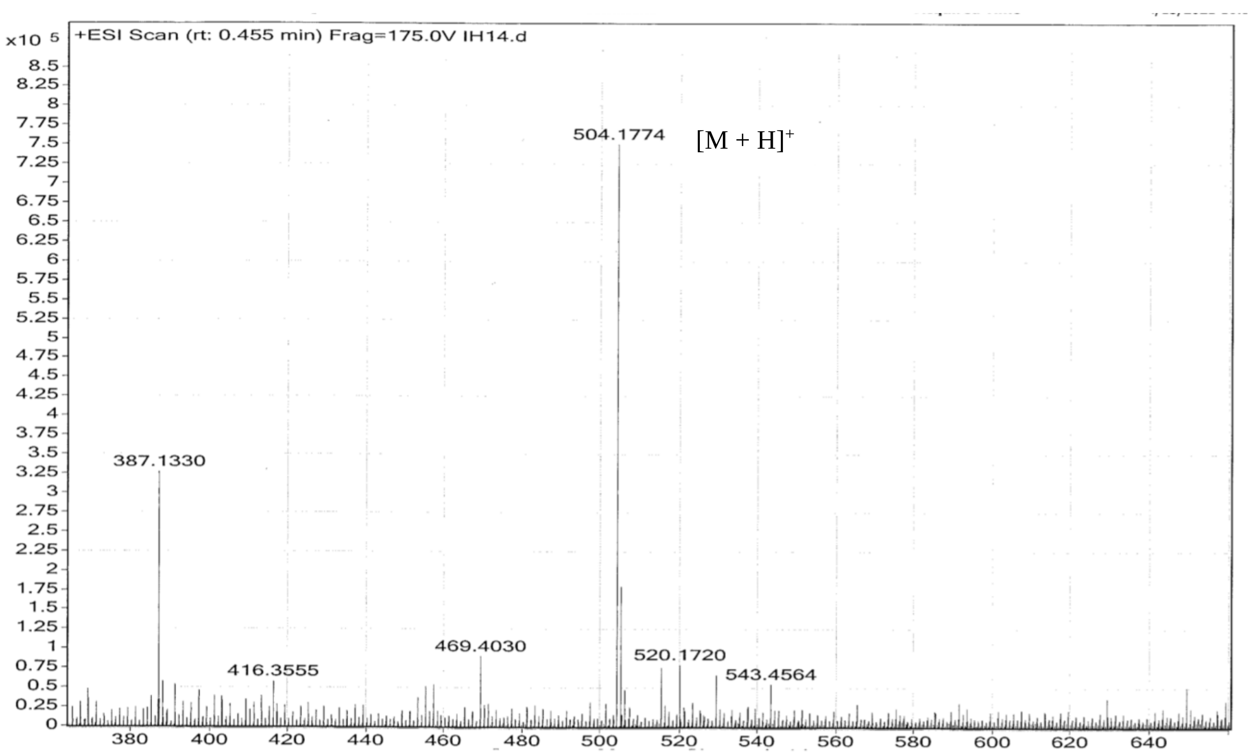

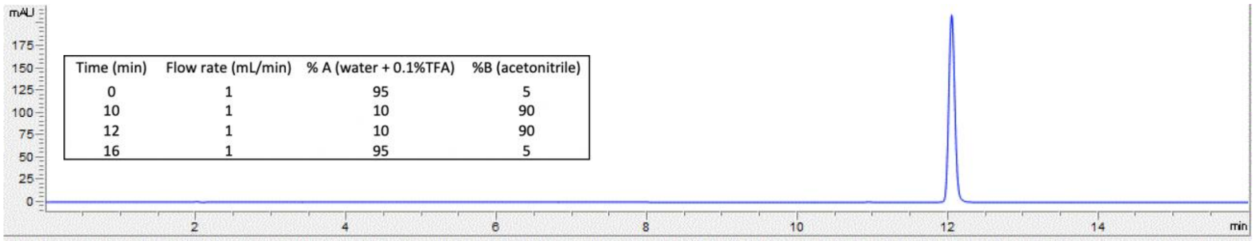

Time (min)	Flow rate (mL/min)	% A (water + 0.1%TFA)	%B (acetonitrile)
0	1	95	5
10	1	10	90
12	1	10	90
16	1	95	5

HPLC Spectra of 6j

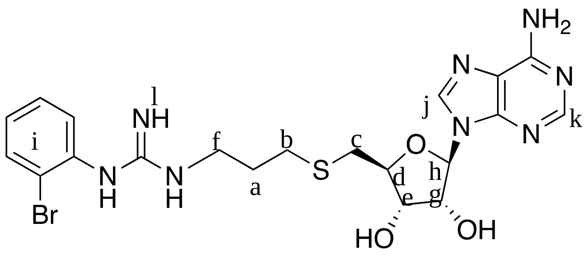
NH$_2$
N
N
j
N
N
k
N
O
c
S
b
f
NH
l
i
N
H
N
H
a
d
h
e
g
HO
OH
Br

6k

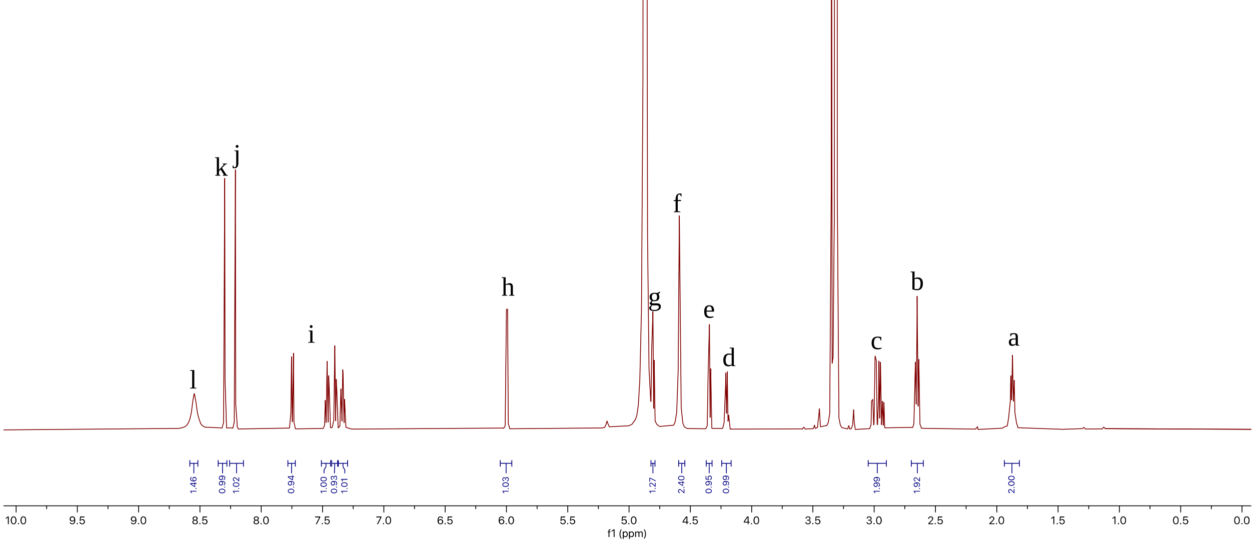
l
k
j
i
h
g
f
e
d
c
b
a
1.46
0.99
1.02
0.94
1.00
0.93
1.01
1.03
1.27
2.40
0.95
0.99
1.99
1.92
2.00
10.0
9.5
9.0
8.5
8.0
7.5
7.0
6.5
6.0
5.5
5.0
4.5
4.0
3.5
3.0
2.5
2.0
1.5
1.0
0.5
0.0
f1 (ppm)

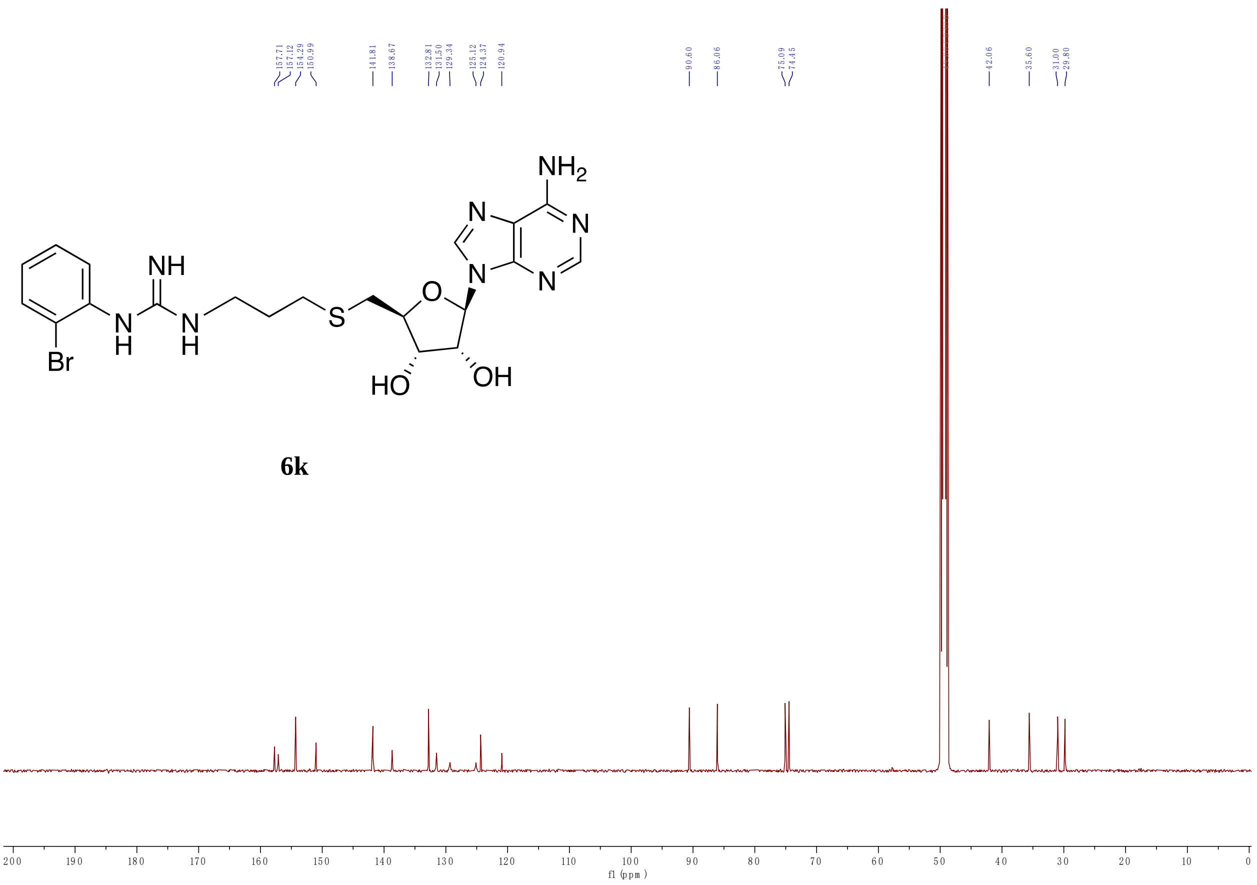

HRMS Spectra of 6k

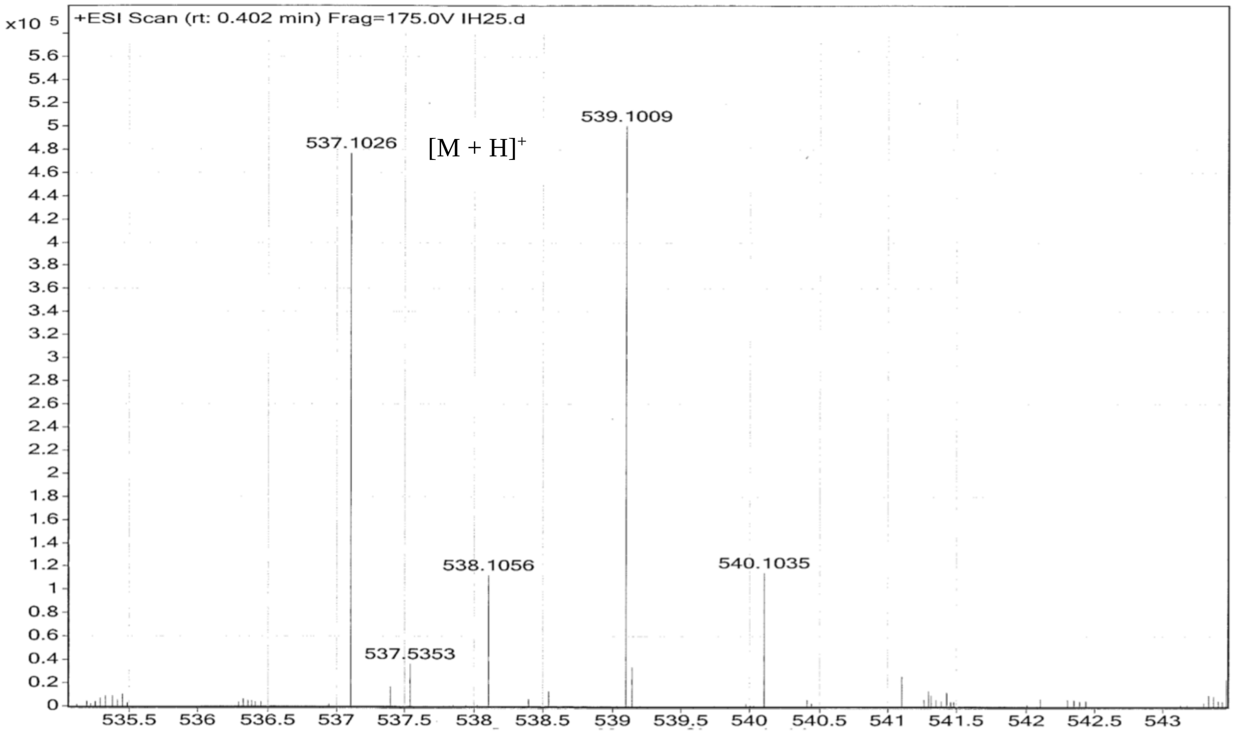

HPLC Spectra of 6k

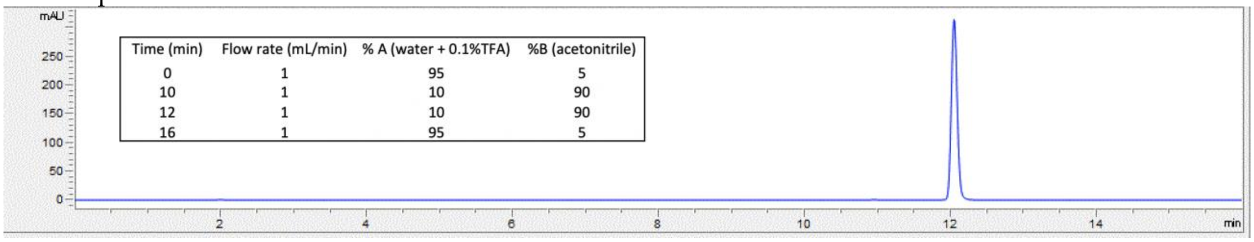

Time (min)	Flow rate (mL/min)	% A (water + 0.1%TFA)	%B (acetonitrile)
0	1	95	5
10	1	10	90
12	1	10	90
16	1	95	5

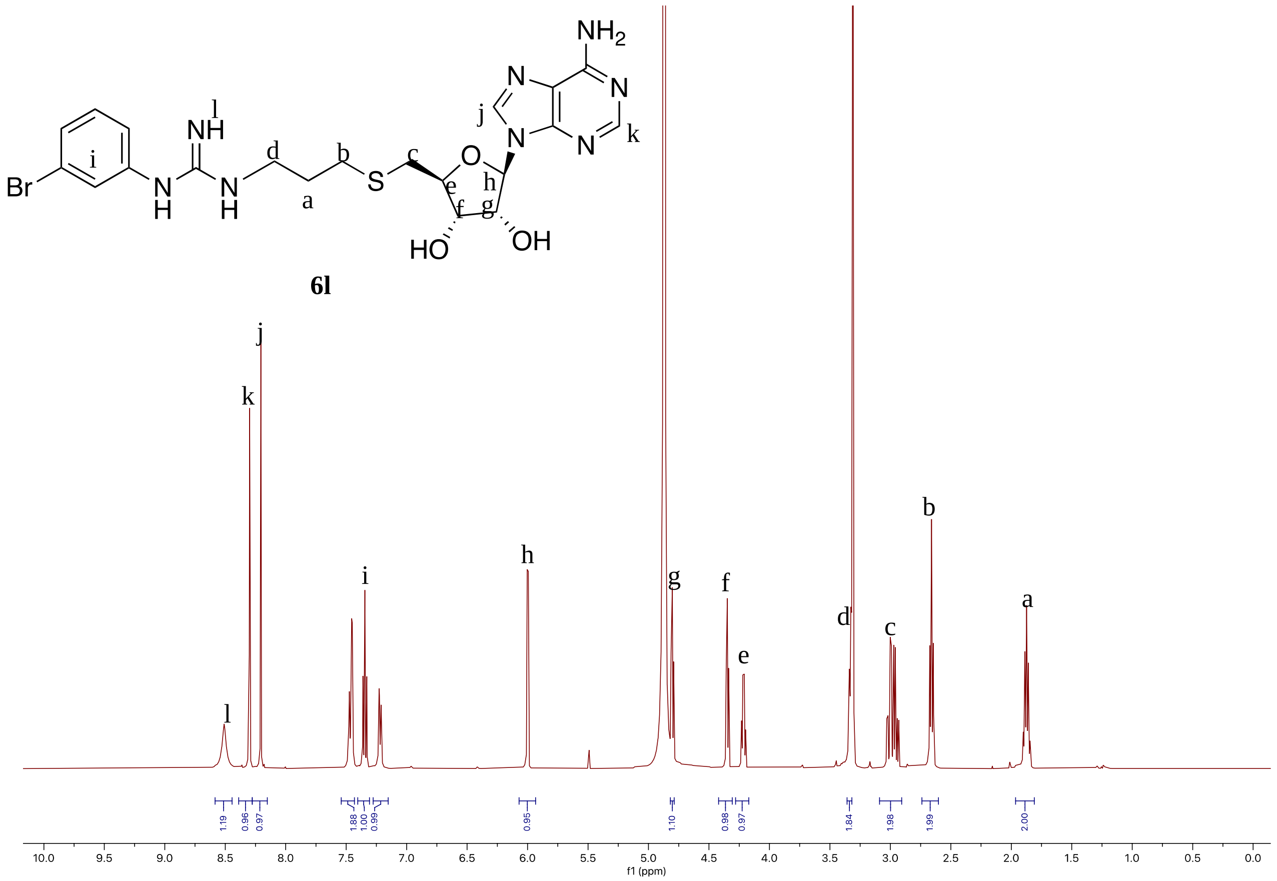

NH$_2$
N
N
N
N
j
k
l
NH
Br
i
N
H
N
H
d
b
a
c
S
O
e
h
f
g
HO
OH
l
k
j
i
h
g
f
e
d
c
b
a
1.19
0.96
0.97
1.88
1.00
0.99
0.95
1.10
0.98
0.97
1.84
1.98
1.99
2.00
10.0 9.5 9.0 8.5 8.0 7.5 7.0 6.5 6.0 5.5 5.0 4.5 4.0 3.5 3.0 2.5 2.0 1.5 1.0 0.5 0.0
f1 (ppm)

6l

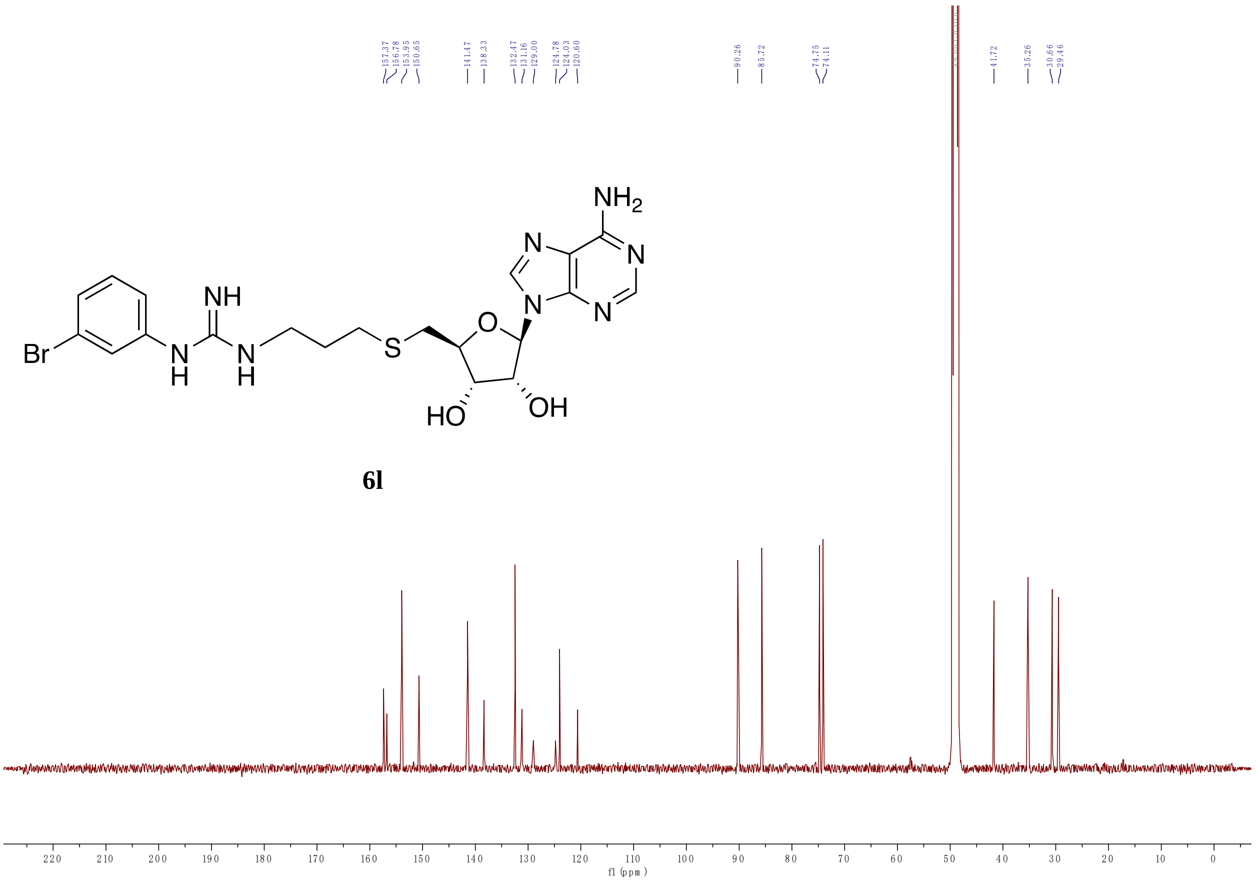

6l

HRMS Spectra of 6l

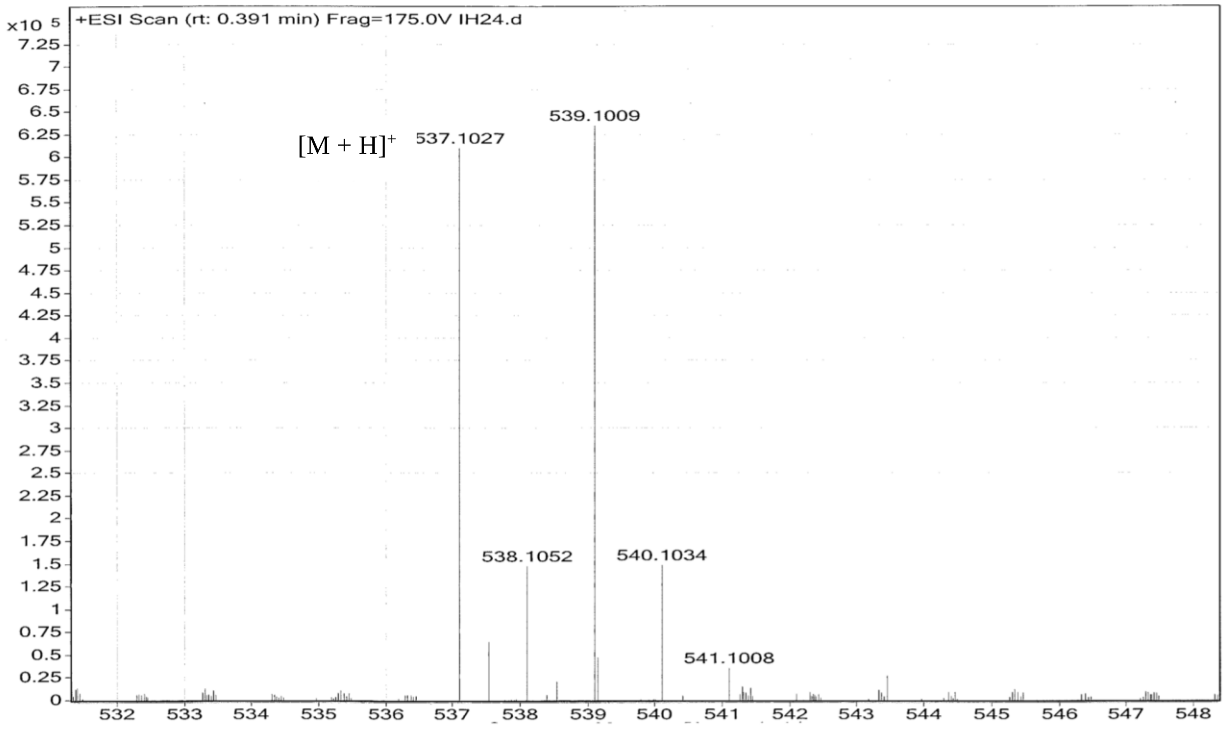

HPLC Spectra of 6l

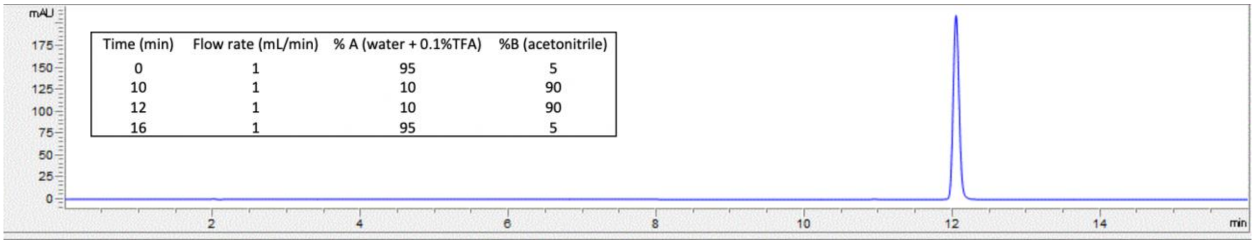

Time (min)	Flow rate (mL/min)	% A (water + 0.1%TFA)	%B (acetonitrile)
0	1	95	5
10	1	10	90
12	1	10	90
16	1	95	5

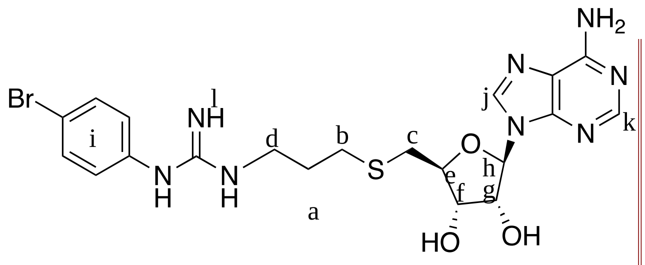

Br
i
NH
l
N
H
N
H
d
b
a
S
c
O
e
f
g
h
HO
OH
N
N
N
N
NH2
j
k

6m

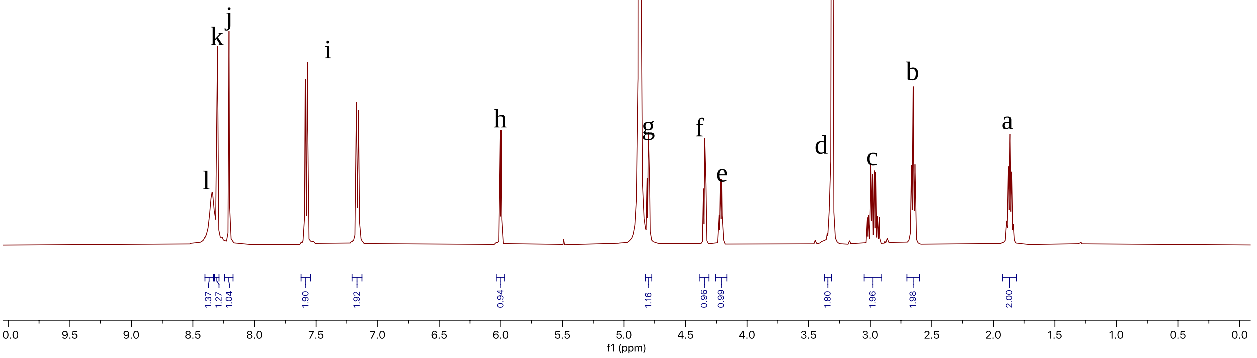

l
k
j
i
h
g
f
e
d
c
b
a
1.37
1.27
1.04
1.90
1.92
0.94
1.16
0.96
0.99
1.80
1.96
1.98
2.00
0.0
9.5
9.0
8.5
8.0
7.5
7.0
6.5
6.0
5.5
5.0
4.5
4.0
3.5
3.0
2.5
2.0
1.5
1.0
0.5
0.0
f1 (ppm)

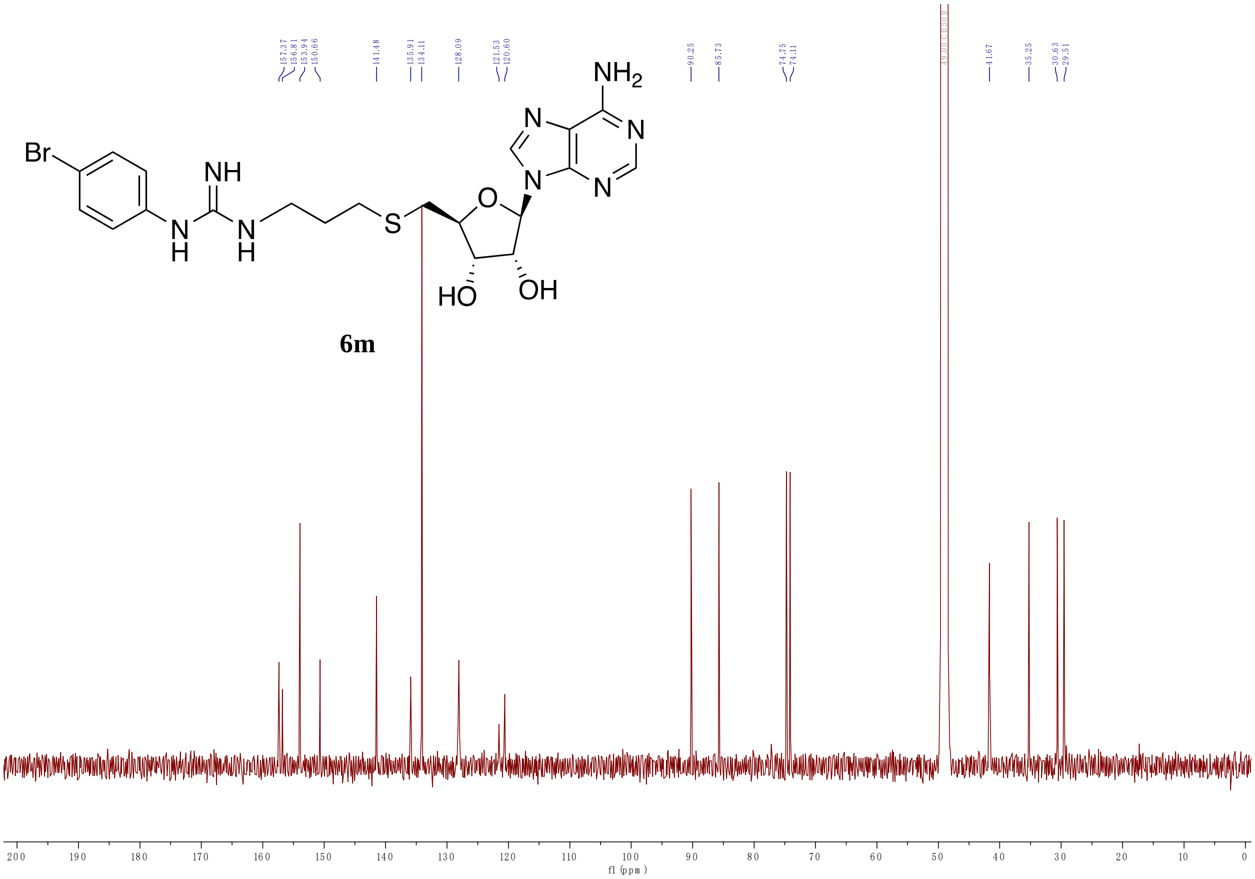

HRMS Spectra of 6m

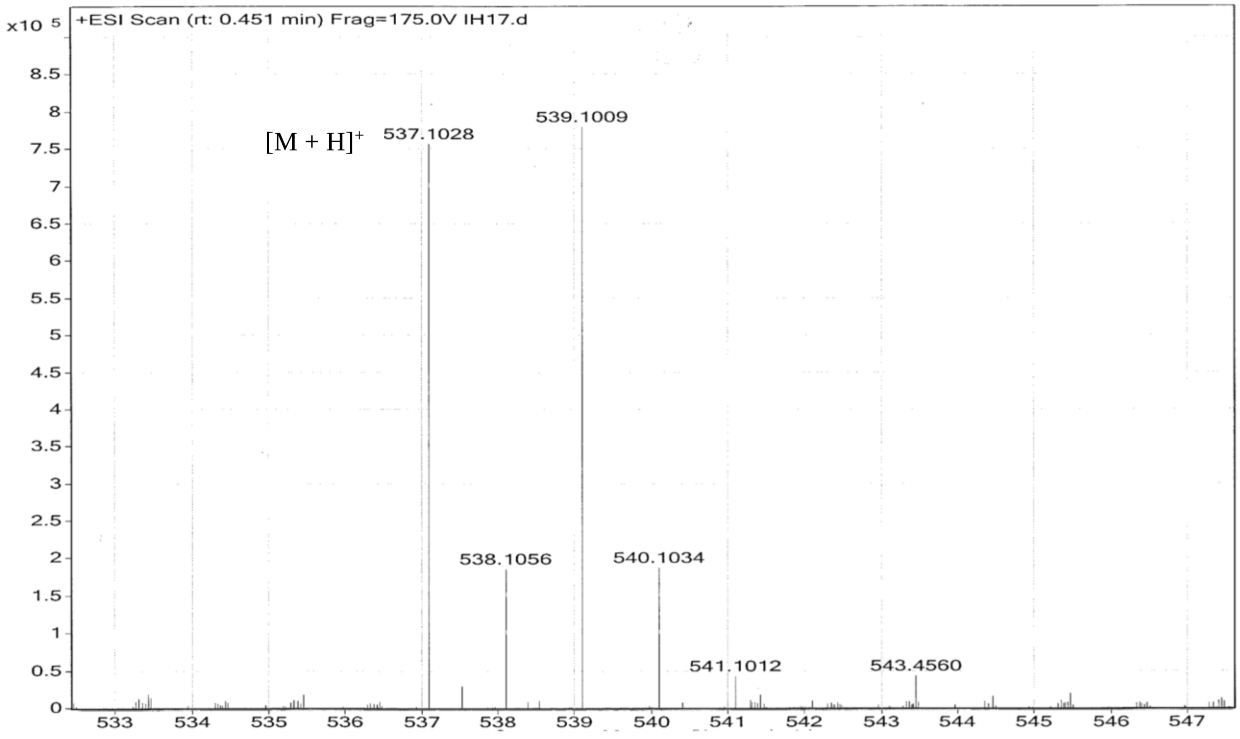

HPLC Spectra of 6m

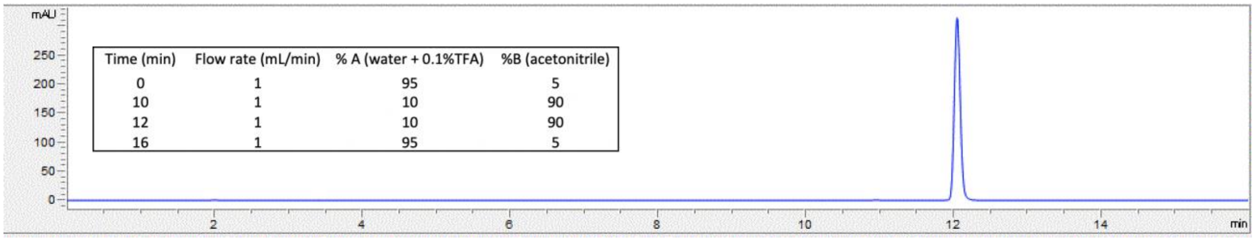

Time (min)	Flow rate (mL/min)	% A (water + 0.1%TFA)	%B (acetonitrile)
0	1	95	5
10	1	10	90
12	1	10	90
16	1	95	5

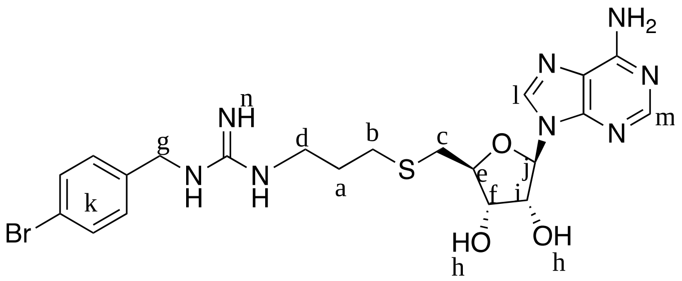

6n

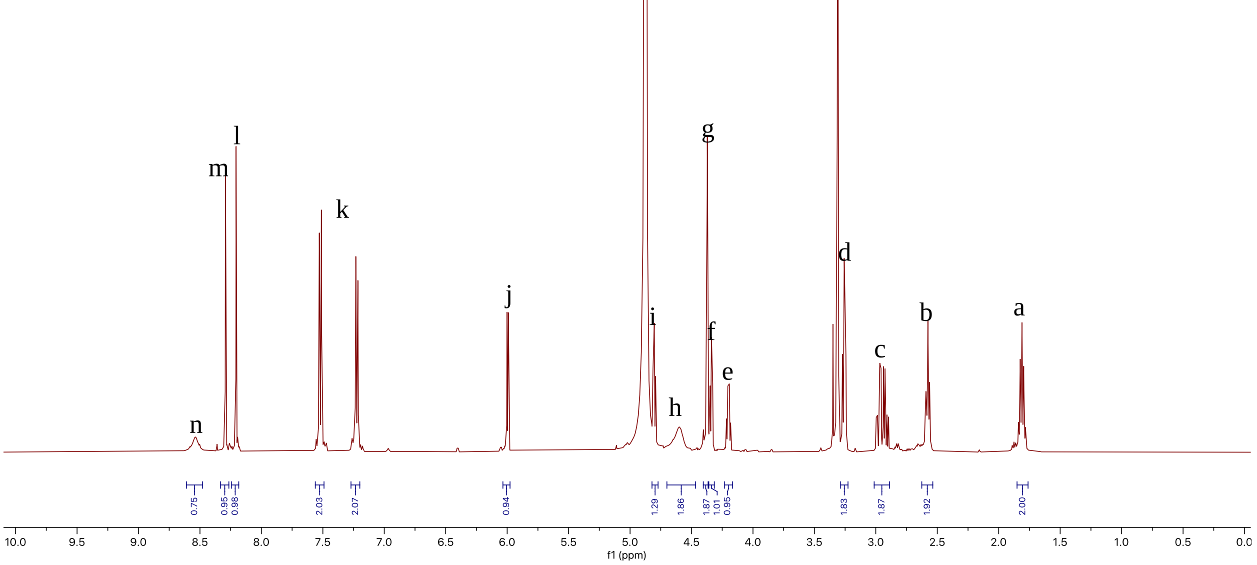
n
m
l
k
j
i
h
g
f
e
d
c
b
a
0.75
0.95
0.98
2.03
2.07
0.94
1.29
1.86
1.87
1.01
0.95
1.83
1.87
1.92
2.00
10.0 9.5 9.0 8.5 8.0 7.5 7.0 6.5 6.0 5.5 5.0 4.5 4.0 3.5 3.0 2.5 2.0 1.5 1.0 0.5 0.0
f1 (ppm)

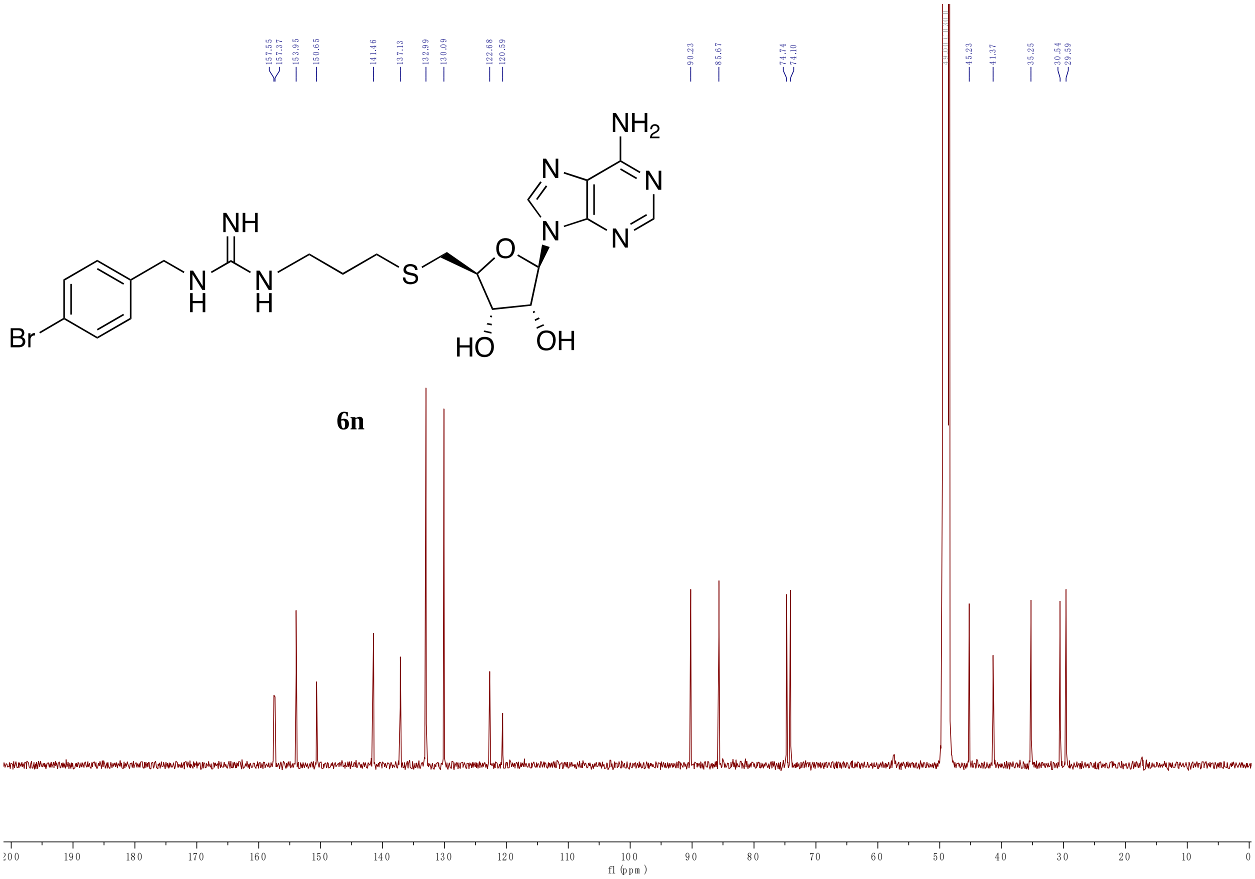

HRMS Spectra of 6n

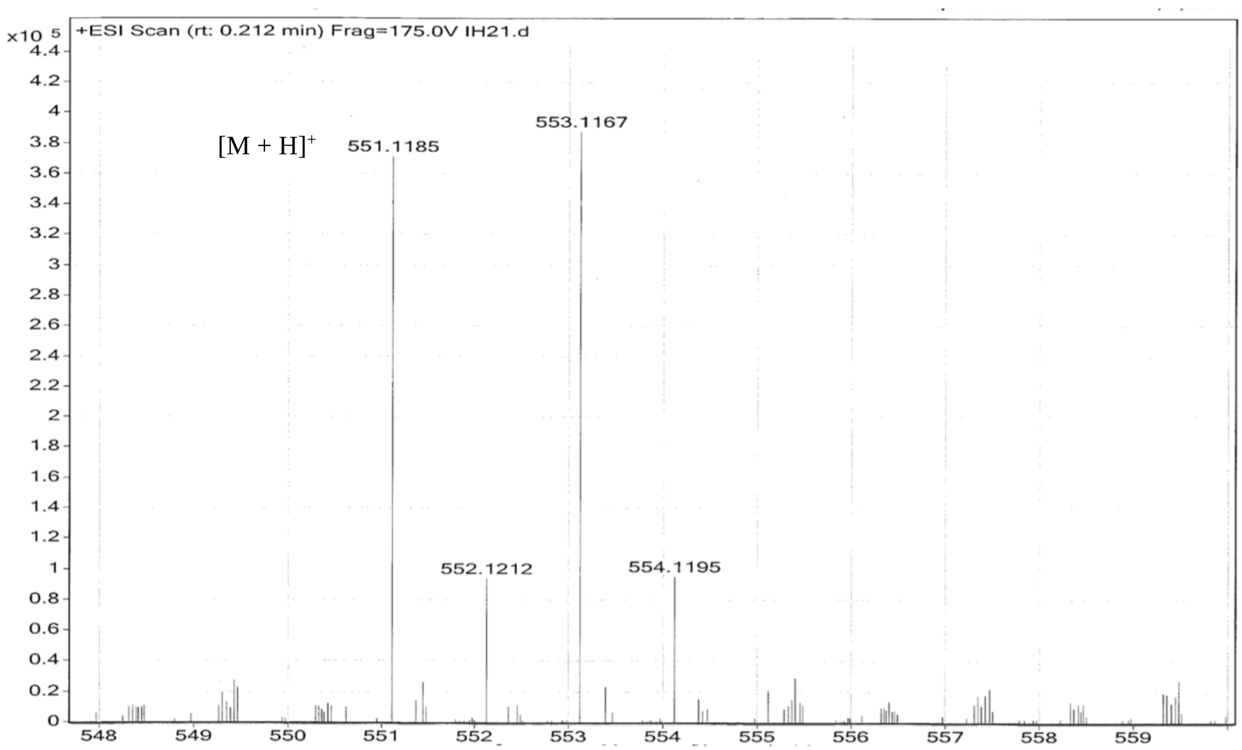

HPLC Spectra of 6n

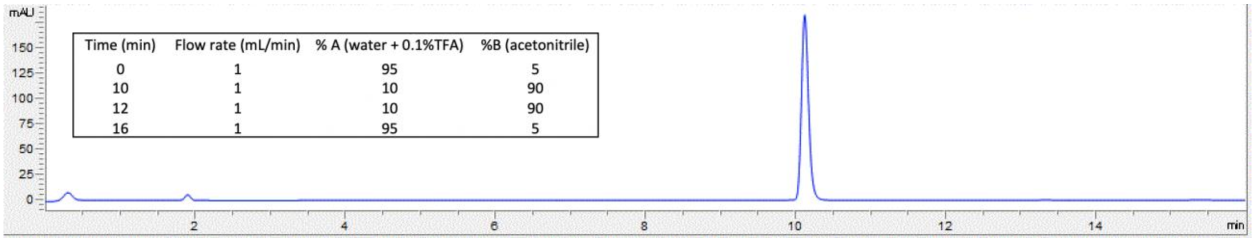

Supplement: Supplementary file 1 [file biomolecules-11-00854-s001.zip › biomolecules-1199650-supplementary.pdf]
